# 1,1,1,3,3,3-Hexafluoro-2-Propanol-Promoted Friedel–Crafts Reaction: Metal-Free Synthesis of C3-Difluoromethyl Carbinol-Containing Imidazo[1,2-*a*]pyridines at Room Temperature

**DOI:** 10.3390/molecules28227522

**Published:** 2023-11-10

**Authors:** Juanjuan Gao, Zhaowen Liu, Xiaohua Guo, Longhui Wu, Zhixi Chen, Kai Yang

**Affiliations:** College of Pharmacy, Gannan Medical University, Ganzhou 341000, China; gaoya0758@163.com (J.G.); liuzhaowenyifan@126.com (Z.L.); xhguo20041@163.com (X.G.); 13970997583@163.com (L.W.)

**Keywords:** hydroxydifluoromethylation, Friedel–Crafts reaction, imidazo[1,2-*a*]pyridine, HFIP

## Abstract

A facile and efficient method has been developed for the synthesis of C3-difluoromethyl carbinol-containing imidazo[1,2-*a*]pyridines at room temperature via the HFIP-promoted Friedel–Crafts reaction of difluoroacetaldehyde ethyl hemiacetal and imidazo[1,2-*a*]pyridines. This strategy could be applied to the direct C(sp^2^)-H hydroxydifluoromethylation of imidazo[1,2-*a*]pyridines and afford a series of novel difluoromethylated carbinols in good to satisfactory yields with 29 examples. Furthermore, gram-scale and synthetic transformation experiments have also been achieved, demonstrating its potential applicable value in organic synthesis. This green protocol has several advantages, including being transition metal- and oxidant-free, being carried out at room temperature, having high efficiency, and having a wide substrate scope.

## 1. Introduction

**Figure 1:**
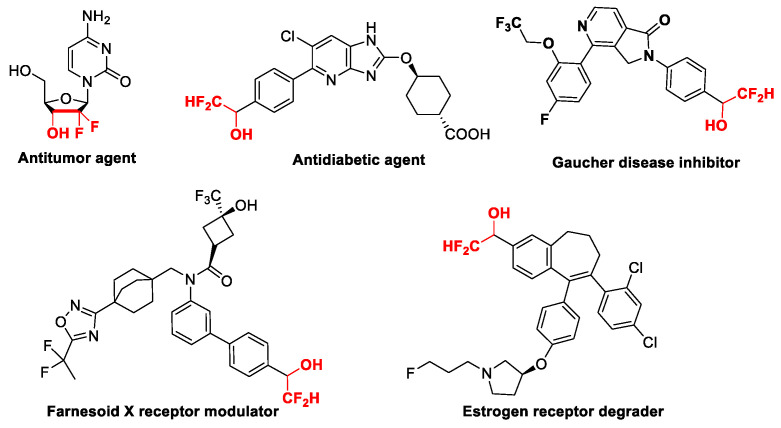
Representative difluoromethyl carbinol-containing biologically active molecules.

The incorporation of fluorine-containing groups within bioactive compounds is considered one of the most useful approaches to address important issues relevant to medicinal chemistry due to their unique chemical and biological properties, such as affinity, metabolic stability, lipophilicity, cell permeability, and bioavailability [1,2,3]. One of the more prominent examples is the CF_2_H unit, which is a good hydrogen bond donor and may serve as a bioisostere for hydroxy, thiol, and amide groups, and it has additional attractive properties such as its ability to modulate lipophilicity, polarity, and conformational preferences [4]. For these reasons, CF_2_H-containing compounds may be widely applied in the fields of pharmaceuticals, agrochemicals, and advanced functional materials [5,6,7]. Notably, difluoromethyl carbinols containing both the difluoromethyl and hydroxyl groups are prevalent in bioactive molecules, such as antitumor agents [8], antidiabetic agents [9], Gaucher disease inhibitors [10], farnesoid X receptor modulators [11], and estrogen receptor degraders [12] (Figure 1). Therefore, the exploration of a facile synthetic protocol of difluoromethylated carbinol derivatives is an undeniably important and valuable research topic in expanding the chemical space for drug discovery [13,14].

**Figure 2:**
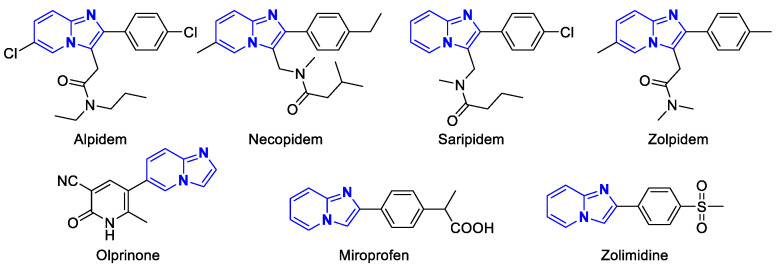
Representative drugs containing imidazo[1,2-*a*]pyridine scaffolds.

In addition, imidazo[1,2-*a*]pyridines are widely found in natural products. They are also extensively used in modern organic synthesis and pharmaceutical and materials science [15]. Imidazo[1,2-*a*]pyridine is considered to be an important scaffold due to its various biological and pharmaceutical activities, such as antiviral, antifungal, and antitumor activities. The core structure of many commercially available drugs includes alpidem, miroprofen, necopidem, olprinone, saripidem, zolpidem, and zolimidine (Figure 2) [16,17,18,19,20,21]. Therefore, the efficient and green preparation of imidazo[1,2-*a*]pyridine derivatives with various substituents has also drawn considerable attention, especially C3-functionalized imidazo[1,2-*a*]pyridine derivatives [22]. Therefore, a sustained effort is being made to develop new approaches to synthesize C3-functionalized imidazo[1,2-*a*]pyridine derivatives. However, there are no reports on the synthesis of C3-difluoromethyl carbinol-containing imidazo[1,2-*a*]pyridines.

**Scheme 1:**
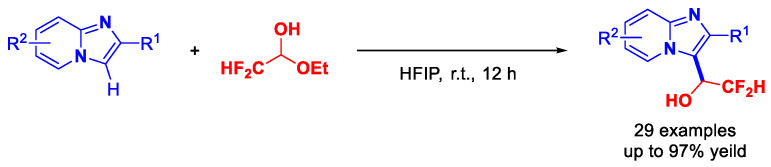
The synthesis of C3-difluoromethyl carbinol-containing imidazo[1,2-*a*]pyridines.

The strategy of C-H bond functionalization is known to be an ideal route for the preparation of diverse imidazo[1,2-*a*]pyridines as it is a straightforward, atom-economical, and synthetic step-economical method [23,24,25]. However, there are no reports on the C-H hydroxydifluoromethylation of imidazo[1,2-*a*]pyridines. It has been reported that 1,1,1,3,3,3-hexafluoro-2-propanol (HFIP), with a much higher polarity, increased Brønsted acidity, and strong hydrogen-bond donation [26,27,28,29,30,31], was considered to be a promising catalyst or promoter for the Friedel–Crafts reaction. For example, as early as 2012, the Naájera research group reported that HFIP serves as a promoter for the substitution reaction of allylic alcohols with nucleophiles [26]. Subsequently, HFIP was studied and applied as a catalyst or promoter in various types of Friedel–Crafts reactions. Inspired by this strategy for HFIP-promoted Friedel–Crafts alkylation, we focused our interest on developing facile methods for the synthesis of C3-difluoromethyl carbinol-containing imidazo[1,2-*a*]pyridines at room temperature via the HFIP-promoted cross-dehydrative coupling of difluoroacetaldehyde ethyl hemiacetal and imidazo[1,2-*a*]pyridines (Scheme 1). This green protocol possesses many intrinsic advantages like operational simplicity, high efficiency, atom economy, mild reaction conditions (e.g., at room temperature, transition metal- and oxidant-free, without inert gas protection), and a wide substrate scope.

## 2. Results and Discussion

### 2.1. Optimization of Reaction Conditions

The hydroxydifluoromethylation of imidazo[1,2-*a*]pyridine (**1a**) and commercially available difluoroacetaldehyde ethylacetal (**2a**) was selected as the model reaction for the optimization of the reaction conditions. The results are summarized in Table 1.

Initially, the effect of Brønsted acids and Lewis acids, such as trifluoroacetic acid (TFA), *p*-toluenesulfonic acid (TsOH), BF_3_**^.^**Et_2_O, Y(OTf)_3_, and Sc(OTf)_3_, employed as catalysts on the reaction was investigated (Entries 1–5); it was found that only a trace amount of product **3a** was detected. HFIP was considered to be a promising catalyst or promoter considering its much higher polarity, increased Brønsted acidity, and strong hydrogen-bond donation [26,27,28,29,30,31]. Therefore, we investigated the effect of HFIP as a catalyst or promoter on this transformation. Regrettably, trace product **3a** was afforded in dichloromethane when 10 mol% HFIP was employed in the reaction, indicating that the catalytic amount of HFIP was not enough to facilitate the hydroxydifluoromethylation process (Entry 6). We attempted to increase the dosage of HFIP to 1.0 or 2.0 equivalents; product **3a** could be obtained in 50% and 73% yields, respectively (Entries 7 and 8). This result encouraged us to use HFIP as a solvent for the reaction. Excitingly, the hydroxydifluoromethylation could proceed completely to obtain **3a** in 97% yield (Entry 9). Thus, using HFIP as the solvent, the reaction of **1a** (0.2 mmol) and **2a** (0.4 mmol) was successfully achieved at room temperature for 12 h to obtain **3a** in satisfactory yield.

### 2.2. Scope of Reaction Substrates

Under the optimized reaction conditions, the substrate scope of this approach was examined by using different 2-substituted imidazo[1,2-*a*]pyridines. The results are summarized in Table 2.

It was found that 2-phenyl imidazo[1,2-*a*]pyridine could react smoothly with **2a** to give the corresponding product **3b** in 95% yield. Then, a series of substituents, such as methyl, methoxyl, fluoro, chloro, and bromo groups on the C2-phenyl ring of 2-phenyl imidazo[1,2-*a*]pyridines, were well tolerated under the standard conditions. This showed that both methyl and methoxyl substituted substrates proceeded smoothly to give the corresponding products **3c**–**3f** in 79–92% yields. The halo-substituted substrates exhibited good reactivity in the hydroxydifluoromethylation to obtain products **3g**–**3l** in 82–92% yields. Dichloro-substituted and dimethoxy-substituted substrates were also subjected to smooth transformation to afford products **3m** and **3n** in 84% and 82% yields, respectively. As anticipated, 2-(naphthalen-1-yl) and 2-(naphthalen-2-yl) imidazo[1,2-*a*]pyridines underwent hydroxydifluoromethylation to give the desired products **3o** and **3p** in 78% and 80% yields, respectively. To our delight, 2-heteroaryl imidazo[1,2-*a*]pyridines were also successfully employed for the synthesis of the desired products. Using 2-(2-pyridyl)imidazo[1,2-*a*]pyridine and 2-(2-thienyl)imidazo[1,2-*a*]pyridine as substrates, corresponding products **3q** and **3r** were obtained in 68% and 73% yields, respectively. Furthermore, this conversion could be readily carried out on the 5 mmol scale to obtain the desired product **3a** in 87% yield, which proved to be easily applied to a gram-scale preparation.

To further extend the scope of this methodology, a series of imidazo[1,2-*a*]pyridines with different substituents on the pyridine ring were used to evaluate the universality of this hydroxydifluoromethylation (Table 3). As anticipated, imidazo[1,2-*a*]pyridines with an electron-donating group as well as a halogen group on the pyridine rings all worked well with **2a** and afforded the desired target molecules **3aa**–**3ah** in satisfactory yields (83–95%). Trifluoromethyl, as a strong electron-withdrawing group, can also be tolerated in this conversion, giving the products in good yields (**3ai**, 89% and **3aj**, 87%). 6-Bromo-7-methyl-2-phenylimidazo[1,2-*a*]pyridine as a polysubstituted substrate was also suitable for this conversion, resulting in the product **3ak** with 90% yield.

The structures of the desired products were confirmed by NMR (^1^H, ^13^C, and ^19^F) and HM-RS data, and the structure of **3b** (CCDC 2295549) was unambiguously confirmed by single-crystal X-ray analysis, which can be seen in Supplementary Materials for details [32].

### 2.3. Mechanism Investigation

**Scheme 2:**
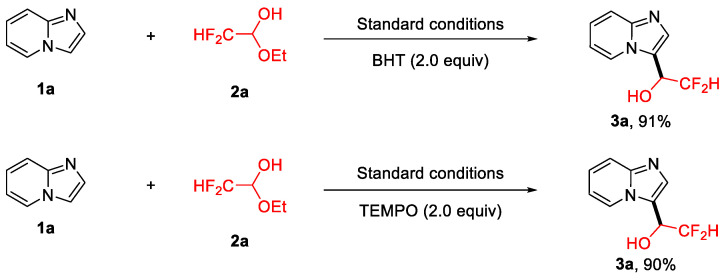
Control experiments.

To gain insight into the details of the mechanism, we performed two control experiments accordingly. Adding 2.0 equivalent radical scavenger 2,2,6,6-tetramethylpiperidin-1-yloxyl (TEMPO) or BHT (butylated hydroxytoluene) to the reaction system, it was found that the corresponding compound **3a** could also be obtained in 91% and 88% isolation yields, respectively (Scheme 2). These results indicated that the reaction may not be involved in the pathway of radical participation.

**Scheme 3:**
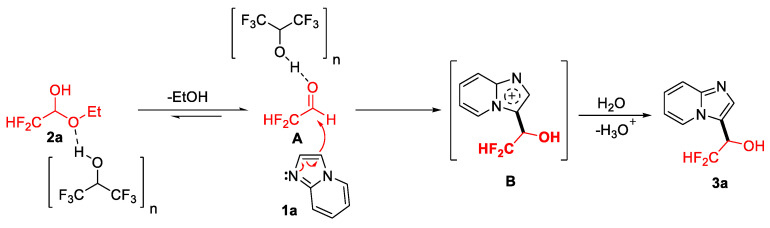
A plausible reaction pathway.

It has been reported that HFIP can be used to promote the generation of a C-C bond by cleavage of a C-O bond (the substrates of aromatic aldehyde hydrate, propargyl alcohol, difluoroacetaldehyde ethyl hemiacetal, etc.) via the Friedel–Crafts alkylation pathway [26,27,28,33,34,35]. Based on the properties of HFIP and the above-mentioned control experimental results, a plausible mechanism of HFIP-promoted Friedel–Crafts alkylation was put forward (Scheme 3). Initially, under the action of proton donor HFIP, the difluoroacetaldehyde ethyl hemiacetal **2a** has a tendency to remove one molecule of ethanol to generate difluoroacetaldehyde **A,** then with proton exchange with HFIP taking place and forming carbonium ions to active difluoroacetaldehyde **A**, making it more susceptible for nucleophilic addition with imidazo[1,2-*a*]pyridine **1a**, to generate the corresponding **B**. Finally, **B** undergoes the dehydrogenation process to produce the final product **3a**.

### 2.4. Synthetic Derivatization

**Scheme 4:**
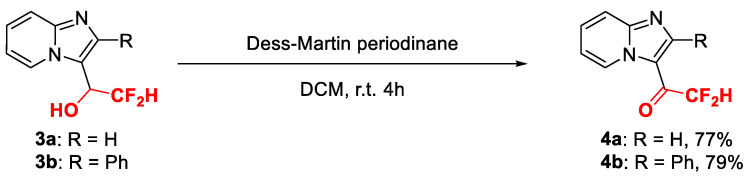
Synthetic derivatization.

Moreover, the reactions are highly practical because of their wide range of applications in pharmaceutical chemistry for the production of diversified structural scaffolds and combinatorial libraries for drug discovery. The desired targets derivatized at hydroxyl positions may be promising candidates for such applications in pharmaceutical chemistry and organic synthesis. For instance, C3-difluoroacetyl imidazo[1,2-*a*]pyridines **4a** and **4b** have been prepared through Dess–Martin periodinane oxidation with **3a** and **3b** with yields of 77% and 79%, respectively (Scheme 4).

## 3. Materials and Methods

### 3.1. General Information

Melting point (m.p.) was performed on a Büchi Melting Point B-545 instrument without correcting. The ^1^H, ^13^C and ^19^F NMR spectra were collected on a BRUKER DRX-400 spectrometer in CDCl_3_ using tetramethylsilane (TMS) as an internal standard. High-resolution mass spectra (HRMS) were obtained with an LCMS-IT-TOF mass spectrometer. Single-crystal X-ray analysis was obtained using Bruker APEX2 Smart CCD. TLC was performed by using commercially prepared 100–400 mesh silica gel plates (GF254), and visualization was detected at 254 or 365 nm. All reagents and solvents were purchased from commercial sources and used without further purification, The 2-substituted imidazo[1,2-*a*]pyridines **1** (except for **1a**) were synthesized from 2-bromoacetophenones (or acetophenones) and various 2-aminopyridines [36,37].

### 3.2. Experimental Procedure for Compounds ***3a***–***3ak***

The mixture of substituted imidazo[1,2-*a*]pyridine **1** (0.2 mmol, 1.0 equiv.) and difluoroacetaldehyde ethyl hemiacetal **2a** (0.30 mmol, 1.5 equiv.) in 1,1,1,3,3,3-hexafluoro-2-propanol (1.0 mL) was stirred at room temperature for 12 h. After the completion of the reaction, the reaction mixture was quenched with H_2_O (15 mL) and extracted three times with ethyl acetate (3 × 15 mL). Then, the organic layer was dried over anhydrous Na_2_SO_4_. After the filtration and evaporation of the solvents under reduced pressure, the crude products were purified by column chromatography on silica gel to afford the desired products **3a**–**3ak**.

### 3.3. Experimental Procedure for Compounds ***4a*** and ***4b***

Compounds **3a** or **3b** (0.1 mmol, 1.0 equiv.) and Dess–Martin periodinane (0.37 mmol, 3.7 equiv.) in DCM (1.0 mL) were stirred in a ground glass test tube at room temperature for 4 h. After monitoring the end of the reaction on TLC, the residues were purified by column chromatography on silica gel to give the pure products **4a** or **4b**.

### 3.4. Characterization Data for All Products ***3a***–***3ak*** and ***4a***–***4b***

*(±)-3-(2,2-Difluoro-1-hydroxy)ethyl-imidazo[*1,2-*a]pyridine* (**3a**), white solid (38 mg, 97%); m.p. 160–162 °C; ^1^H-NMR (400 MHz, CDCl_3_:CD_3_OD = 7:1), *δ_H_*: 5.16–5.23 (*m*, 1H), 6.10 (*td*, *J* = 55.6, 3.2 Hz, 1H), 6.85–6.90 (*m*, 1H), 7.23–7.28 (*m*, 1H), 7.49–7.56 (*m*, 2H), 8.41 (*d*, *J* = 6.8 Hz, 1H); ^13^C-NMR (100 MHz, CDCl_3_:CD_3_OD = 7:1), *δ_C_*: 65.7 (*t*, *J* = 26.0 Hz), 112.7, 115.1 (*t*, *J* = 243.0 Hz), 116.9, 119.9, 125.6, 125.7, 131.8, 146.2; ^19^F NMR (376 MHz, CDCl_3_:CD_3_OD = 7:1), *δ_F_*, ppm: −126.14 (*d*, *J* = 285.8 Hz, 1F), 127.05 (*d*, *J* = 285.8 Hz, 1F); ESI-HRMS, *m*/*z*: Calcd for C_9_H_9_F_2_N_2_O [M + H]^+^, 199.0677, found: 199.0669.
*(±)-3-(2,2-Difluoro-1-hydroxy)ethyl-2-phenyl-imidazo[*1,2-*a]pyridine* (**3b**), white solid (52 mg, 95%); m.p. 161–163 °C; ^1^H-NMR (400 MHz, CDCl_3_:CD_3_OD = 7:1), *δ_H_*, ppm: 5.31–5.38 (*m*, 1H), 6.14 (*td*, *J* = 55.6, 3.2 Hz, 1H), 7.18–7.23 (*m*, 1H), 7.30–7.35 (*m*, 3H), 7.44–7.50 (*m*, 3H), 8.63 (*d*, *J* = 6.8 Hz, 1H); ^13^C-NMR (100 MHz, CDCl_3_:CD_3_OD = 7:1), *δ_C_*, ppm: 66.2 (*t*, *J* = 25.0 Hz), 112.3, 115.4 (*t*, *J* = 245.0 Hz), 115.6, 116.5, 125.9, 127.3, 128.3, 128.4, 128.8, 133.1, 145.4, 145.5; ^19^F NMR (376 MHz, CDCl_3_:CD_3_OD = 7:1), *δ_F_*, ppm: −123.70 (*d*, *J* = 282.0 Hz, 1F), −124.88 (*d*, *J* = 282.0 Hz, 1F); ESI-HRMS, *m*/*z*: Calcd for C_15_H_13_F_2_N_2_O [M + H]^+^, 275.0990, found: 275.0979.
*(±)-3-(2,2-Difluoro-1-hydroxy)ethyl-2-(p-tolyl)-imidazo[*1,2-*a]pyridine* (**3c**), white solid (53 mg, 92%); m.p. 187–189 °C; ^1^H-NMR (400 MHz, CDCl_3_:CD_3_OD = 7:1), *δ_H_*, ppm: 2.35 (*s*, 3H), 5.31–5.38 (*m*, 1H), 6.13 (*J* = 55.6, 3.2 Hz, 1H), 6.75–6.80 (*m*, 1H), 7.13 (*d*, *J* = 7.6 Hz, 2H), 7.16–7.21 (*m*, 1H), 7.35 (*d*, *J* = 8.0 Hz, 2H), 7.43 (*d*, *J* = 9.2 Hz, 1H), 8.61 (*d*, *J* = 6.8 Hz, 1H); ^13^C-NMR (100 MHz, CDCl_3_:CD_3_OD = 7:1), *δ_C_*, ppm: 21.2, 66.2 (*t*, *J* = 24.6 Hz), 112.2, 115.4 (*t*, *J* = 244.0 Hz), 116.4, 125.8, 127.3, 128.6, 128.8, 129.1, 129.3, 130.1, 138.1, 145.4; ^19^F NMR (376 MHz, CDCl_3_:CD_3_OD = 7:1), *δ_F_*, ppm: −123.68 (*d*, *J* = 282.0 Hz, 1F), −124.96 (*d*, *J* = 282.0 Hz, 1F); ESI-HRMS, *m*/*z*: Calcd for C_16_H_15_F_2_N_2_O [M + H]^+^, 289.1147, found: 289.1136.
*(±)-3-(2,2-Difluoro-1-hydroxy)ethyl-2-(4-methoxyphenyl)-imidazo[*1,2-*a]pyridine* (**3d**), white solid (55 mg, 90%); m.p. 178–180 °C; ^1^H-NMR (400 MHz, CDCl_3_:CD_3_OD = 7:1), *δ_H_*, ppm: 3.83 (*s*, 3H), 5.29–5.36 (*m*, 1H), 6.14 (*td*, *J* = 55.6, 2.4 Hz, 1H), 6.71–6.81 (*m*, 1H), 6.86–6.90 (*m*, 2H), 7.81–7.21 (*m*, 1H), 7.40–7.46 (*m*, 3H), 8.62 (*d*, *J* = 4.0 Hz, 1H); ^13^C-NMR (100 MHz, CDCl_3_:CD_3_OD = 7:1), *δ_C_*, ppm: 55.2, 66.2 (*t*, *J* = 25.0 Hz), 112.2, 113.9, 115.1, 115.4 (*t*, *J* = 244.0 Hz), 116.3, 115.6, 125.8, 127.2, 130.1, 145.2, 145.4, 159.6; ^19^F NMR (376 MHz, CDCl_3_:CD_3_OD = 7:1), *δ_F_*, ppm: −123.74 (*d*, *J* = 282.0 Hz, 1F), −124.87 (*d*, *J* = 282.0 Hz, 1F); ESI-HRMS, *m*/*z*: Calcd for C_16_H_15_F_2_N_2_O_2_ [M + H]^+^, 305.1096, found: 305.1081.
*(±)-3-(2,2-Difluoro-1-hydroxy)ethyl-2-(3-methoxyphenyl)-imidazo[*1,2-*a]pyridine* (**3e**), white solid (52 mg, 85%); m.p. 176–178 °C; ^1^H-NMR (400 MHz, CDCl_3_:CD_3_OD = 7:1), *δ_H_*, ppm: 5.34–5.42 (*m*, 1H), 6.13 (*td*, *J* = 55.6, 3.6 Hz, 1H), 6.75–6.79 (*m*, 1H), 6.84–6.87 (*m*, 1H), 7.00–7.05 (*m*, 2H), 7.14–7.23 (*m*, 2H), 7.41 (*d*, *J* = 7.2 Hz, 1H), 8.61 (*d*, *J* = 6.8 Hz, 1H); ^13^C-NMR (100 MHz, CDCl_3_:CD_3_OD = 7:1), *δ_C_*, ppm: 55.2, 66.3 (*t*, *J* = 26.0 Hz), 112.3, 112.9, 114.1, 114.2, 115.4 (*t*, *J* = 245.0 Hz), 115.5, 116.6, 1221.2, 125.9, 127.3, 129.5, 134.4, 145.3, 145.4, 159.4; ^19^F NMR (376 MHz, CDCl_3_:CD_3_OD = 7:1), *δ_F_*, ppm: −123.62 (*d*, *J* = 282.0 Hz, 1F), −124.75 (*d*, *J* = 282.0 Hz, 1F); ESI-HRMS, *m*/*z*: Calcd for C_16_H_15_F_2_N_2_O_2_ [M + H]^+^, 305.1096, found: 305.1081.
*(±)-3-(2,2-Difluoro-1-hydroxy)ethyl-2-(2-methoxyphenyl)-imidazo[*1,2-*a]pyridine* (**3f**), white solid (48 mg, 79%); m.p. 177–179 °C; ^1^H-NMR (400 MHz, CDCl_3_), *δ_H_*, ppm: 3.56 (*s*, 1H), 5.07–5.14 (*m*, 1H), 5.91 (*td*, *J* = 55.6, 1.6 Hz, 1H), 6.70–6.77 (*m*, 2H), 6.81 (*d*, *J* = 8.4 Hz, 1H), 7.09–7.16 (*m*, 2H), 7.21–7.25 (*m*, 1H), 7.37 (*d*, *J* = 8.8 Hz, 1H), 8.57 (*d*, *J* = 6.8 Hz, 1H); ^13^C-NMR (100 MHz, CDCl_3_), *δ_C_*, ppm: 55.2, 66.5 (*t*, *J* = 24.0 Hz), 111.0, 112.1, 115.7 (*t*, *J* = 246.0 Hz),, 116.6, 116.9, 120.8, 122.1, 125.4, 127.2, 130.0, 132.1, 141.3, 145.6, 156.3; ^19^F NMR (376 MHz, CDCl_3_), *δ_F_*, ppm: −123.06 (*d*, *J* = 278.2 Hz, 1F), −127.67 (*d*, *J* = 278.2 Hz, 1F); ESI-HRMS, *m*/*z*: Calcd for C_16_H_15_F_2_N_2_O_2_ [M + H]^+^, 305.1096, found: 305.1081.
*(±)-3-(2,2-Difluoro-1-hydroxy)ethyl-2-(4-fluorophenyl)-imidazo[*1,2-*a]pyridine 2,2-* (**3g**), white solid (49 mg, 85%); m.p. 208–210 °C; ^1^H-NMR (400 MHz, CD_3_OD), *δ_H_*, ppm: 5.28–5.35 (*m*, 1H), 6.33 (*td*, *J* = 55.6, 3.6 Hz, 1H), 6.92–6.96 (*m*, 1H), 7.18–7.24 (*m*, 2H), 7.35–7.39 (*m*, 1H), 7.55 (*d*, *J* = 9.2 Hz, 1H), 7.63–7.67 (*m*, 2H), 8.75 (*d*, *J* = 6.8 Hz, 1H); ^13^C-NMR (100 MHz, CD_3_OD), *δ_C_*, ppm: 66.1 (*t*, *J* = 25.0 Hz), 112.3, 115.1 (*t*, *J* = 22.0 Hz), 115.7 (*t*, *J* = 243.0 Hz), 115.8, 116.1, 126.4, 127.5, 129.6 (*d*, *J* = 3.0 Hz), 130.8 (*d*, *J* = 9.0 Hz), 145.5, 144.2, 163.0 (*d*, *J* = 246.0 Hz); ^19^F NMR (376 MHz, CDCl_3_:CD_3_OD = 7:1), *δ_F_*, ppm: −115.40, −125.45 (*d*, *J* = 285.8 Hz, 1F), −126.28 (*d*, *J* = 282.0 Hz, 1F); ESI-HRMS, *m*/*z*: Calcd for C_15_H_12_F_3_N_2_O [M + H]^+^, 293.0896, found: 293.0885.
*(±)-3-(2,2-Difluoro-1-hydroxy)ethyl-2-(2-fluorophenyl)-imidazo[*1,2-*a]pyridine* (**3h**), white solid (48 mg, 82%); m.p. 173–175 °C; ^1^H-NMR (400 MHz, CDCl_3_), *δ_H_*, ppm: 5.04–5.11 (*m*, 1H), 5.95 (*td*, *J* = 55.6, 2.0 Hz, 1H), 6.68–6.72 (*m*, 1H), 6.92–7.00 (*m*, 2H), 7.09–7.13 (*m*, 1H), 7.17–7.23 (*m*, 1H), 7.26–7.31 (*m*, 1H), 8.37 (*d*, *J* = 9.2 Hz, 1H), 8.57 (*d*, *J* = 6.0 Hz, 1H); ^13^C-NMR (100 MHz, CDCl_3_), *δ_C_*, ppm: 66.4 (*t*, *J* = 24.0 Hz), 112.4, 115.4 (*t*, *J* = 246.0 Hz), 115.6, 115.8, 116.6, 120.9 (*d*, *J* = 15.0 Hz), 124.3 (*d*, *J* = 4.0 Hz), 126.0, 127.5, 130.4 (*d*, *J* = 8.0 Hz), 132.0, 138.9, 145.9, 159.4 (*d*, *J* = 245.0 Hz); ^19^F NMR (376 MHz, CDCl_3_), *δ_F_*, ppm: −123.88 (*d*, *J* = 285.8 Hz, 1F), −124.66 (*d*, *J* = 285.8 Hz, 1F); ESI-HRMS, *m*/*z*: Calcd for C_15_H_12_F_3_N_2_O [M + H]^+^, 293.0896, found: 293.0885.
*(±)-2-(4-Chlorophenyl)-3-(2,2-difluoro-1-hydroxy)ethyl-imidazo[*1,2-*a]pyridine* (**3i**), white solid (55 mg, 90%); m.p. 198–200 °C; ^1^H-NMR (400 MHz, CDCl_3_:CD_3_OD = 7:1), *δ_H_*, ppm: 5.26–5.32 (*m*, 1H), 6.16 (*td*, *J* = 55.6, 3.2 Hz, 1H), 6.80–6.85 (*m*, 1H), 7.22–7.31 (*m*, 3H), 7.40 (*d*, *J* = 8.0 Hz, 2H), 7.46 (*d*, *J* = 9.2 Hz, 1H), 8.60 (*d*, *J* = 6.8 Hz, 1H); ^13^C-NMR (100 MHz, CDCl_3_:CD_3_OD = 7:1), *δ_C_*, ppm: 66.3 (*t*, *J* = 26.0 Hz), 112.6, 115.2 (*t*, *J* = 245.0 Hz), 115.6, 116.6, 126.2, 127.3, 128.7, 129.9, 131.5, 134.4, 144.2, 145.5; ^19^F NMR (376 MHz, CDCl_3_:CD_3_OD = 7:1), *δ_F_*, ppm: −123.74 (*d*, *J* = 282.0 Hz, 1F), −124.57 (*d*, *J* = 282.0 Hz, 1F); ESI-HRMS, *m*/*z*: Calcd for C_15_H_12_ClF_2_N_2_O [M + H]^+^, 309.0601, found: 309.0616.
*(±)-2-(3-Chlorophenyl)-3-(2,2-difluoro-1-hydroxy)ethyl-imidazo[*1,2-*a]pyridine* (**3j**), white solid (57 mg, 92%); m.p. 174–176 °C; ^1^H-NMR (400 MHz, CDCl_3_:CD_3_OD = 7:1), *δ_H_*, ppm: 5.28–5.26 (*m*, 1H), 6.15 (*td*, *J* = 55.6, 3.6 Hz, 1H), 6.78–6.83 (*m*, 1H), 7.20–7.23 (*m*, 1H), 7.25–7.32 (*m*, 2H), 7.35–7.38 (*m*, 1H), 7.44–7.51 (*m*, 2H), 8.63 (*d*, *J* = 6.8 Hz, 1H); ^13^C-NMR (100 MHz, CDCl_3_:CD_3_OD = 7:1), *δ_C_*, ppm: 66.1 (*t*, *J* = 25.0 Hz), 112.6, 115.3 (*t*, *J* = 245.0 Hz), 115.9, 116.5, 126.2, 126.9, 127.4, 128.3, 128.7, 129.7, 134.3, 134.9, 143.8, 145.5; ^19^F NMR (376 MHz, CDCl_3_:CD_3_OD = 7:1), *δ_F_*, ppm: −123.86 (*d*, *J* = 285.8 Hz, 1F), −124.63 (*d*, *J* = 285.8 Hz, 1F); ESI-HRMS, *m*/*z*: Calcd for C_15_H_12_ClF_2_N_2_O [M + H]^+^, 309.0601, found: 309.0616.
*(±)-2-(4-Bromophenyl)-3-(2,2-difluoro-1-hydroxy)ethyl-imidazo[*1,2-*a]pyridine* (**3k**), white solid (60 mg, 86%); m.p. 190–192 °C; ^1^H-NMR (400 MHz, CDCl_3_:CD_3_OD = 7:1), *δ_H_*, ppm: 5.24–5.31 (*m*, 1H), 6.16 (*td*, *J* = 55.6, 3.6 Hz, 1H), 6.80–6.85 (*m*, 1H), 7.22–7.26 (*m*, 1H), 7.32 (*d*, *J* = 8.4 Hz, 2H), 7.43–7.47 (*m*, 3H), 8.59 (*d*, *J* = 6.8 Hz, 1H); ^13^C-NMR (100 MHz, CDCl_3_:CD_3_OD = 7:1), *δ_C_*, ppm: 66.2 (*t*, *J* = 25.0 Hz), 112.5, 115.2 (*t*, *J* = 245.0 Hz), 115.7, 116.6, 122.7, 126.2, 127.3, 130.2, 131.6, 131.9, 144.2, 145.5; ^19^F NMR (376 MHz, CDCl_3_:CD_3_OD = 7:1), *δ_F_*, ppm: −123.74 (*d*, *J* = 282.0 Hz, 1F), −124.56 (*d*, *J* = 282.0 Hz, 1F); ESI-HRMS, *m*/*z*: Calcd for C_16_H_15_BrF_2_N_2_O [M + H]^+^, 353.0096, found: 353.0107.
*(±)-2-(3-Bromophenyl)-3-(2,2-difluoro-1-hydroxy)ethyl-imidazo[*1,2-*a]pyridine* (**3l**), white solid (59 mg, 84%); m.p. 186–188 °C; ^1^H-NMR (400 MHz, CDCl_3_:CD_3_OD = 7:1), *δ_H_*, ppm: 5.27–5.35 (*m*, 1H), 6.01–6.16 (*m*, 1H), 6.78–6.82 (*m*, 1H), 7.17–7.22 (*m*, 2H), 7.38–7.45 (*m*, 3H), 7.67 (*s*, 1H), 8.62 (*d*, *J* = 6.4 Hz, 1H); ^13^C-NMR (100 MHz, CDCl_3_:CD_3_OD = 7:1), *δ_C_*, ppm: 66.22 (*t*, *J* = 25.0 Hz), 112.6, 115.3 (*t*, *J* = 245.0 Hz), 115.9, 116.6, 122.5, 126.3, 127.4, 130.0, 131.3, 131.7, 135.2, 143.7, 145.6; ^19^F NMR (376 MHz, CDCl_3_:CD_3_OD = 7:1), *δ_F_*, ppm: −123.88 (*d*, *J* = 285.8 Hz, 1F), −124.66 (*d*, *J* = 285.8 Hz, 1F); ESI-HRMS, *m*/*z*: Calcd for C_15_H_12_BrF_2_N_2_O [M + H]^+^, 353.0096, found: 353.0107.
*(±)-2-(3,4-Dichlorophenyl)-3-(2,2-difluoro-1-hydroxy)ethyl-imidazo[*1,2-*a]pyridine* (**3m**), white solid (57 mg, 84%); m.p. 205–207 °C; ^1^H-NMR (400 MHz, CDCl_3_:CD_3_OD = 7:1), *δ_H_*, ppm: 5.26–5.34 (*m*, 1H), 6.18 (*td*, *J* = 55.6, 3.6 Hz, 1H), 6.83–6.87 (*m*, 1H), 7.25–7.30 (*m*, 1H), 7.38–7.53 (*m*, 3H), 7.68 (*s*, 1H), 8.63 (*d*, *J* = 6.4 Hz, 1H); ^13^C-NMR (100 MHz, CDCl_3_:CD_3_OD=7:1), *δ_C_*, ppm: 66.2 (*t*, *J* = 26.0 Hz), 112.7, 115.2 (*t*, *J* = 245.0 Hz), 116.0, 116.6, 126.5, 127.4, 128.0, 130.5, 132.5, 132.6, 133.2, 142.9, 145.6; ^19^F NMR (376 MHz, CDCl_3_:CD_3_OD = 7:1), *δ_F_*, ppm: −123.85 (*d*, *J* = 285.8 Hz, 1F), −124.66 (*d*, *J* = 285.8 Hz, 1F); ESI-HRMS, *m*/*z*: Calcd for C_15_H_11_Cl_2_F_2_N_2_O [M + H]^+^, 343.0211, found: 343.0213.
*(±)-3-(2,2-Difluoro-1-hydroxy)ethyl-2-(3,4-dimethoxyphenyl)-imidazo[*1,2-*a]pyridine* (**3n**), white solid (55 mg, 82%); m.p. 178–180 °C; ^1^H-NMR (400 MHz, CDCl_3_:CD_3_OD = 7:1), *δ_H_*, ppm: 3.86 (*s*, 3H), 3.90 (*s*, 3H), 5.35–5.42 (*m*, 1H), 6.19 (*td*, *J* = 55.6, 3.6 Hz, 1H), 6.78–6.86 (*m*, 2H), 7.04 (*d*, *J* = 8.0 Hz, 1H), 7.16 (*s*, 1H), 7.19–7.23 (*m*, 1H), 7.46 (*d*, *J* = 8.8 Hz, 1H), 8.61 (*d*, *J* = 6.8 Hz, 1H); ^13^C-NMR (100 MHz, CDCl_3_:CD_3_OD = 7:1), *δ_C_*, ppm: 55.7, 55.7, 66.3 (*t*, *J* = 25.0 Hz), 110.9, 112.1, 112.3, 115.1, 115.4 (*t*, *J* = 245.0 Hz), 116.3, 121.2, 125.8, 125.9, 127.2, 145.3, 145.4, 148.7, 149.0; ^19^F NMR (376 MHz, CDCl_3_:CD_3_OD = 7:1), *δ_F_*, ppm: −123.54 (*d*, *J* = 282.0 Hz, 1F), −124.53 (*d*, *J* = 282.0 Hz, 1F); ESI-HRMS, *m*/*z*: Calcd for C_17_H_17_F_2_N_2_O_3_ [M + H]^+^, 335.1202, found: 335.1195.
*(±)-3-(2,2-Difluoro-1-hydroxy)ethyl-2-(naphthalen-1-yl)-imidazo[*1,2-*a]pyridine* (**3o**), white solid (51 mg, 78%); m.p. 212–214 °C; ^1^H-NMR (400 MHz, CDCl_3_), *δ_H_*, ppm: 4.68–4.76 (*m*, 1H), 5.45 (*td*, *J* = 55.6, 2.8 Hz, 1H), 6.67–6.71 (*m*, 1H), 6.94–6.99 (*m*, 2H), 7.06–7.10 (*m*, 1H), 7.15–7.19 (*m*, 1H), 7.25–7.30 (*m*, 2H), 7.41 (*d*, *J* = 8.4 Hz, 1H), 7.74 (*d*, *J* = 8.0 Hz, 1H), 8.46 (*d*, *J* = 6.8 Hz, 1H); ^13^C-NMR (100 MHz, CDCl_3_), *δ_C_*, ppm: 66.0 (*t*, *J* = 25.0 Hz), 112.3, 115.2 (*t*, *J* = 245.0 Hz), 116.4, 117.2, 124.9, 125.6, 125.9, 125.9, 126.2, 127.3, 128.0, 128.3, 129.0, 130.2, 132.2, 133.4, 143.8, 145.3; ESI-HRMS, *m*/*z*: Calcd for C_19_H_15_F_2_N_2_O [M + H]^+^, 325.1147, found: 325.1154.
*(±)-3-(2,2-Difluoro-1-hydroxy)ethyl-2-(naphthalen-2-yl)-imidazo[*1,2-*a]pyridine* (**3p**), white solid (52 mg, 80%); m.p. 210–212 °C; ^1^H-NMR (400 MHz, CDCl_3_:CD_3_OD = 7:1), *δ_H_*, ppm: 5.38–5.42 (*m*, 1H), 6.14 (*td*, *J* = 55.6, 3.2 Hz, 1H), 6.57–6.61 (*m*, 1H), 6.96–7.01 (*m*, 1H), 7.33 (*d*, *J* = 8.8 Hz, 1H), 7.47–7.51 (*m*, 3H), 7.65 (*d*, *J* = 8.4 Hz, 1H), 7.73–7.77 (*m*, 2H), 7.85 (*s*, 1H), 8.53 (*d*, *J* = 6.8 Hz, 1H); ^13^C-NMR (100 MHz, CDCl_3_:CD_3_OD=7:1), *δ_C_*, ppm: 66.3 (*t*, *J* = 25.0 Hz), 112.2, 115.4 (*t*, *J* = 245.0 Hz), 115.8, 116.3, 125.8, 126.3, 126.3, 126.4, 127.2, 127.6, 127.8, 128.1, 128.3, 130.4, 132.9, 133.0, 145.2, 145.5; ^19^F NMR (376 MHz, CDCl_3_:CD_3_OD = 7:1), *δ_F_*, ppm: −123.52 (*d*, *J* = 285.8 Hz, 1F), −124.60 (*d*, *J* = 285.8 Hz, 1F); ESI-HRMS, *m*/*z*: Calcd for C_19_H_15_F_2_N_2_O [M + H]^+^, 325.1147, found: 325.1154.
*(±)-3-(2,2-Difluoro-1-hydroxy)ethyl-2-(pyridin-2-yl)-imidazo[*1,2-*a]pyridine* (**3q**), white solid (37 mg, 68%); m.p. 165–167 °C; ^1^H-NMR (400 MHz, CDCl_3_), *δ_H_*, ppm: 5.25–5.32 (*m*, 1H), 5.87 (*td*, *J* = 55.6, 5.6 Hz, 1H), 6.78–6.82 (*m*, 1H), 7.14–7.23 (*m*, 2H), 7.56 (*d*, *J* = 9.2 Hz, 1H), 7.78–7.82 (*m*, 1H), 8.03 (*d*, *J* = 6.8 Hz, 1H), 8.38 (*d*, *J* = 8.0 Hz, 1H), 8.45 (*d*, *J* = 4.0 Hz, 1H), 9.82 (*br*, 1H); ^13^C-NMR (100 MHz, CDCl_3_), *δ_C_*, ppm: 65.8 (*t*, *J* = 26.0 Hz), 113.3, 115.9 (*t*, *J* = 244.0 Hz), 118.0, 120.1, 123.0, 123.0, 124.0, 125.6, 138.4, 142.6, 145.1, 147.2, 152.6; ^19^F NMR (376 MHz, CDCl_3_), *δ_F_*, ppm: −123.20 (*d*, *J* = 282.0 Hz, 1F), −127.07 (*d*, *J* = 282.0 Hz, 1F); ESI-HRMS, *m*/*z*: Calcd for C_14_H_12_F_2_N_3_O [M + H]^+^, 276.0943, found: 276.0962.
*(±)-3-(2,2-Difluoro-1-hydroxy)ethyl-2-(thiophen-2-yl)-imidazo[*1,2-*a]pyridine* (**3r**), white solid (41 mg, 73%); m.p. 163–165 °C; ^1^H-NMR (400 MHz, CDCl_3_:CD_3_OD = 7:1), *δ_H_*, ppm: 5.46–5.54 (*m*, 1H), 6.10 (*td*, *J* = 55.6, 3.2 Hz, 1H), 6.69–6.73 (*m*, 1H), 6.96–6.99 (*m*, 1H), 7.11–7.16 (*m*, 1H), 7.19 (*d*, *J* = 3.6 Hz, 1H), 7.27 (*d*, *J* = 5.2 Hz, 1H), 7.40 (*d*, *J* = 9.2 Hz, 1H), 8.57 (*d*, *J* = 7.2 Hz, 1H); ^13^C-NMR (100 MHz, CDCl_3_:CD_3_OD = 7:1), *δ_C_*, ppm: 66.4 (*t*, *J* = 25.0 Hz), 112.4, 114.9, 115.4 (*t*, *J* = 246.0 Hz), 116.4, 126.2, 126.3, 126.7, 127.4, 127.6, 135.5, 139.1, 145.7; ^19^F NMR (376 MHz, CDCl_3_), *δ_F_*, ppm: −123.44 (*d*, *J* = 282.0 Hz, 1F), −127.37 (*d*, *J* = 282.0 Hz, 1F); ESI-HRMS, *m*/*z*: Calcd for C_13_H_11_F_2_N_2_OS [M + H]^+^, 281.0555, found: 281.0566.
*(±)-3-(2,2-Difluoro-1-hydroxy)ethyl-6-methyl-2-phenyl-imidazo[*1,2-*a]pyridine* (**3aa**), white solid (54 mg, 93%); m.p. 212–214 °C; ^1^H-NMR (400 MHz, CDCl_3_:CD_3_OD = 7:1), *δ_H_*, ppm: 2.30 (*s*, 3H), 5.28–5.36 (*m*, 1H), 6.13 (*td*, *J* = 55.6, 4.0 Hz, 1H), 7.00 (*d*, *J* = 9.2 Hz, 1H), 7.26–7.31 (*m*, 4H), 7.38–7.41 (*m*, 2H), 8.35 (*s*, 1H); ^13^C-NMR (100 MHz, CDCl_3_:CD_3_OD = 7:1), *δ_C_*, ppm: 18.4, 66.3 (*t*, *J* = 25.0 Hz), 115.1, 115.2 (*t*, *J* = 245.0 Hz), 115.8, 122.0, 124.8, 128.0, 128.4, 128.7, 129.0, 133.1, 144.5, 145.2; ^19^F NMR (376 MHz, CDCl_3_:CD_3_OD = 7:1), *δ_F_*, ppm: −123.27 (*d*, *J* = 282.0 Hz, 1F), −124.93 (*d*, *J* = 282.0 Hz, 1F); ESI-HRMS, *m*/*z*: Calcd for C_16_H_15_F_2_N_2_O [M + H]^+^, 289.1147, found: 289.1136.
*(±)-3-(2,2-Difluoro-1-hydroxy)ethyl-7-methyl-2-phenyl-imidazo[*1,2-*a]pyridine* (**3ab**), white solid (55 mg, 95%); m.p. 186–188 °C; ^1^H-NMR (400 MHz, CDCl_3_), *δ_H_*, ppm: 2.32 (*s*, 3H), 5.26–5.34 (*m*, 1H), 6.18 (*td*, *J* = 55.6, 3.6 Hz, 1H), 7.55 (*d*, *J* = 8.8 Hz, 1H), 7.17–7.29 (*m*, 6H), 8.43 (*d*, *J* = 9.2 Hz, 1H); ^13^C-NMR (100 MHz, CDCl_3_), *δ_C_*, ppm: 21.3, 66.4 (*t*, *J* = 25.0 Hz), 114.8, 115.1, 115.3 (*t*, *J* = 245.0 Hz), 126.5, 128.1, 128.4, 128.6, 132.7, 137.3, 144.8, 145.7; ^19^F NMR (376 MHz, CDCl_3_), *δ_F_*, ppm: −123.73 (*d*, *J* = 282.0 Hz, 1F), −124.81 (*d*, *J* = 282.0 Hz, 1F); ESI-HRMS, *m*/*z*: Calcd for C_16_H_15_F_2_N_2_O [M + H]^+^, 289.1147, found: 289.1136.
*(±)-3-(2,2-Difluoro-1-hydroxy)ethyl-8-methyl-2-phenyl-imidazo[*1,2-*a]pyridine* (**3ac**), white solid (49 mg, 84%); m.p. 184–186 °C; ^1^H-NMR (400 MHz, CDCl_3_:CD_3_OD = 7:1), *δ_H_*, ppm: 2.53 (*s*, 3H), 5.21–5.28 (*m*, 1H), 6.08 (*td*, *J* = 55.6, 1.6 Hz, 1H), 6.70–6.74 (*m*, 1H), 7.03 (*d*, *J* = 6.0 Hz, 1H), 7.28–7.32 (*m*, 3H), 7.45–7.48 (*m*, 2H), 8.48 (*d*, *J* = 6.8 Hz, 1H); ^13^C-NMR (100 MHz, CDCl_3_:CD_3_OD = 7:1), *δ_C_*, ppm: 16.9, 66.2 (*t*, *J* = 25.0 Hz), 112.4, 115.2 (*t*, *J* = 245.0 Hz), 115.9, 124.8, 124.9, 126.7, 128.1, 128.3, 129.1, 133.3, 145.2, 145.9; ^19^F NMR (376 MHz, CDCl_3_:CD_3_OD = 7:1), *δ_F_*, ppm: −123.51 (*d*, *J* = 282.0 Hz, 1F), −124.57 (*d*, *J* = 282.0 Hz, 1F); ESI-HRMS, *m*/*z*: Calcd for C_16_H_15_F_2_N_2_O [M + H]^+^, 289.1147, found: 289.1136.
*(±)-3-(2,2-Difluoro-1-hydroxy)ethyl-7-methoxy-2-phenyl-imidazo[*1,2-*a]pyridine* (**3ad**), white solid (52 mg, 85%); m.p. 201–203 °C; ^1^H-NMR (400 MHz, CDCl_3_:CD_3_OD = 7:1), *δ_H_*, ppm: 3.86 (*s*, 3H), 5.27–5.32 (*m*, 1H), 5.99–6.28 (*m*, 1H), 6.78–6.81 (*m*, 1H), 7.31–7.42 (*m*, 3H), 7.49–7.59 (*m*, 2H), 8.46 (*d*, *J* = 4.8 Hz, 1H); ^13^C-NMR (100 MHz, CDCl_3_:CD_3_OD = 7:1), *δ_C_*, ppm: 55.5, 66.2 (*t*, *J* = 25.0 Hz), 93.7, 107.3, 114.3, 115.4 (*t*, *J* = 245.0 Hz), 127.8, 128.1, 128.4, 128.7, 133.3, 145.0, 147.3, 158.8; ^19^F NMR (376 MHz, CDCl_3_:CD_3_OD = 7:1), *δ_F_*, ppm: −123.98 (*d*, *J* = 282.0 Hz, 1F), −125.18 (*d*, *J* = 282.0 Hz, 1F); ESI-HRMS, *m*/*z*: Calcd for C_16_H_15_F_2_N_2_O_2_ [M + H]^+^, 305.1096, found: 305.1081.
*(±)-6-Chloro-3-(2,2-difluoro-1-hydroxy)ethyl-2-phenyl-imidazo[*1,2-*a]pyridine* (**3ae**), white solid (57 mg, 92%); m.p. 215–217 °C; ^1^H-NMR (400 MHz, CDCl_3_:CD_3_OD = 7:1), *δ_H_*, ppm: 5.30–5.38 (*m*, 1H), 6.16 (*td*, *J* = 55.6, 3.2 Hz, 1H), 7.18–7.22 (*m*, 1H), 7.36–7.42 (*m*, 3H), 7.46 (*d*, *J* = 9.2 Hz, 1H), 7.51–7.54 (*m*, 2H), 8.72 (*s*, 1H); ^13^C-NMR (100 MHz, CDCl_3_:CD_3_OD = 7:1), *δ_C_*, ppm: 66.3 (*t*, *J* = 25.0 Hz), 115.3 (*t*, *J* = 245.0 Hz), 116.2, 116.9, 120.5, 125.3, 127.2, 128.5, 128.6, 128.8, 132.8, 144.0, 146.3; ^19^F NMR (376 MHz, CDCl_3_:CD_3_OD = 7:1), *δ_F_*, ppm: −123.99 (*d*, *J* = 282.0 Hz, 1F), −124.97 (*d*, *J* = 282.0 Hz, 1F); ESI-HRMS, *m*/*z*: Calcd for C_15_H_12_ClF_2_N_2_O [M + H]^+^, 309.0601, found: 309.0616.
*(±)-7-Chloro-3-(2,2-difluoro-1-hydroxy)ethyl-7-chloro-2-phenyl-imidazo[*1,2-*a]pyridine* (**3af**), white solid (56mg, 91%); m.p. 219–221 °C; ^1^H-NMR (400 MHz, CDCl_3_:CD_3_OD = 7:1), *δ_H_*, ppm: 5.34–5.41 (*m*, 1H), 6.16 (*t*, *J* = 55.6 Hz, 1H), 6.83 (*d*, *J* = 7.2 Hz, 1H), 7.33–7.47 (*m*, 3H); 7.59 (*d*, *J* = 4.4 Hz, 2H), 8.62 (*d*, *J* = 7.2 Hz, 1H); ^13^C-NMR (100 MHz, DMSO-*d*6), *δ_C_*, ppm: 65.8 (*t*, *J* = 24.0 Hz), 113.8, 116.0, 116.3 (*t*, *J* = 243.0 Hz), 116.8, 128.8, 128.9, 129.0, 129.2, 131.3, 133.8, 145.2, 146.0; ^19^F NMR (376 MHz, CDCl_3_:CD_3_OD = 7:1), *δ_F_*, ppm: −124.13 (*d*, *J* = 285.8 Hz, 1F), −125.03 (*d*, *d*, *J* = 285.8 Hz, 1F); ESI-HRMS, *m*/*z*: Calcd for C_15_H_12_ClF_2_N_2_O [M + H]^+^, 309.0601, found: 309.0616.
*(±)-8-Chloro-3-(2,2-difluoro-1-hydroxy)ethyl-8-chloro-2-phenyl-imidazo[*1,2-*a]pyridine* (**3ag**), white solid (55 mg, 89%); m.p. 233–235 °C; ^1^H-NMR (400 MHz, CDCl_3_:CD_3_OD = 7:1), *δ_H_*, ppm: 5.32–5.39 (*m*, 1H), 6.08 (*td*, *J* = 55.6, 3.2 Hz, 1H), 6.78–6.82 (*m*, 1H), 7.34–7.45 (*m*, 4H), 7.62 (*d*, *J* = 6.4 Hz, 2H), 8.64 (*d*, *J* = 7.2 Hz, 1H); ^13^C-NMR (100 MHz, DMSO-d6), *δ_C_*, ppm: 65.9 (*t*, *J* = 25.0 Hz), 112.5, 116.2 (*t*, *J* = 243.0 Hz), 118.4, 121.8, 125.2, 127.2, 128.9, 129.2, 129.2, 133.8, 142.5, 145.6; ^19^F NMR (376 MHz, CDCl_3_:CD_3_OD = 7:1), *δ_F_*, ppm: −124.06 (*d*, *J* = 282.0 Hz, 1F), −125.11 (*d*, *J* = 282.0 Hz, 1F); ESI-HRMS, *m*/*z*: Calcd for C_15_H_12_ClF_2_N_2_O [M + H]^+^, 309.0601, found: 309.0616.
*(±)-8-Bromo-3-(2,2-difluoro-1-hydroxy)ethyl-2-phenyl-imidazo[*1,2-*a]pyridine* (**3ah**), white solid (58 mg, 83%); m.p. 259–261 °C; ^1^H-NMR (400 MHz, CDCl_3_:CD_3_OD = 7:1), *δ_H_*, ppm: 5.32–5.38 (*m*, 1H), 6.01–6.30 (*m*, 1H), 6.73–6.77 (*m*, 1H), 7.41–7.46 (*m*, 3H), 7.55 (*d*, *J* = 6.8 Hz, 1H), 7.64 (*d*, *J* = 6.8 Hz, 2H), 8.69 (*d*, *J* = 6.0 Hz, 1H); ^13^C-NMR (100 MHz, DMSO-d6), *δ_C_*, ppm: 66.0 (*t*, *J* = 25.0 Hz), 110.6, 113.0, 116.2 (*t*, *J* = 243.0 Hz), 118.4, 127.6, 128.6, 128.8, 129.1, 129.2, 133.8, 143.1, 145.6; ^19^F NMR (376 MHz, CDCl_3_:CD_3_OD = 7:1), *δ_F_*, ppm: −123.98 (*d*, *J* = 282.0 Hz, 1F), −125.16 (*d*, *J* = 282.0 Hz, 1F); ESI-HRMS, *m*/*z*: Calcd for C_15_H_12_BrF_2_N_2_O [M + H]^+^, 353.0096, found: 353.0107.
*(±)-3-(2,2-Difluoro-1-hydroxy)ethyl-2-phenyl-6-(trifluoromethyl)-imidazo[*1,2-*a]pyridine* (**3ai**), white solid (61 mg, 89%); m.p. 193–195 °C; ^1^H-NMR (400 MHz, CDCl_3_:CD_3_OD = 7:1), *δ_H_*, ppm: 5.35–5.43 (*m*, 1H), 6.17 (*td*, *J* = 55.6, 2.8 Hz, 1H), 7.33–7.39 (*m*, 4H), 7.48–7.51 (*m*, 2H), 7.59 (*d*, *J* = 9.8 Hz, 1H), 9.02 (*s*, 1H); ^13^C-NMR (100 MHz, CDCl_3_:CD_3_OD = 7:1), *δ_C_*, ppm: 66.1 (*t*, *J* = 25.0 Hz), 115.5 (*t*, *J* = 245.0 Hz), 116.5 (*q*, *J* = 34.0 Hz), 116.9, 117.3, 121.6–121.8 (*m*), 123.5 (*q*, *J* = 270.0 Hz), 126.8 (*q*, *J* = 5.0 Hz), 128.6, 128.7, 128.7, 132.4, 145.4, 147.1; ^19^F NMR (376 MHz, CDCl_3_:CD_3_OD = 7:1), *δ_F_*, ppm: −62.05, −123.79 (*d*, *J* = 282.0 Hz, 1F), −125.09 (*d*, *J* = 282.0 Hz, 1F); ESI-HRMS, *m*/*z*: Calcd for C_16_H_12_F_5_N_2_O [M + H]^+^, 343.0864, found: 343.0857.
*(±)-3-(2,2-Difluoro-1-hydroxy)ethyl-2-phenyl-7-(trifluoromethyl)-imidazo[*1,2-*a]pyridine* (**3aj**), white solid (60 mg, 87%); m.p. 206–208 °C; ^1^H-NMR (400 MHz, CD_3_OD), *δ_H_*, ppm: 5.37–5.45 (*m*, 1H), 6.36 (*td*, *J* = 55.6, 3.6 Hz, 1H), 7.18 (*dd*, *J* = 6.4, 3.2 Hz, 1H), 7.44–7.54 (*m*, 3H), 7.66–7.69 (*m*, 2H); 7.93 (*s*, 1H), 8.96 (*d*, *J* = 7.2 Hz, 1H); ^13^C-NMR (CD_3_OD), *δ_C_*, ppm: 66.1 (*t*, *J* = 25.0 Hz), 107.6–107.7 (*m*), 113.8 (*q*, *J* = 4.0 Hz), 115.8 (*t*, *J* = 245.0 Hz), 118.0 (*t*, *J* = 3.0 Hz), 123.4 (*q*, *J* = 272.0 Hz), 127.5 (*q*, *J* = 34.0 Hz), 128.4, 128.6, 128.8, 129.0, 132.7, 143.7, 147.3; ^19^F NMR (376 MHz, CD_3_OD), *δ_F_*, ppm: −65.30, −125.65 (*d*, *J* = 285.8 Hz, 1F), −126.62 (*d*, *J* = 285.8 Hz, 1F); ESI-HRMS, *m*/*z*: Calcd for C_16_H_12_F_5_N_2_O [M + H]^+^, 343.0864, found: 343.0857.
*(±)-6-Bromo-3-(2,2-difluoro-1-hydroxy)ethyl-7-methyl-2-phenyl-6-(trifluoro-methyl)-imidazo[*1,2-*a]pyridine* (**3ak**), white solid (66 mg, 90%); m.p. 237–239 °C; ^1^H-NMR (400 MHz, DMSO-d6), *δ_H_*, ppm: 5.32–5.39 (*m*, 1H), 6.59 (*t*, *J* = 53.2 Hz, 1H), 6.86–6.88 (*m*, 1H), 7.40–7.51 (*m*, 3H), 7.54–7.70 (*m*, 3H), 8.85 (*s*, 1H); ^13^C-NMR (100 MHz, DMSO-d6), *δ_C_*, ppm: 22.4, 65.8 (*t*, *J* = 24.0 Hz), 110.5, 116.0, 116.3 (*t*, *J* = 243.0 Hz), 116.6, 127.4, 127.7, 129.0, 129.1, 134.0, 135.7, 144.7, 145.6; ^19^F NMR (376 MHz, DMSO-d6), *δ_F_*, ppm: −123.85 (*d*, *J* = 282.0 Hz, 1F), −124.80 (*d*, *J* = 282.0 Hz, 1F); ESI-HRMS, *m*/*z*: Calcd for C_16_H_14_BrF_2_N_2_O [M + H]^+^, 367.0252, found: 367.0244.
*3-(2,2-Difluoroacetyl)-imidazo[*1,2-*a]pyridine* (**4a**), white solid (15 mg, 77%); m.p. 102–104 °C; ^1^H-NMR (400 MHz, CDCl_3_), *δ_H_*, ppm: 6.26 (*t*, *J* = 53.6 Hz, 1H), 7.21–7.25 (*m*, 1H), 7.63–7.68 (*m*, 1H), 7.87 (*d*, *J* = 9.2 Hz, 1H), 8.63 (*s*, 1H), 9.62 (*d*, *J* = 6.8 Hz, 1H); ^13^C-NMR (100 MHz, CDCl_3_), *δ_C_*, ppm: 111.1 (*t*, *J* = 252.0 Hz), 116.2, 118.1, 119.9, 129.0, 131.0, 146.0 (*t*, *J* = 6.0 Hz), 149.7, 177.0 (*t*, *J* = 26.0 Hz); ^19^F NMR (376 MHz, CDCl_3_), *δ*, ppm: −121.40; ESI-HRMS, *m*/*z*: Calcd for C_9_H_7_F_2_N_2_O [M + H]^+^, 197.0521, found: 197.0539.
*3-(2,2-Difluoroacetyl)-2-phenyl-imidazo[*1,2-*a]pyridine* (**4b**), white solid (22 mg, 79%); m.p. 121–123 °C; ^1^H-NMR (400 MHz, CDCl_3_), *δ_H_*, ppm: 5.91 (*t*, *J* = 53.2 Hz, 1H), 7.23–7.26 (*m*, 1H), 7.52–7.58 (*m*, 3H), 7.62–7.65 (*m*, 2H), 7.66–7.72 (*m*, 1H), 7.85 (*d*, *J* = 8.8 Hz, 1H), 9.78 (*d*, *J* = 6.8 Hz, 1H); ^13^C-NMR (100 MHz, CDCl_3_), *δ_C_*, ppm: 106.0 (*t*, *J* = 244.0 Hz), 116.2, 117.7, 119.0, 128.8, 129.4, 129.7, 130.2, 131.6, 133.7, 148.5, 157.5, 177.2 (*t*, *J* = 25.0 Hz); ^19^F NMR (376 MHz, CDCl_3_), *δ_F_*, ppm: −124.48; ESI-HRMS, *m*/*z*: Calcd for C_15_H_11_F_2_N_2_O [M + H]^+^, 273.0834, found: 273.0830.

## 4. Conclusions

In conclusion, we have developed a facile and efficient method for the synthesis of C3-difluoromethyl carbinol-containing imidazo[1,2-*a*]pyridines via HFIP-promoted direct C(sp^2^)-H hydroxydifluoromethylation. A small library of difluoromethylated carbinols were prepared at room temperature in good to high yields by the practical green method. This HFIP-promoted strategy exhibited some definite benefits, such as being transition metal- and oxidant-free and having wide substrate generality, excellent functional group tolerance and mild reaction conditions. In addition, gram-scale and synthetic transformation experiments have also been demonstrated. Therefore, this simple and green synthesis strategy might be attractive for the further design and rapid synthesis of potentially bioactive fluorinated heterocyclic derivatives with multifunctional groups.

## Figures and Tables

**Table 1:** Optimization of reaction conditions ^[a]^.

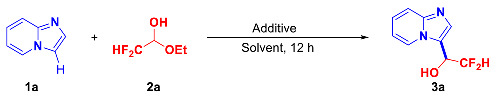			
Entry	Additive (Equiv.)	Solvent	Yield (%) ^[b]^
1	TFA (10%)	DCM	trace
2	TsOH (10%)	DCM	trace
3	BF_3_·OEt_2_	DCM	trace
4	Y(OTf)_3_ (10%)	DCM	trace
5	Sc(OTf)_3_ (10%)	DCM	trace
6	HFIP (10%)	DCM	trace
7	HFIP (1.0)	DCM	50
8	HFIP (2.0)	DCM	73
9		HFIP	97

^[a]^ Reaction conditions: **1a** (0.2 mmol), **2a** (0.4 mmol, 2.0 equiv.), solvent (1.0 mL), r.t., 12 h. ^[b]^ Isolated yield.

**Table 2:** Substrate scope of various 2-aryl imidazo[1,2-*a*]pyridines ^[a, b]^.

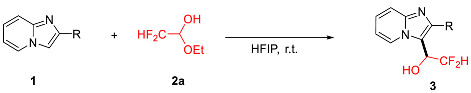
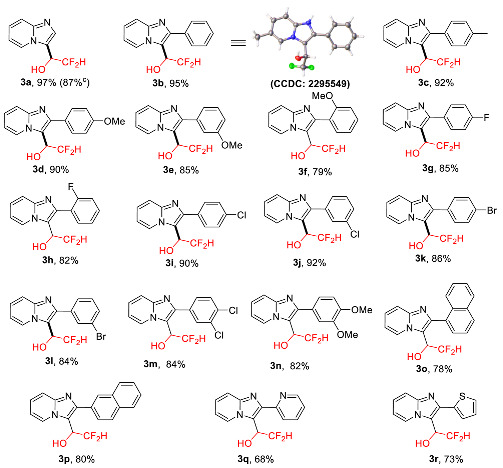

^[a]^ Reaction conditions: **1** (0.2 mmol), **2a** (0.4 mmol, 2.0 equiv.), HFIP (1.0 mL), r.t., 12 h. ^[b]^ Isolated yield. ^[c]^ Reaction performed on a 5 mmol scale.

**Table 3:** Substrate scope of various substituted 2-phenyl imidazo[1,2-*a*]pyridines ^[a, b]^.

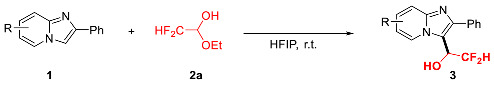
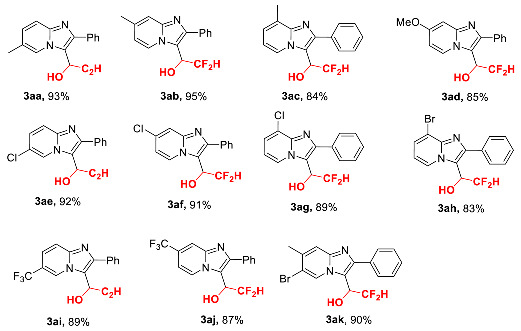

^[a]^ Reaction conditions: **1** (0.2 mmol), **2a** (0.4 mmol, 2.0 equiv.), HFIP (1.0 mL), r.t., 12 h. ^[b]^ Isolated yield.

## Data Availability

All data supporting the findings of this study are available within the paper and within its Supplementary Materials published online.

## Supplementary Materials

The following supporting information can be downloaded at: https://www.mdpi.com/article/10.3390/molecules28227522/s1, which contains details on the crystallographic information parameters (Table S1), experimental procedure for compounds **1b**–**1c**, experimental procedure for compounds **1z**–**1ac**, ^1^H, ^13^C and ^19^F NMR spectra for all compounds **3a**–**3ak** and **4a**–**4b**. References [24,37] are cited in the supplementary materials.
