# Supplementary material for: 1,1,1,3,3,3-Hexafluoro-2-Propanol-Promoted Friedel–Crafts Reaction: Metal-Free Synthesis of C3-Difluoromethyl Carbinol-Containing Imidazo[1,2-a]pyridines at Room Temperature
Source: Molecules. 2023 Nov 10;28(22):7522. doi: 10.3390/molecules28227522 (PMC10672982; doi:10.3390/molecules28227522)
Supplement: Supplementary file 1 [file molecules-28-07522-s001.zip › molecules-2686987-supplementary.pdf]

Supplementary Materials

1,1,1,3,3,3-Hexafluoro-2-Propanol-Promoted Friedel–Crafts Reaction: Metal-Free Synthesis of C3-Difluoromethyl Carbinol-Containing Imidazo[1,2-a]pyridines at Room Temperature

Juanjuan Gao [1], Zhaowen Liu [1], Xiaohua Guo [1], Longhui Wu [1], Zhixi Chen [1,*] and Kai Yang [1,*]

[1] College of Pharmacy, Gannan Medical University, Ganzhou, Jiangxi 341000, P. R. China; gaoya0758@163.com(J.G.); xhguo20041@163.com(X.G.); 13970997583@163.com(L.W.)

* Correspondence: czxb22@gmu.edu.cn (Z.C.); kai_yangyang@126.com (K.Y.); Fax: (+86)-0797-8169782.

Table of Contents

General Information...[2]

Experimental Procedure for Compounds **1b-1c**...[3]

Experimental Procedure for Compounds **1z-1ac**...[4]

Experimental Procedure for Compounds **3a-3ak and 4a-4b**...[5]

Data of Single-crystal X-ray Analysis (**Figure S1**, **Table S1**)...[6]

^{1}H, ^{13}C NMR and ^{19}F NMR Spectra for All Compounds **3a-3ak and 4a-4b**...[7-52]

References...[53]

General Information

Melting point (m.p.) was performed on a Büchi Melting Point B-545 instrument without correcting. ^{1}H, ^{13}C and ^{19}F NMR spectra were collected on a BRUKER DRX-400 spectrometer in $CDCl_3$ using tetramethylsilane (TMS) as an internal standard.

High-resolution mass spectra (HRMS) were obtained with a LCMS-IT-TOF mass spectrometer. Single-crystal X-ray analysis was obtained using Bruker APEX2 Smart CCD. TLC was performed by using commercially prepared 100-400 mesh silica gel plates (GF254) and visualization was detected at 254 or 365 nm.

All reagents and solvents were purchased from commercial sources and used without further purification, 2-substituted imidazo[1,2-a]pyridines **1** (except for **1a**) were synthesized from 2-bromoacetophenones (or acetophenones) and various 2-aminopyridines (see the following for details) [1,2].

Experimental Procedure for Compounds 1b-1y

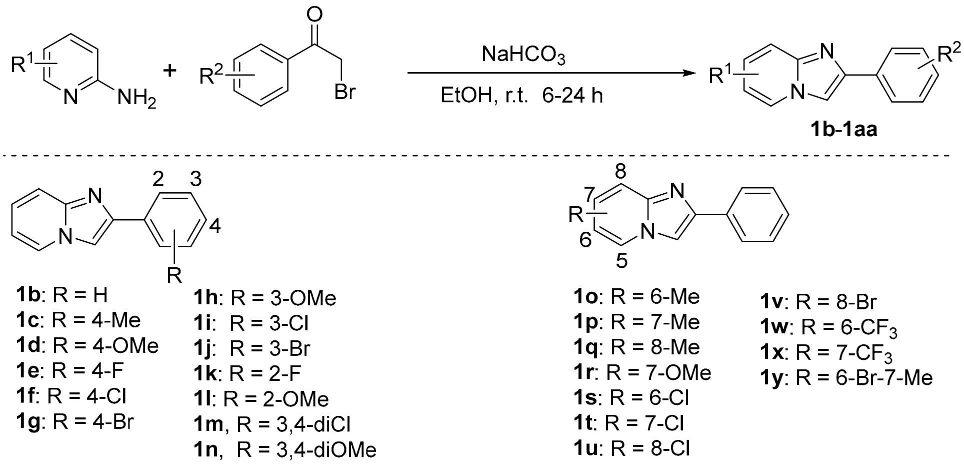

According to the reported procedure[1], compounds **1b-1y** were synthesized. To an ethanol solution containing 2-bromoacetophenones (20 mmol, 1.0 equiv.) and 2-aminopyridines (22 mmol, 1.1 equiv.) was added $NaHCO_3$ (24 mmol, 1.2 equiv.). The reaction mixture was stirred at room temperature for 6-24 hours. After completion of the reaction, the resulting mixture was diluted with water (15 mL) and extract with ether (3 × 20 mL). The combined organic layer was washed with brine (25 mL), dried with anhydrous $MgSO_4$, concentrated under vacuum to give the crude product, which was purified by silica gel column with petroleum ether/EtOAc as the eluent to give the analytical pure 2-arylimidazo[1,2-a]pyridine **1b-1y** in 50-90% yields.

Experimental Procedure for Compounds 1z-1ac

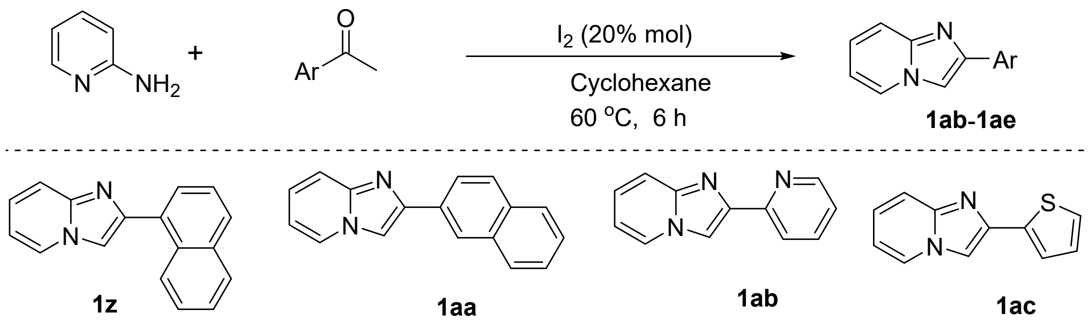

According to the reported procedure[2], compounds **1ab-1ae** were synthesized. A mixture of appropriate acetophenones (10 mmol), 2-amino pyridines (12 mmol), in cyclohexane (20 mL) was sealed, placed into an oil bath and heated at 60 °C for 6 h. After monitoring the end of the reaction on TLC, the resulting mixture was cooled to room temperature and washed with ethyl acetate. The above solution was evaporated under vacuum, and the residue was purified by silica gel column with petroleum ether/ethyl acetate as the eluent to give the analytically pure product 2-substituted imidazo[1,2-a]pyridines **1z-1ac** in 45-72% yield.

Experimental Procedure for Compounds 3a-3ak

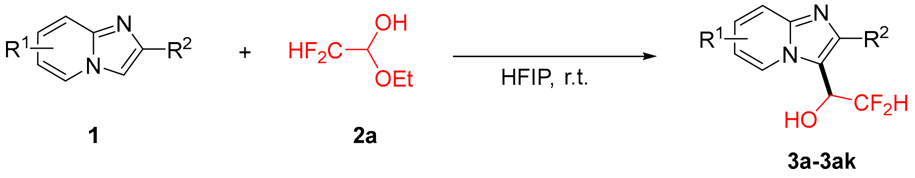

The mixture of imidazo[1,2-a]pyridine **1** (0.2 mmol, 1.0 equiv.), difluoroacetaldehyde ethyl hemiacetal **2a** (0.30 mmol, 1.5 equiv.) in 1,1,1,3,3,3-hexafluoro-2-propanol (1.0 mL) was stirred at room temperature 12 h. After the completion of the reaction, the reaction mixture was quenched with H_2O (15 mL) and extracted with ethyl acetate (3 × 15 mL). Then, the organic layer was dried over anhydrous Na_2SO_4. After filtration and evaporation of the solvents under reduced pressure, the crude product was purified by column chromatography on silica gel to afford the desired product **3**.

Experimental Procedure for Compounds 4a and 4b

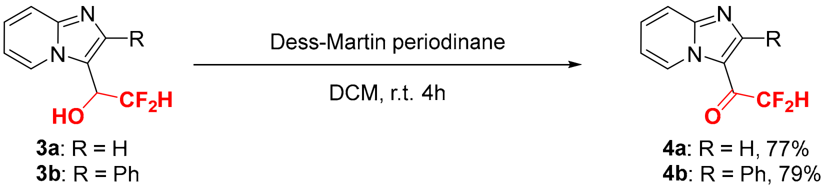

Compounds **3a** (0.1 mmol, 1.0 equiv.) or compounds **3b** (0.1 mmol, 1.0 equiv.), Dess-Martin periodinane (0.37 mmol, 3.7 equiv.) in DCM (1.0mL), white were stirred in a ground glass test tube at room temperature for 4 h. After monitoring the end of the reaction on TLC, the residue were purified by column chromatography on silica gel to give the pure products **4a** and **4b**.

Data of Single-crystal X-ray Analysis

Table S1. Crystal data and structure refinement for **3b**.

Compound	**3b**
Empirical formula	$C_{16}H_{15}F_2N_2O$
Formula weight	289.30
Crystal system	Orthorhombic
Space group	P212121
a (Å)	7.5039(5)
b (Å)	11.1588(8)
c (Å)	16.7454(12)
α (°)	90.00
β (°)	90.00
γ (°)	90.00
Volume(Å3)	1402.17(17)
Z	4
Dcalc (g cm-3)	1.370
F (000)	604.0
Reflections collected	4460
Independent reflections	2945
Rint	0.0119
Goodness-of-fit on F2	1.062
R1, wR2 [I >=2σ (I)]	0.0520, 0.1267
R1, wR2[all data]	0.0682, 0.1374

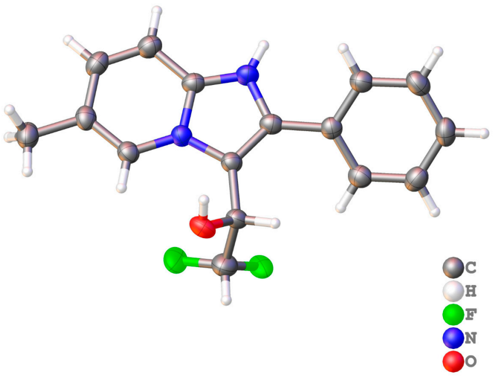

Figure S1. The molecular structure of **3b**.

NMR Spectra for All Compounds 3a-3ak and 4a-4b

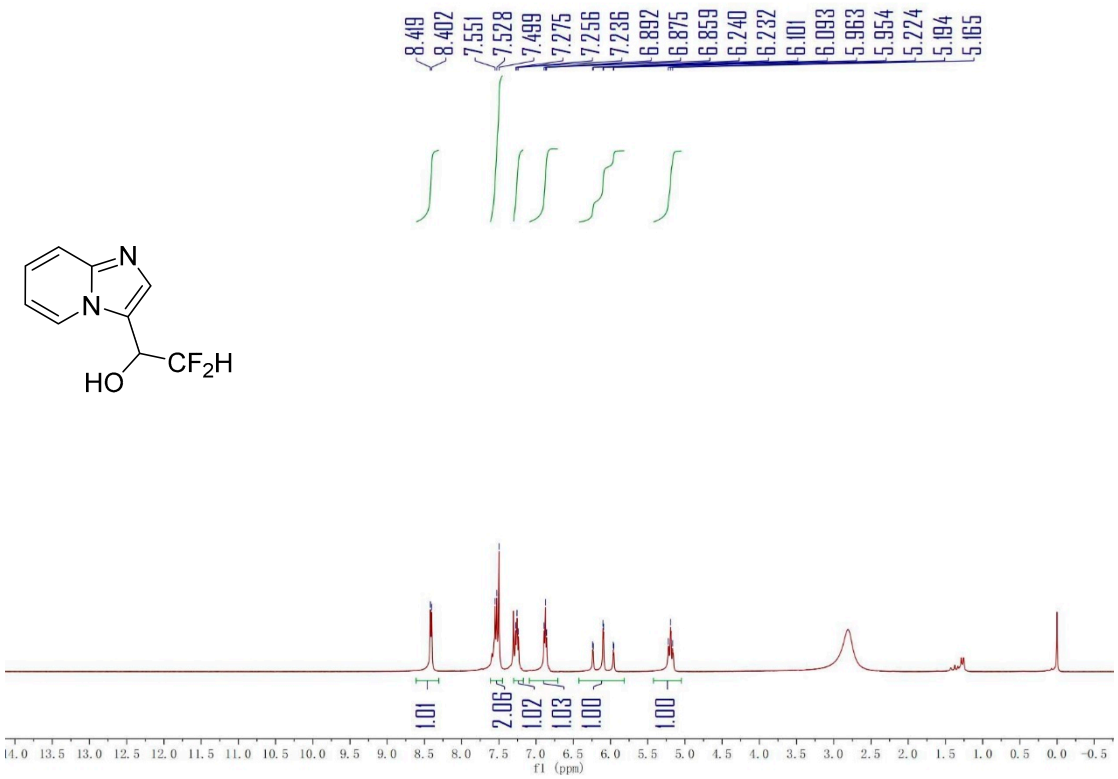

^{1}H NMR spectrum of compound **3a**

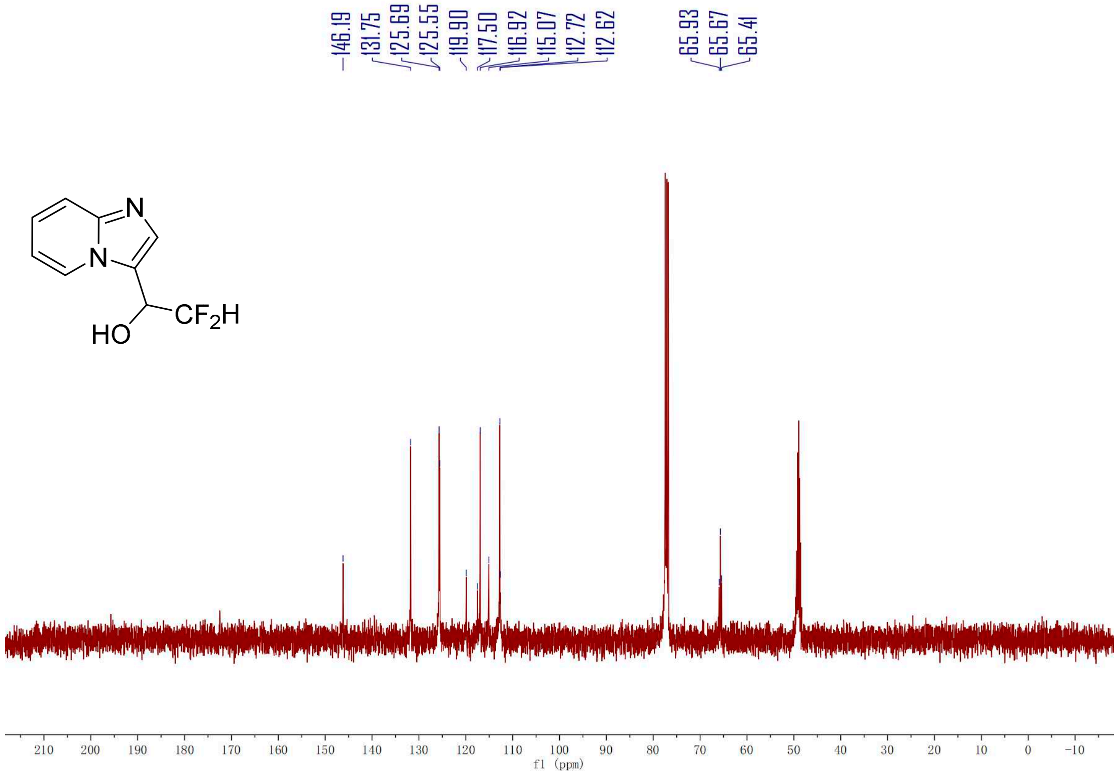

^{13}C NMR spectrum of compound **3a**

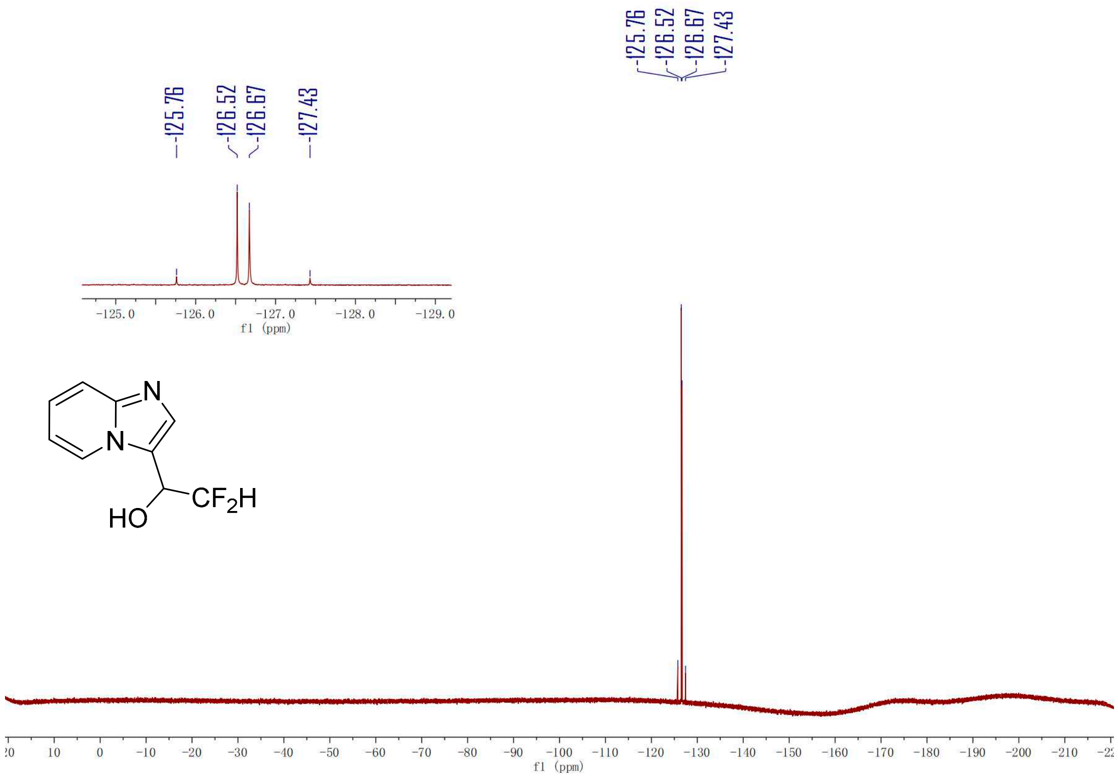

^{19}F NMR spectrum of compound **3a**.

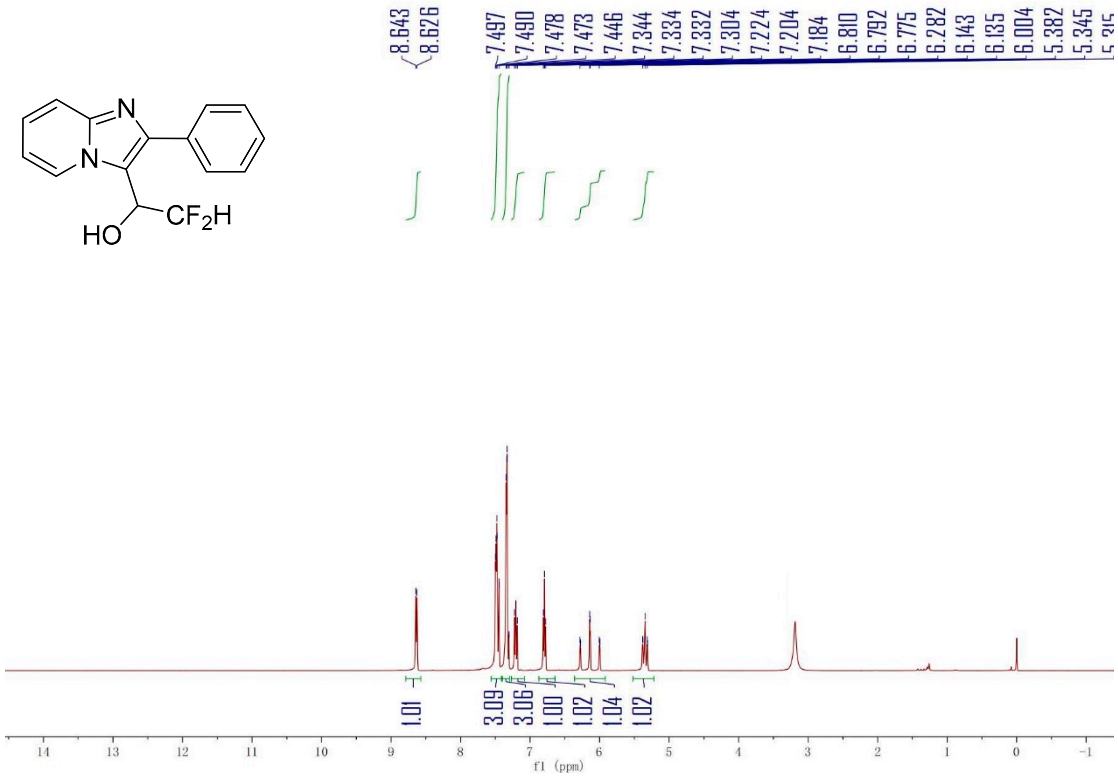

^{1}H NMR spectrum of compound **3b**.

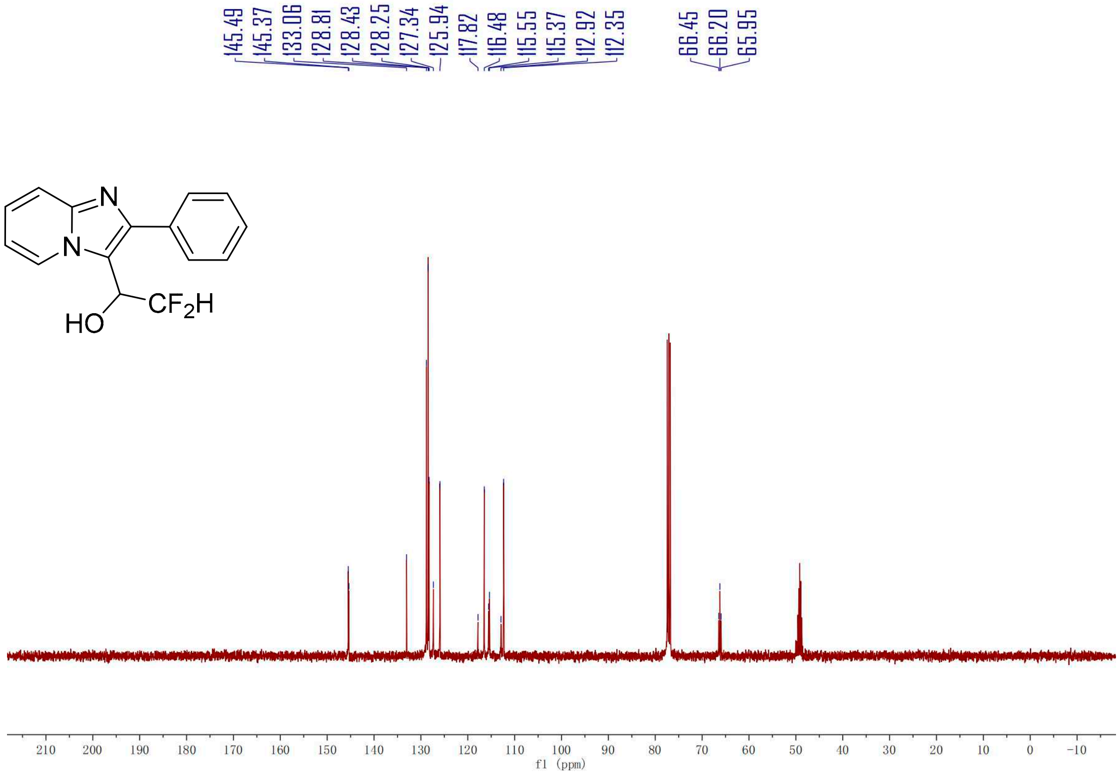

^{13}C NMR spectrum of compound **3b**.

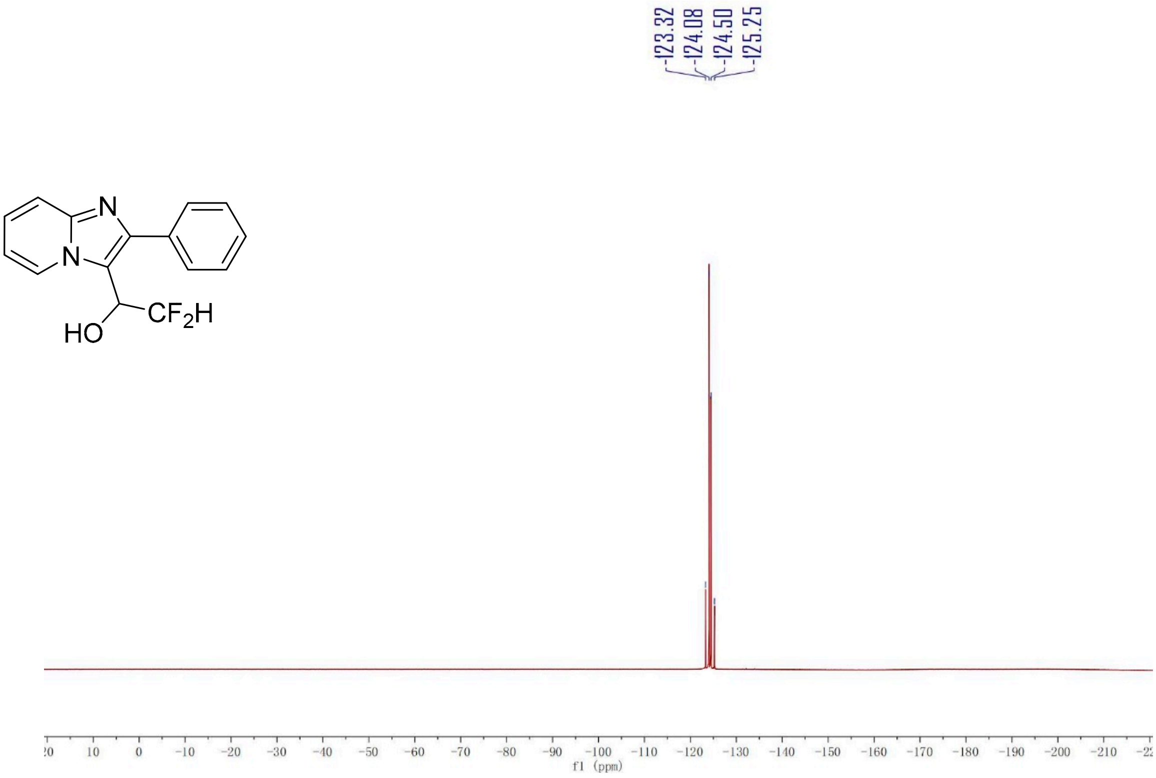

^{19}F NMR spectrum of compound **3b**.

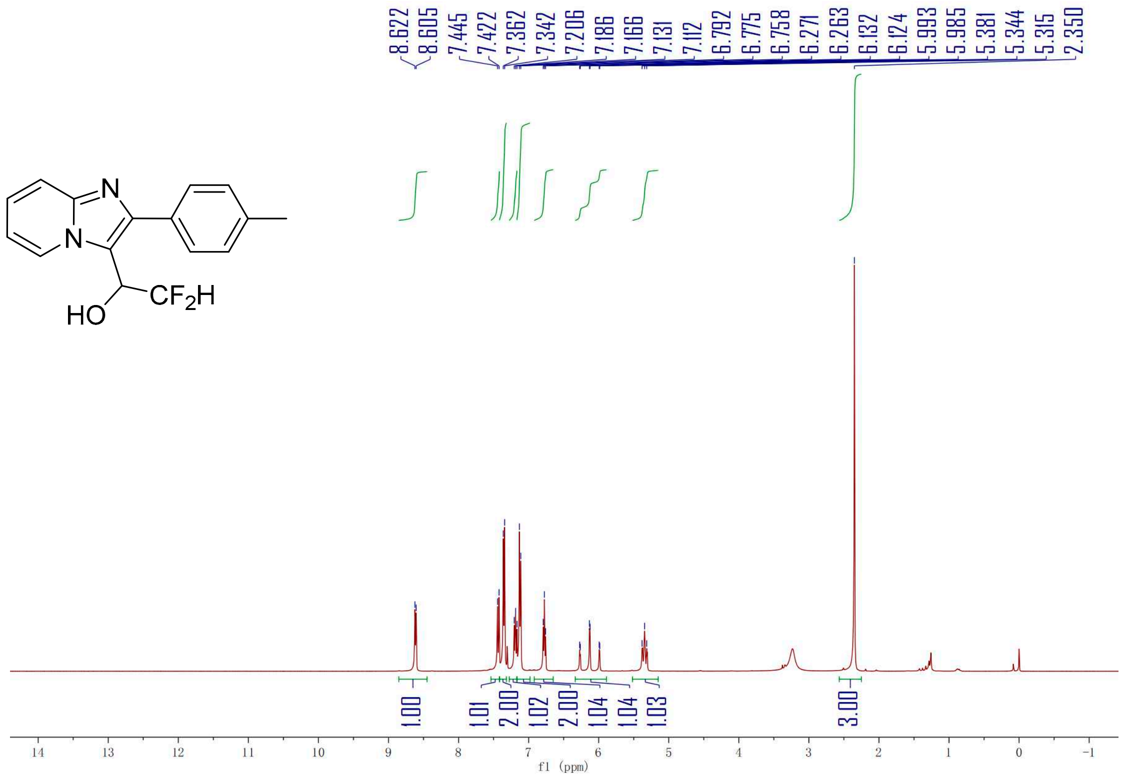

^{1}H NMR spectrum of compound **3c**.

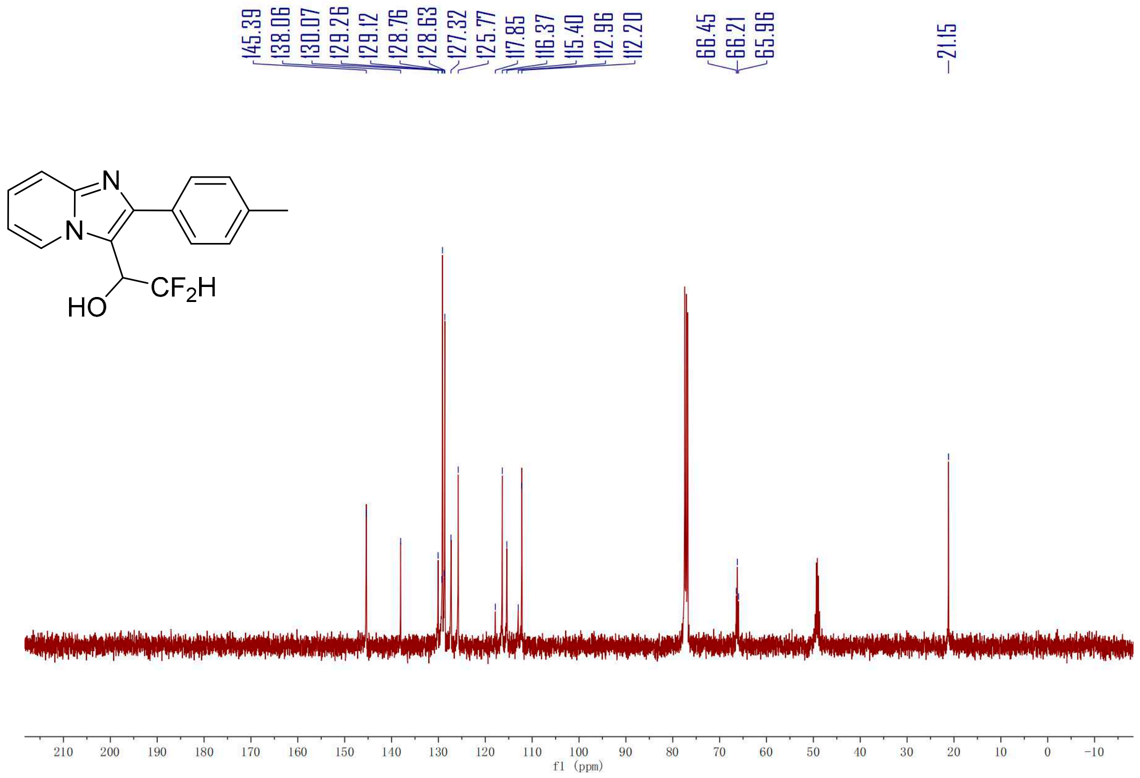

^{13}C NMR spectrum of compound **3c**.

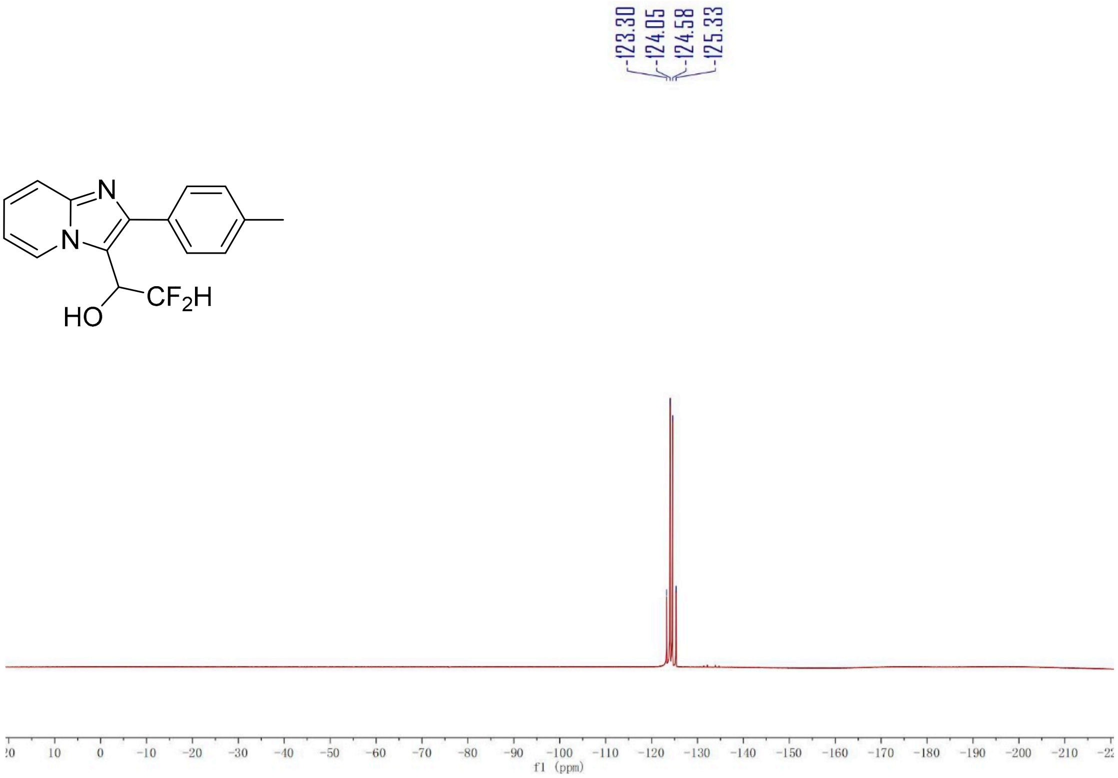

^{19}F NMR spectrum of compound **3c**.

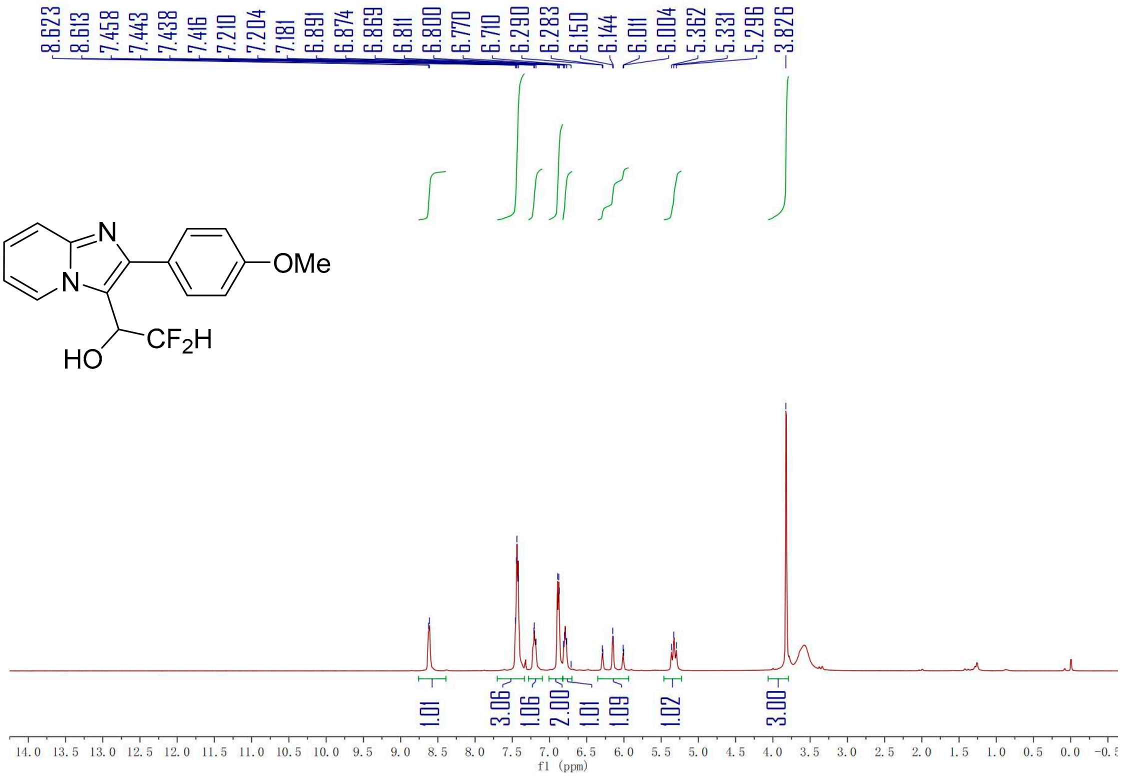

^{1}H NMR spectrum of compound **3d**.

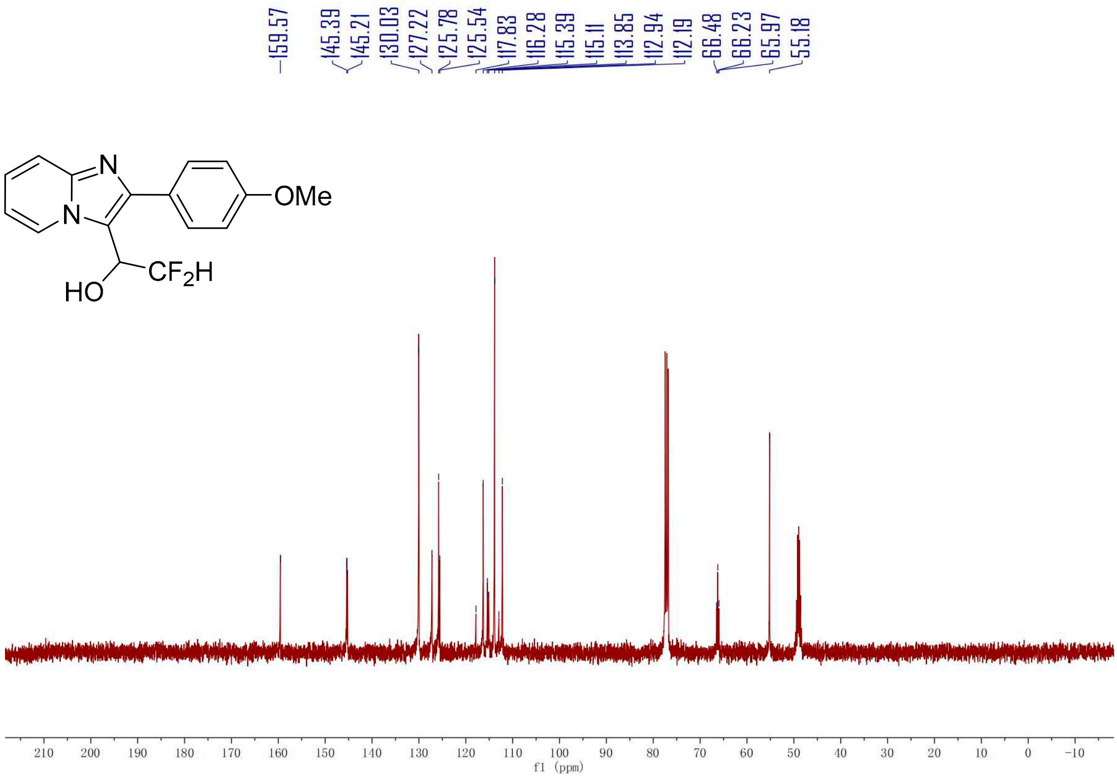

^{13}C NMR spectrum of compound **3d**.

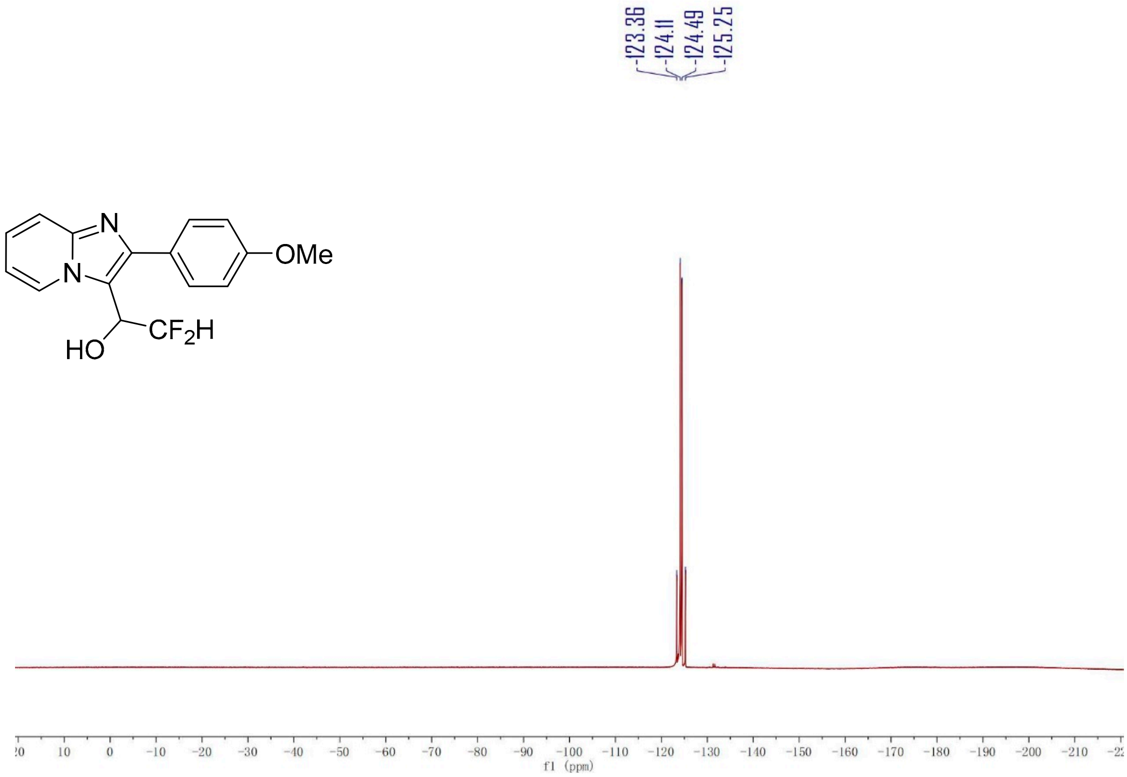

^{19}F NMR spectrum of compound **3d**.

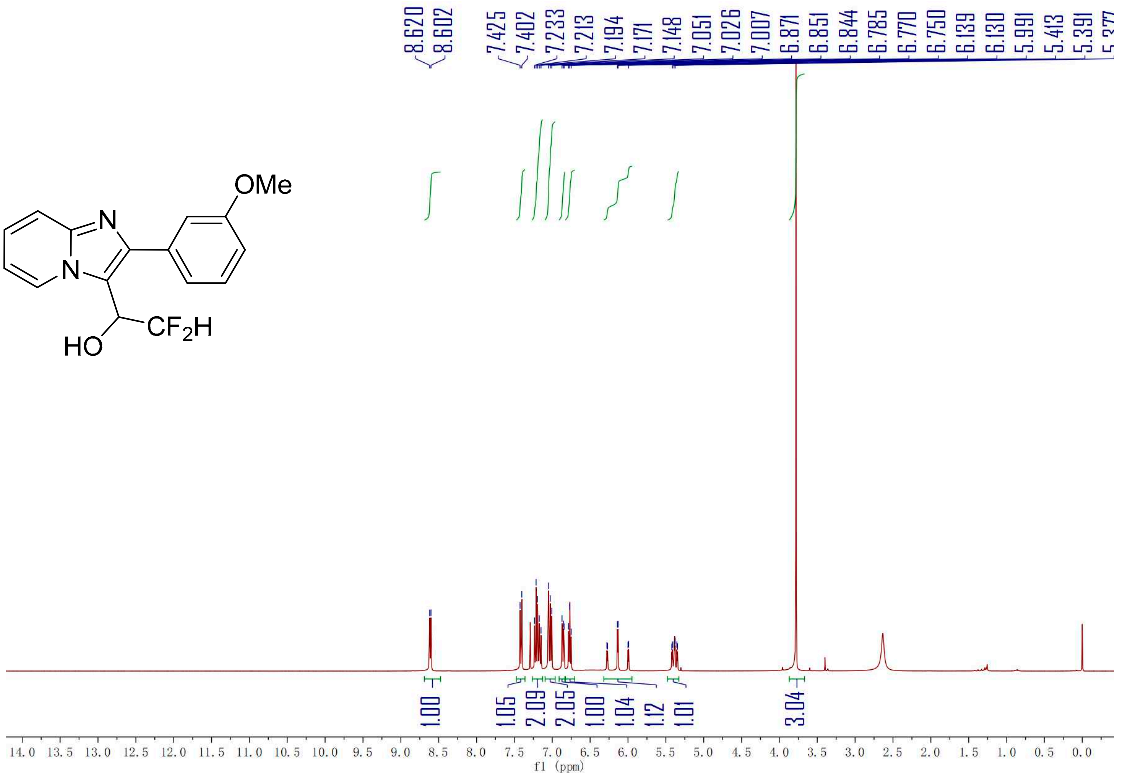

^{1}H NMR spectrum of compound **3e**.

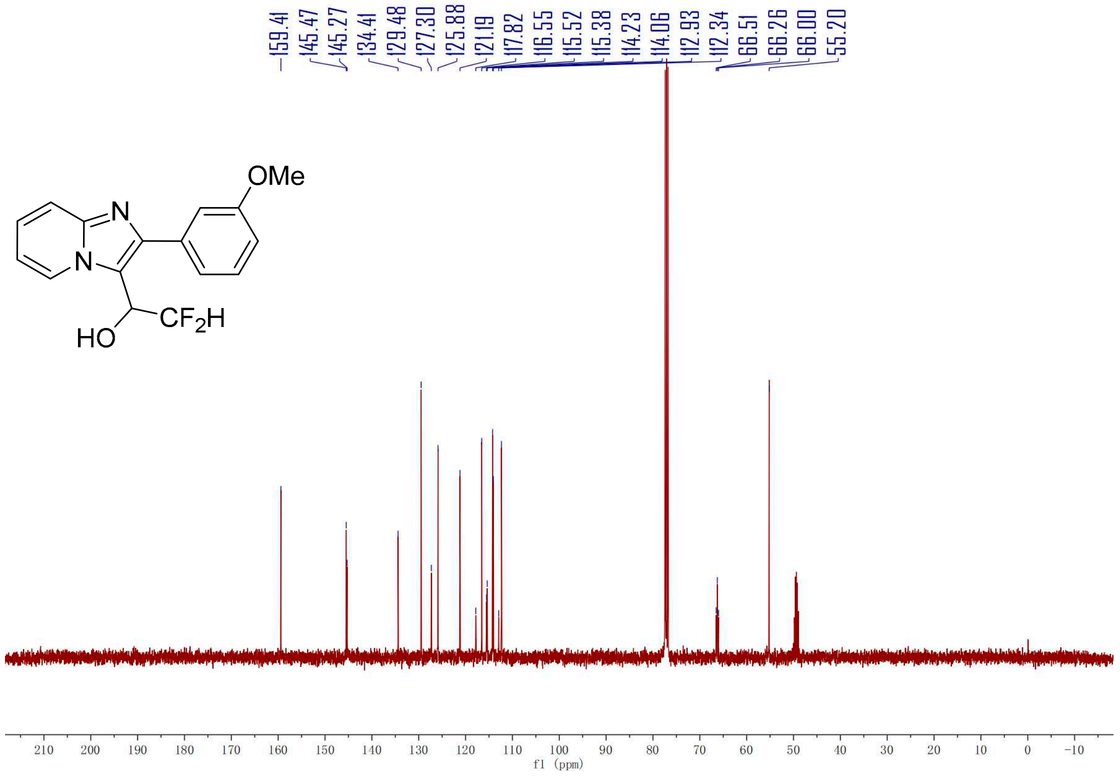

^{13}C NMR spectrum of compound **3e**.

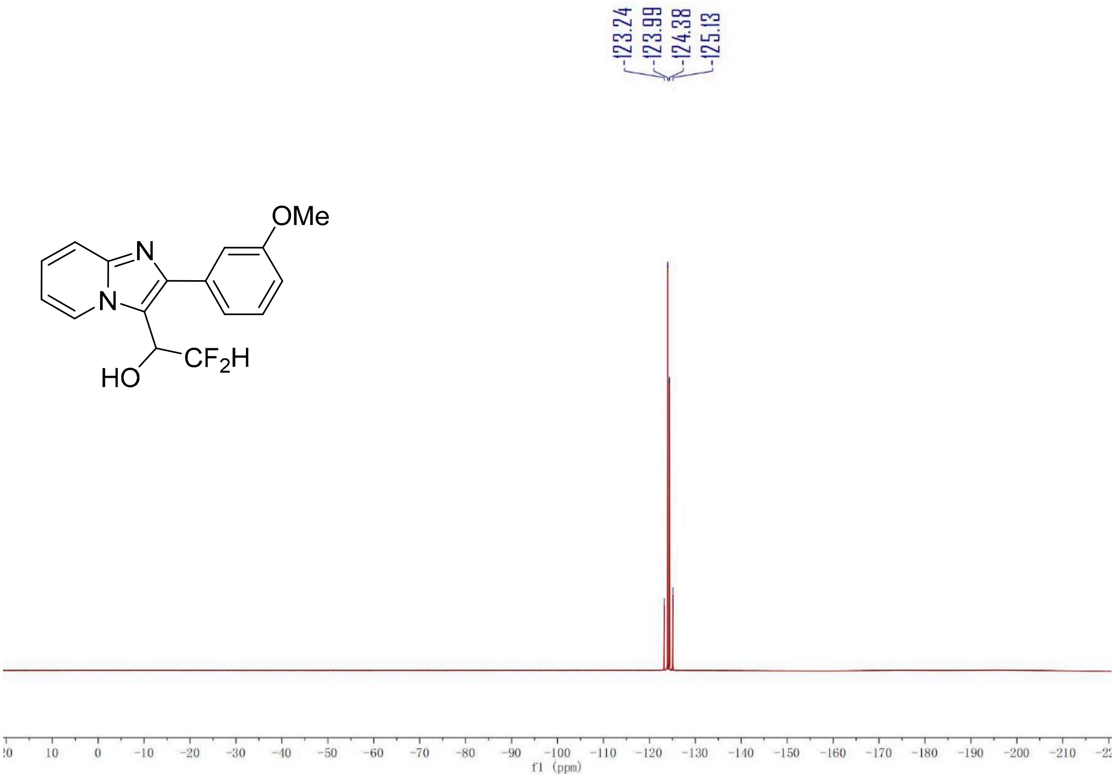

^{19}F NMR spectrum of compound **3e**.

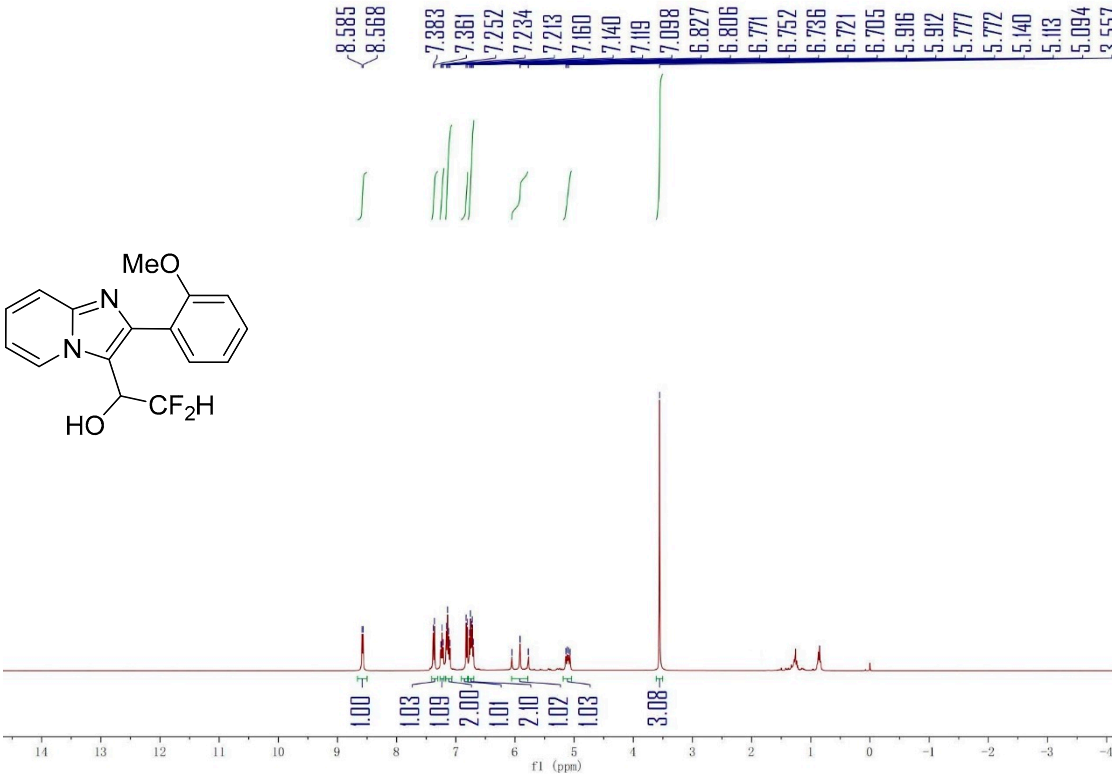

^{1}H NMR spectrum of compound **3f**.

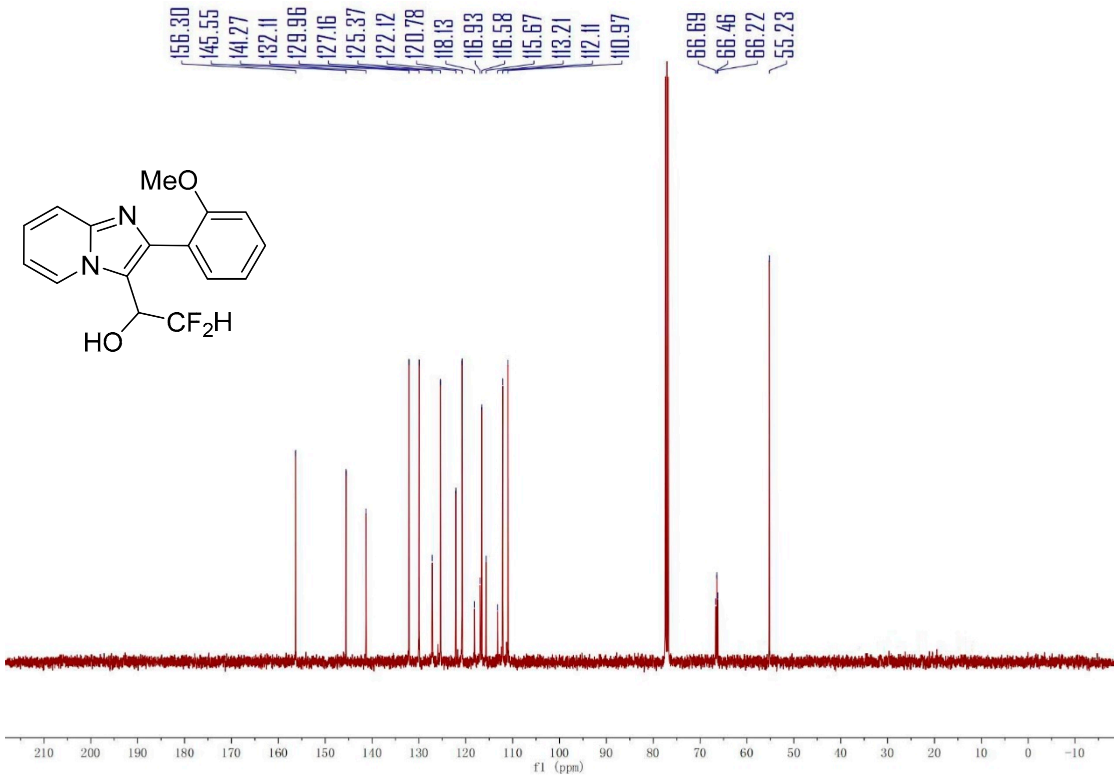

^{13}C NMR spectrum of compound **3f**.

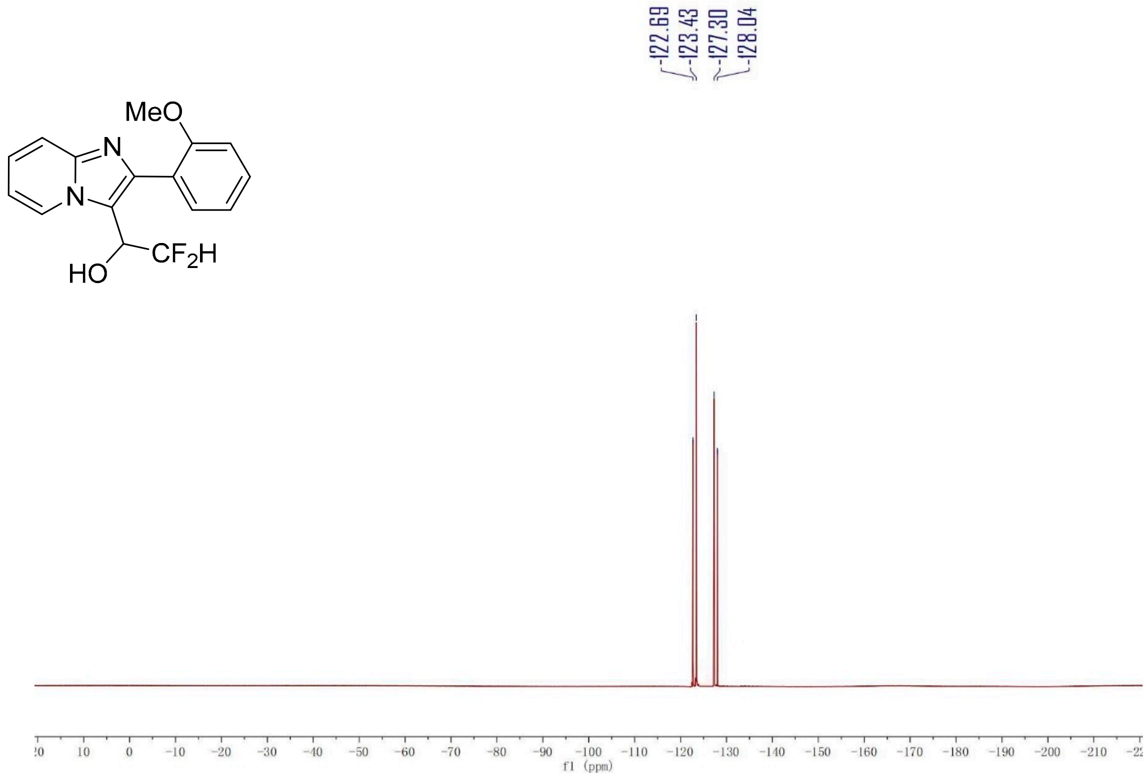

^{19}F NMR spectrum of compound **3f**.

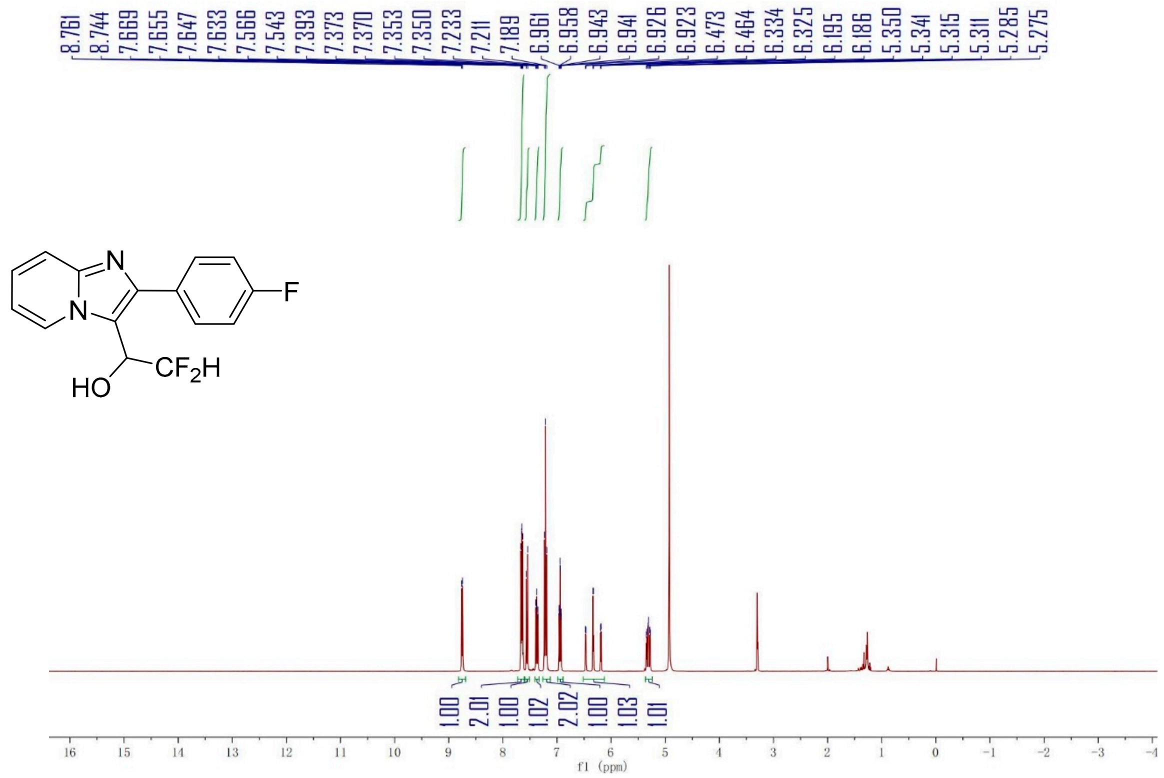

^{1}H NMR spectrum of compound **3g**.

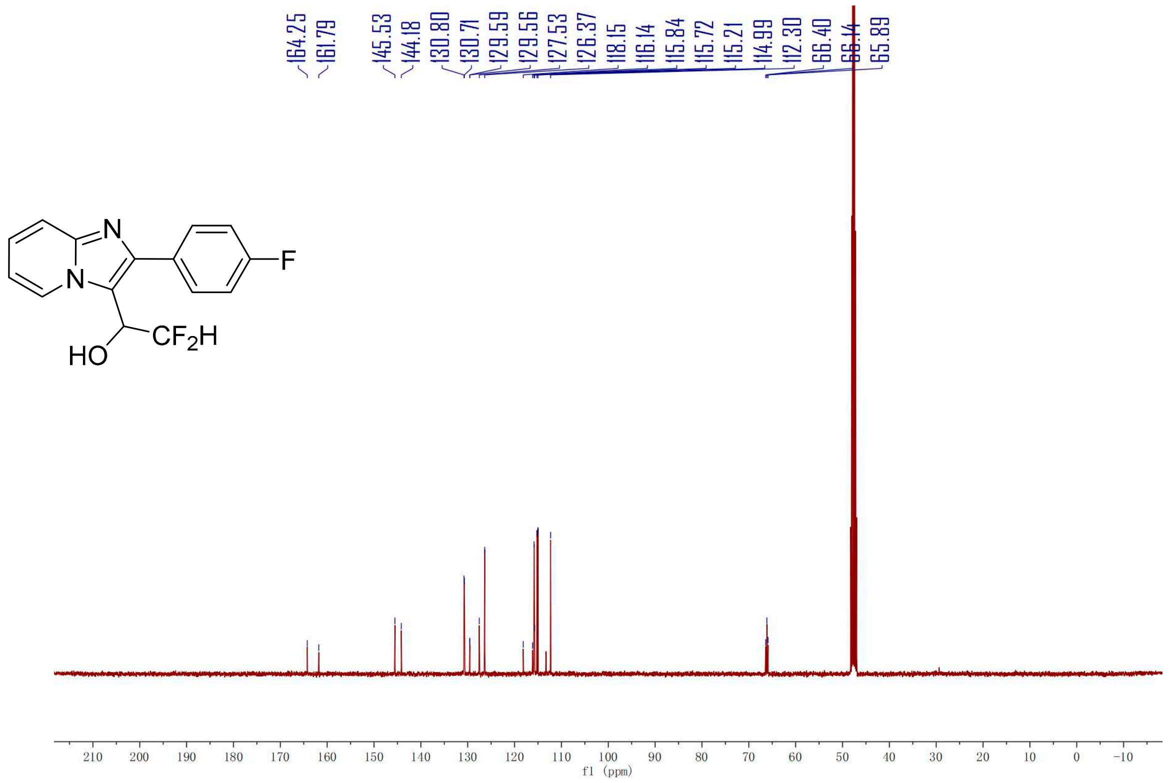

^{13}C NMR spectrum of compound **3g**.

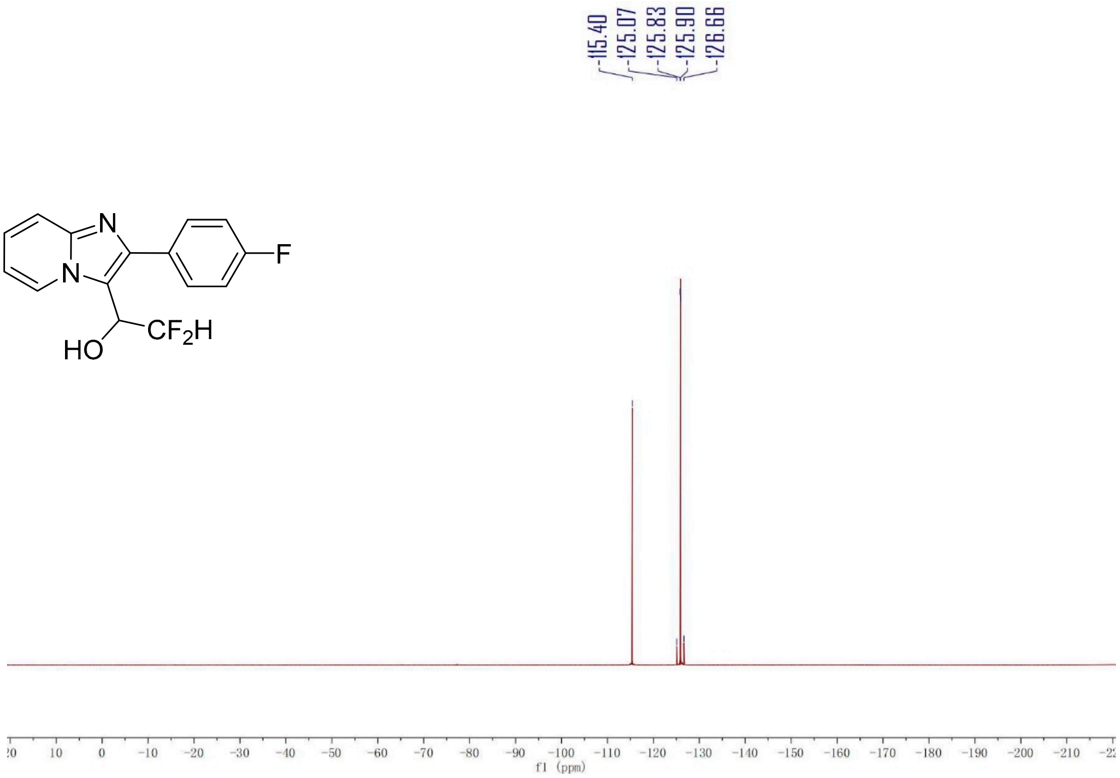

^{19}F NMR spectrum of compound **3g**.

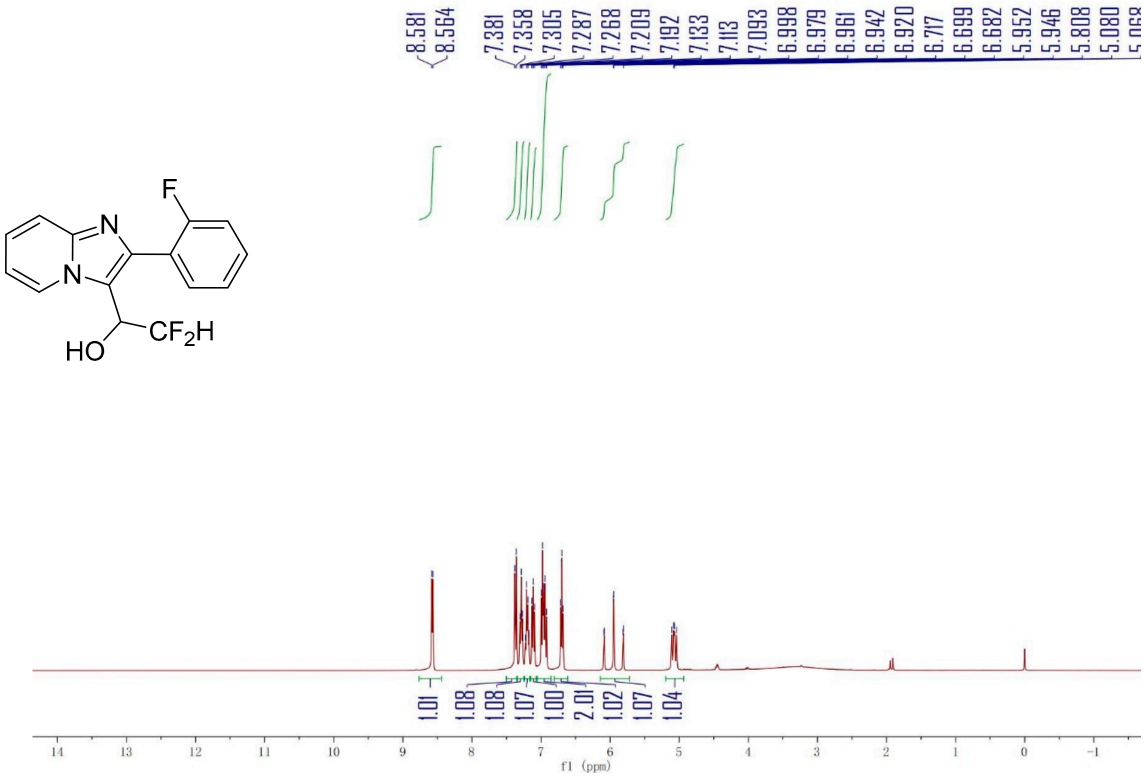

^{1}H NMR spectrum of compound **3h**.

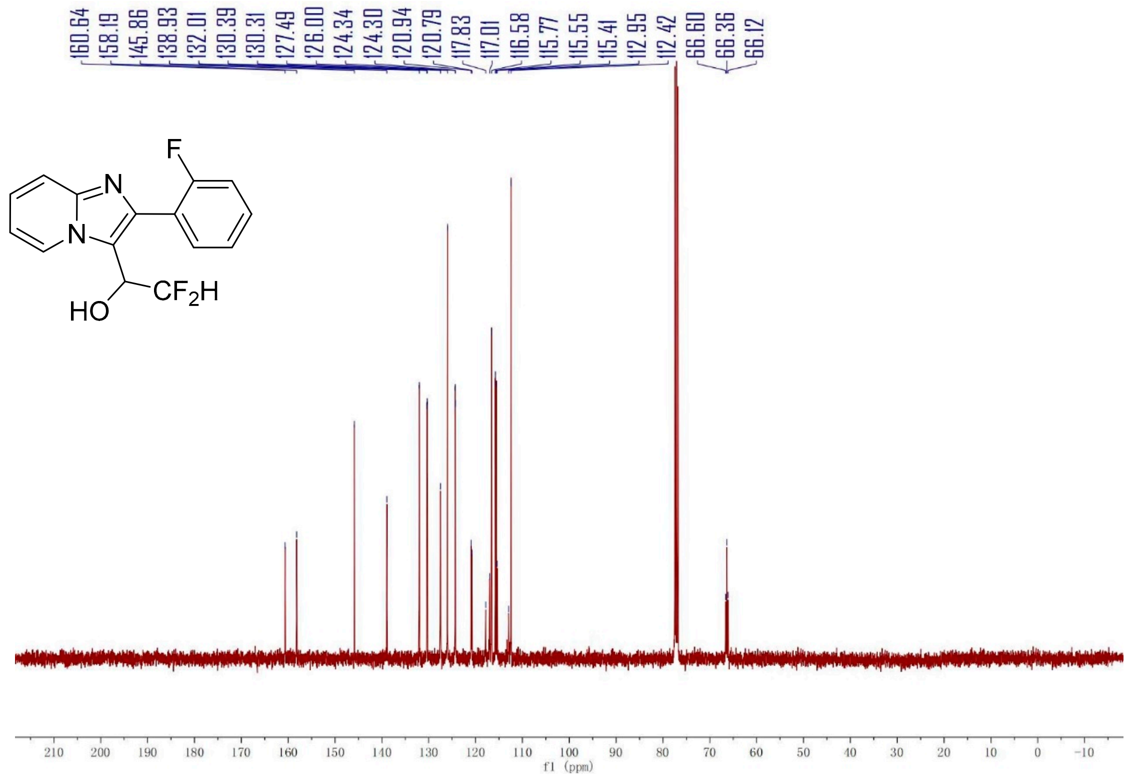

^{13}C NMR spectrum of compound **3h**.

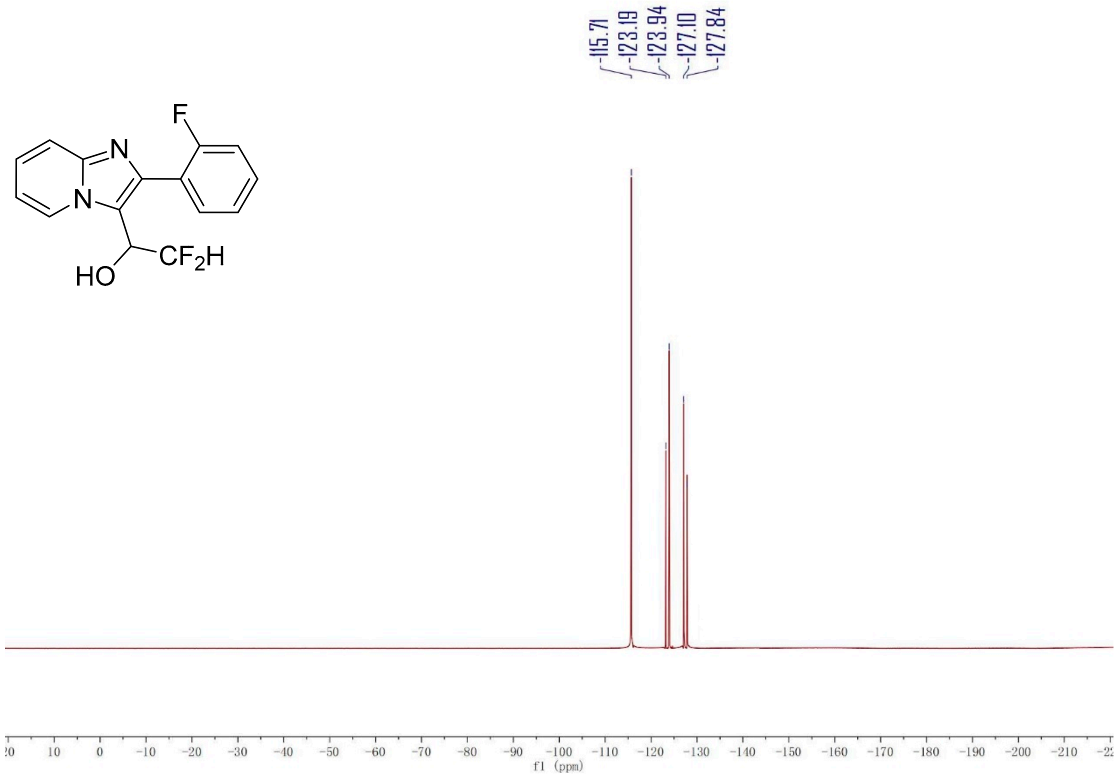

^{19}F NMR spectrum of compound **3h**.

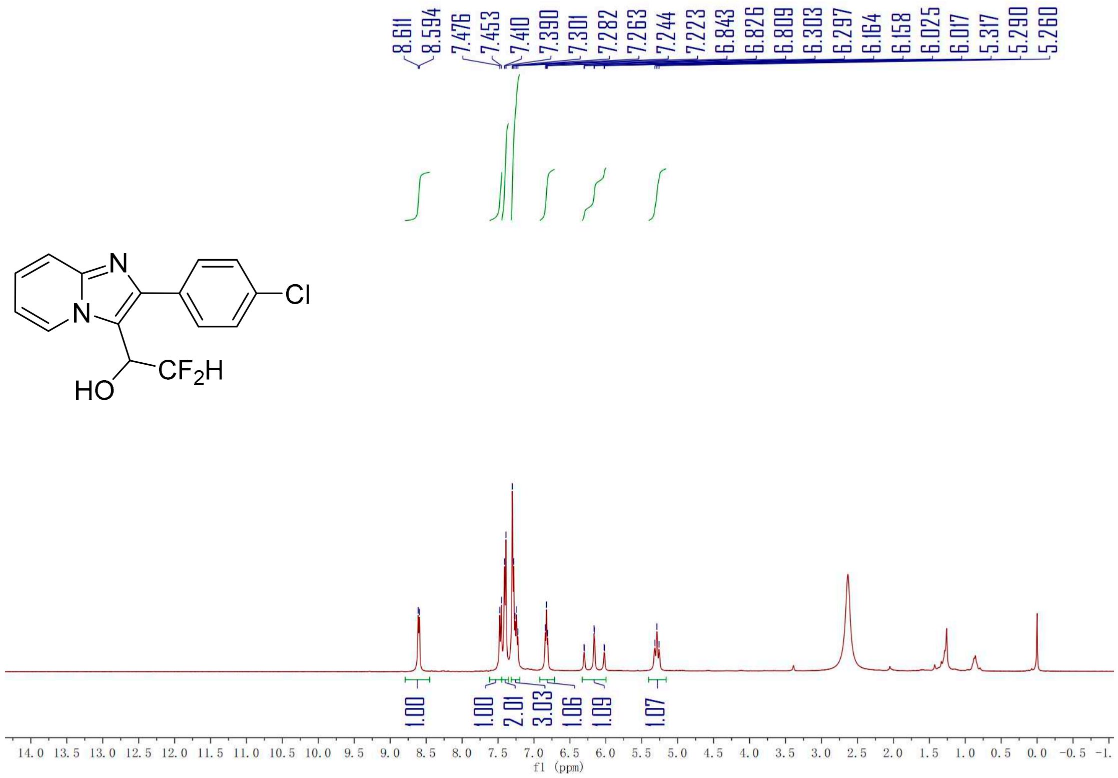

^{1}H NMR spectrum of compound **3i**.

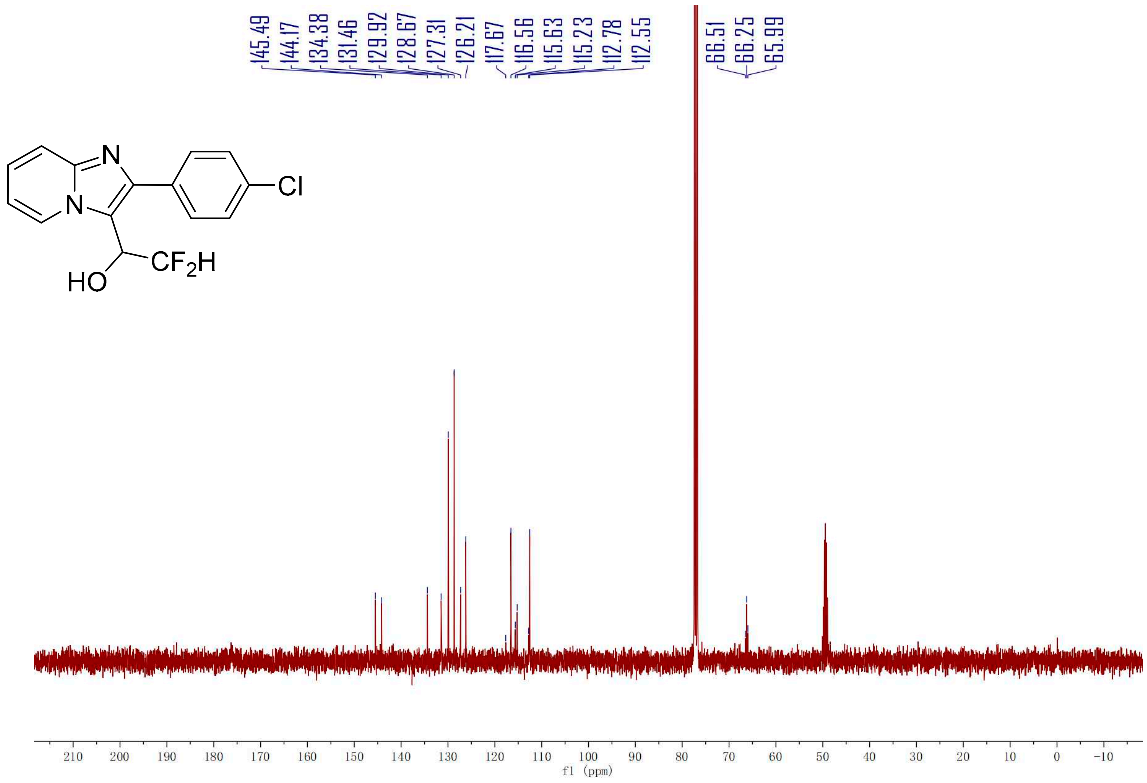

^{13}C NMR spectrum of compound **3i**.

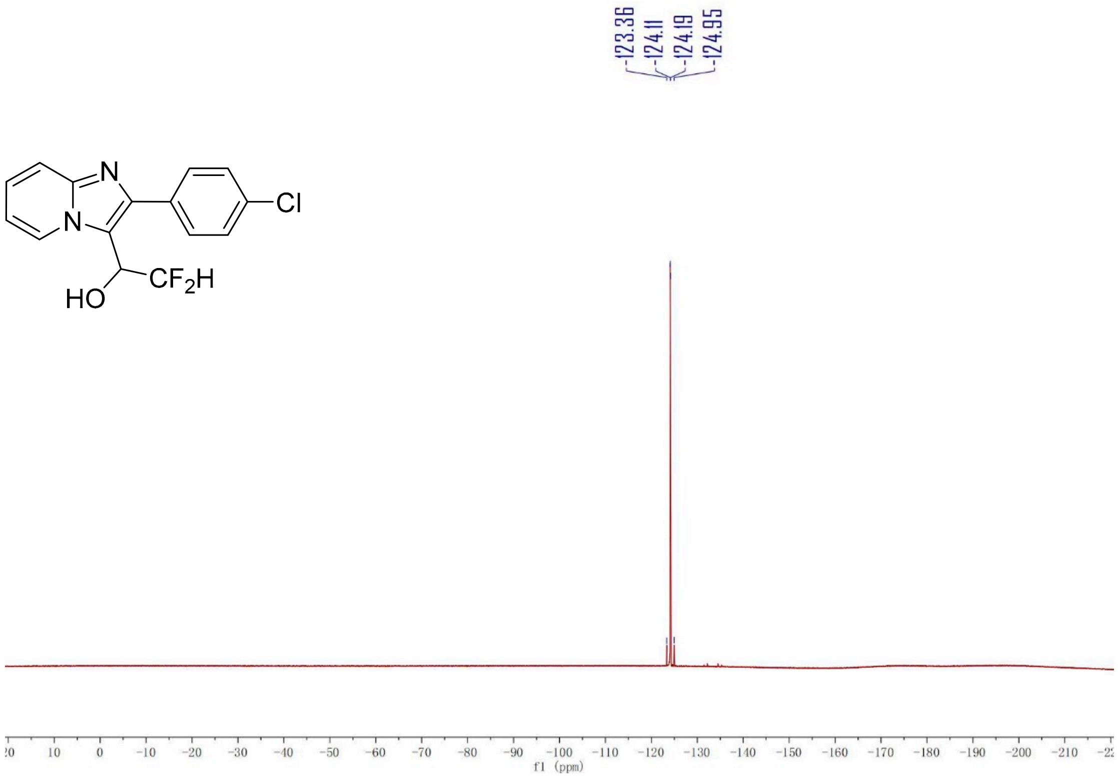

^{19}F NMR spectrum of compound **3i**.

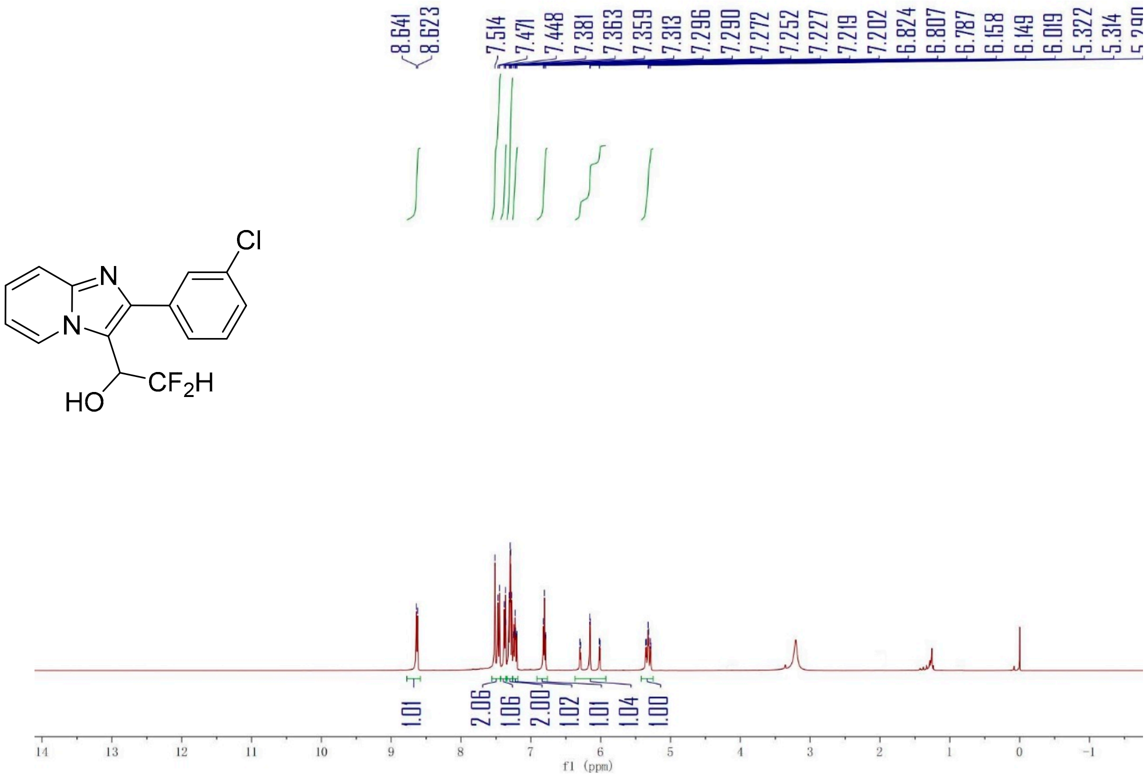

^{1}H NMR spectrum of compound **3j**.

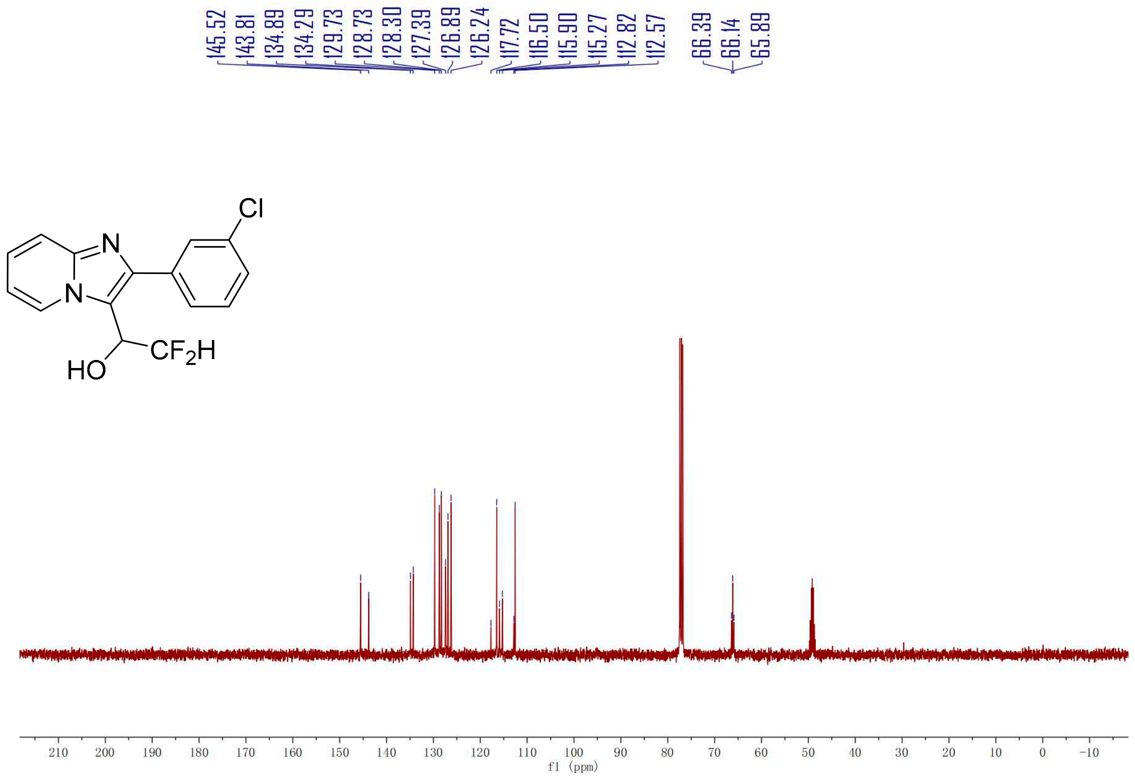

^{13}C NMR spectrum of compound **3j**.

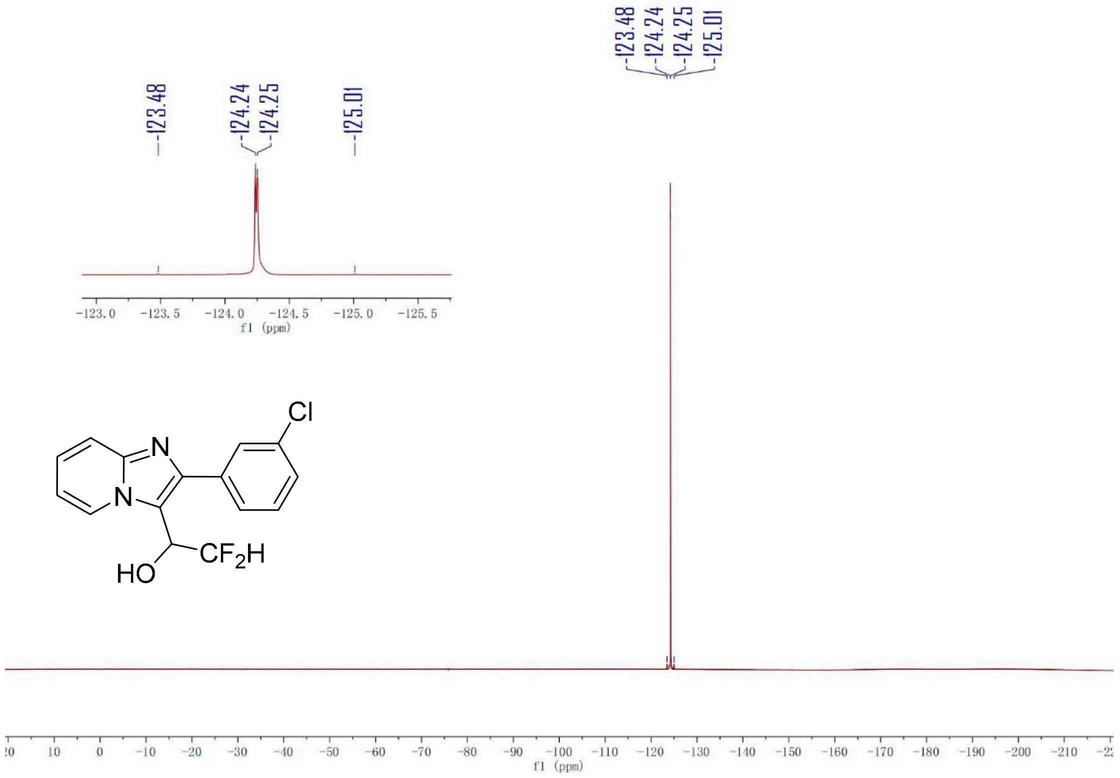

^{19}F NMR spectrum of compound **3j**.

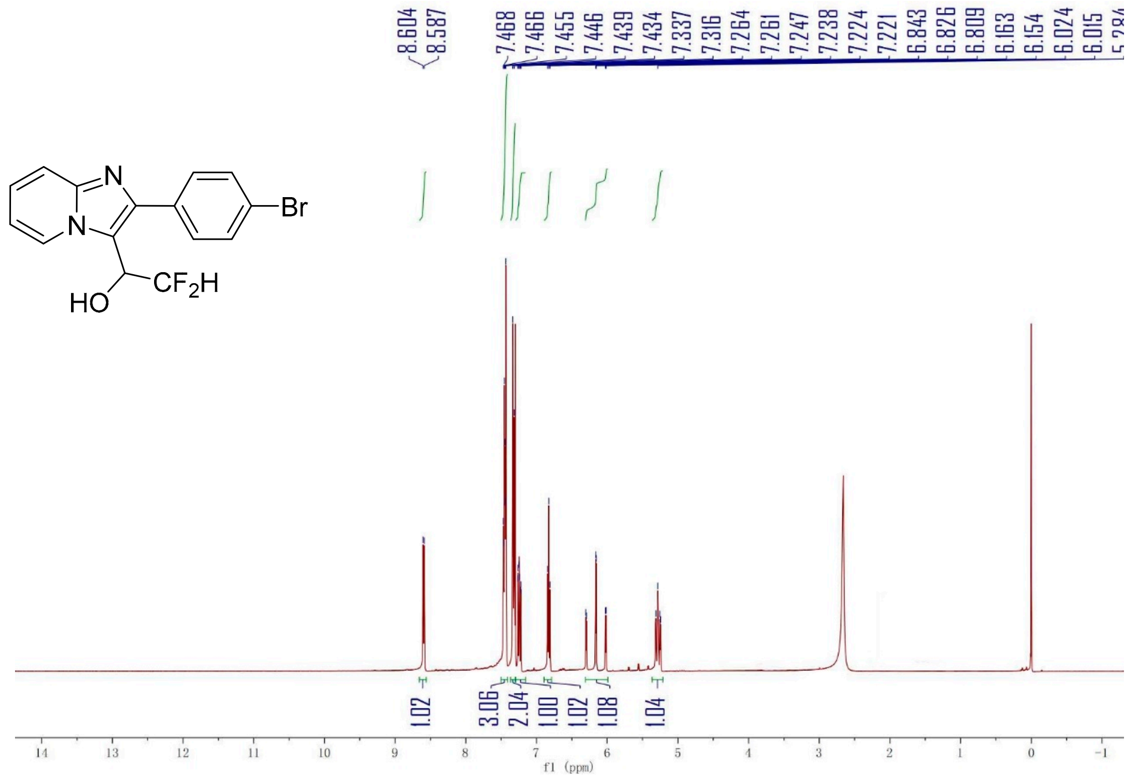

^{1}H NMR spectrum of compound **3k**.

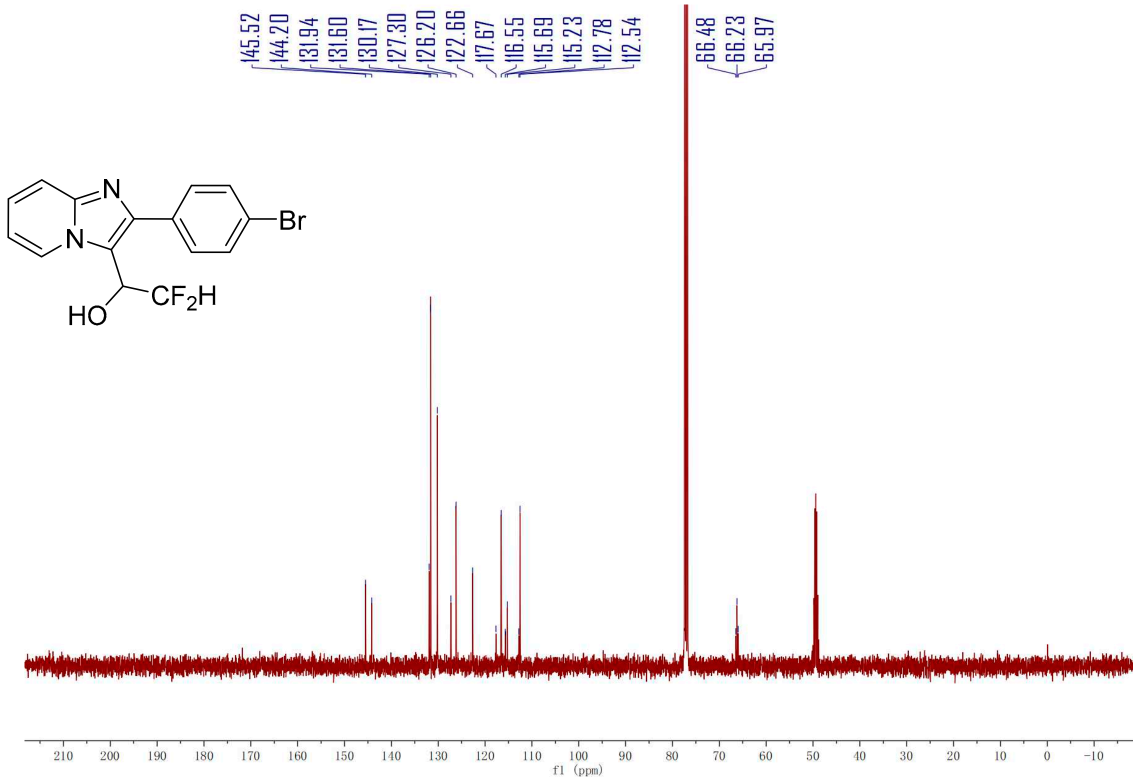

^{13}C NMR spectrum of compound **3k**.

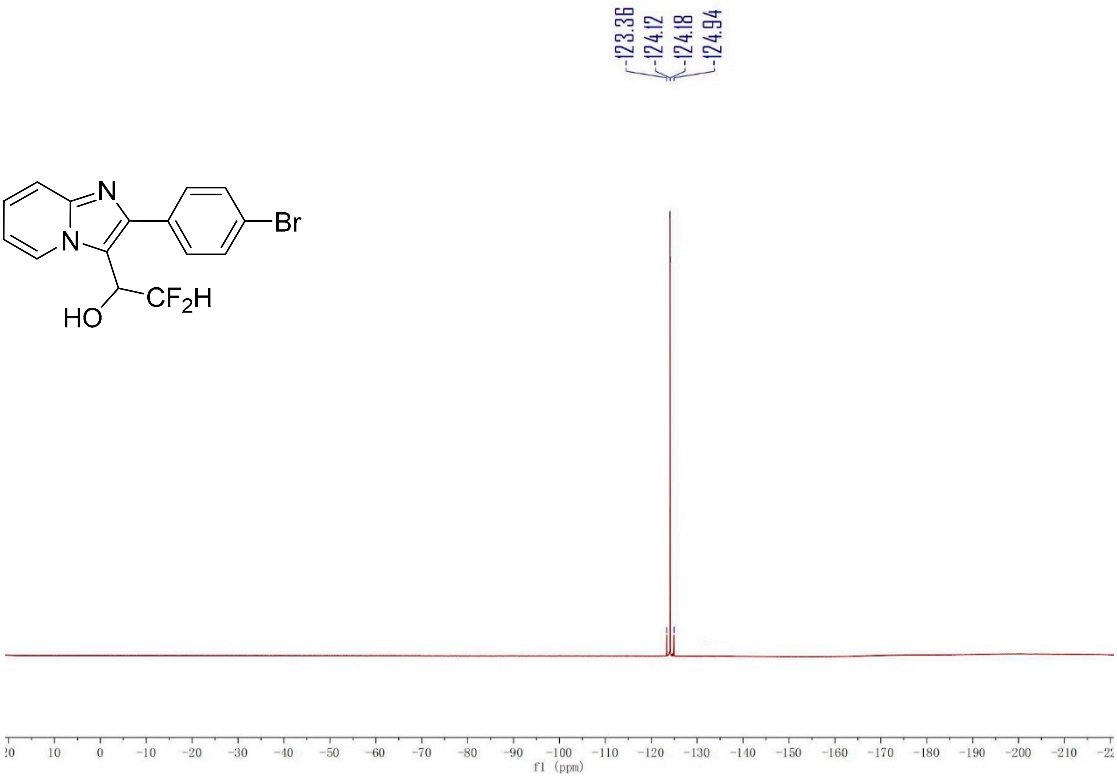

^{19}F NMR spectrum of compound **3k**.

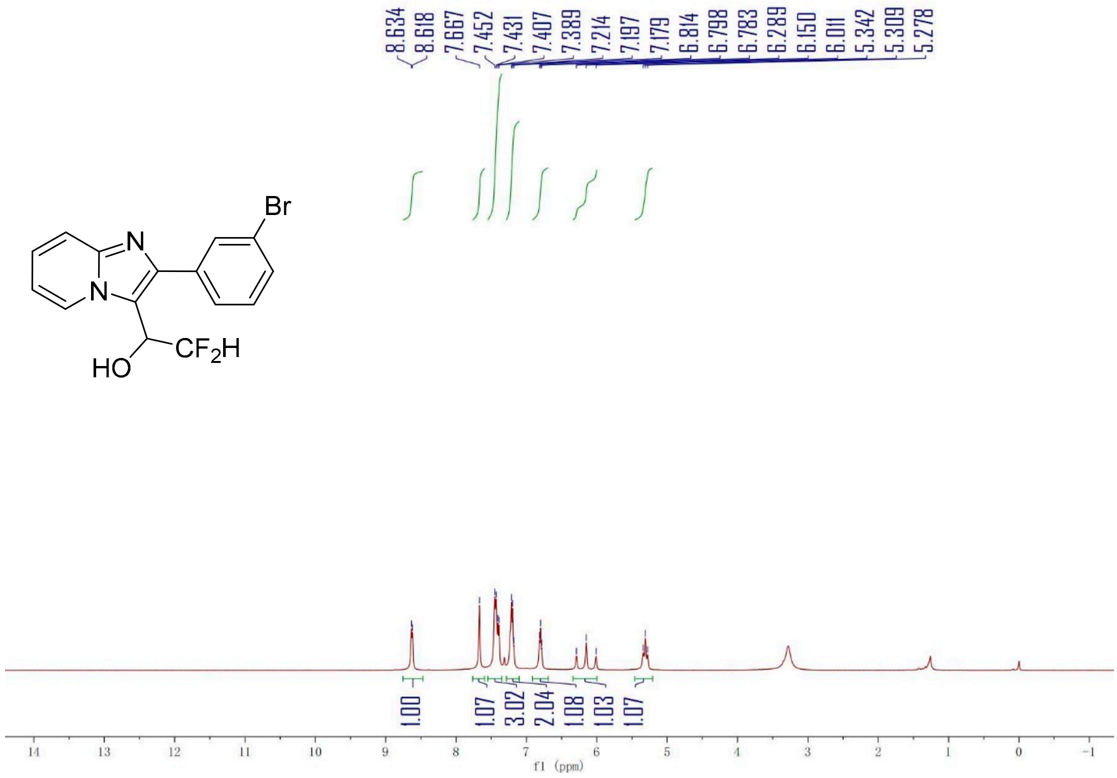

^{1}H NMR spectrum of compound **3l**.

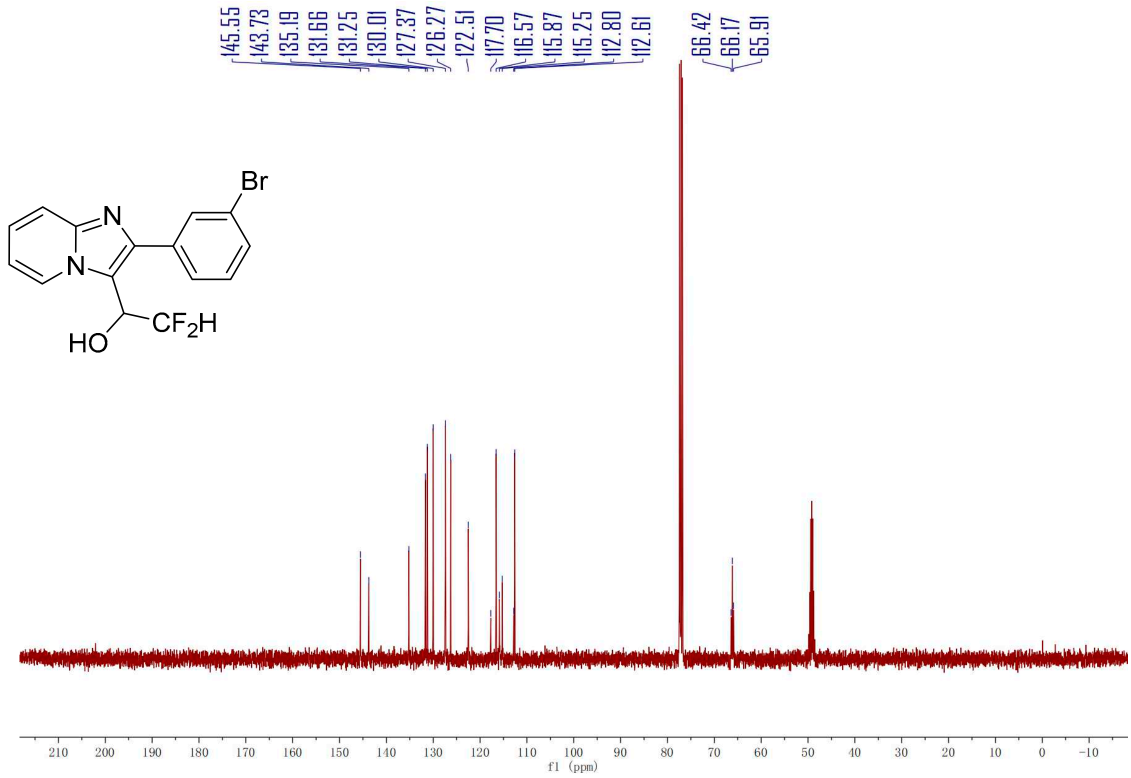

^{13}C NMR spectrum of compound **3l**.

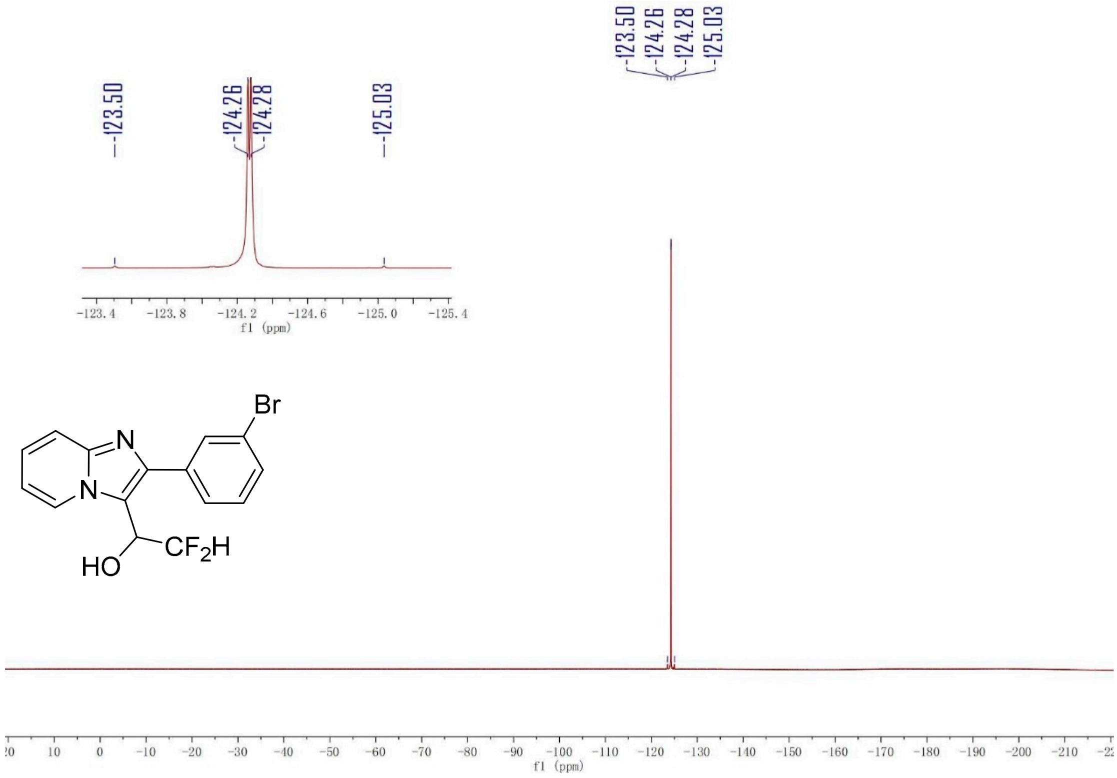

^{19}F NMR spectrum of compound **3l**.

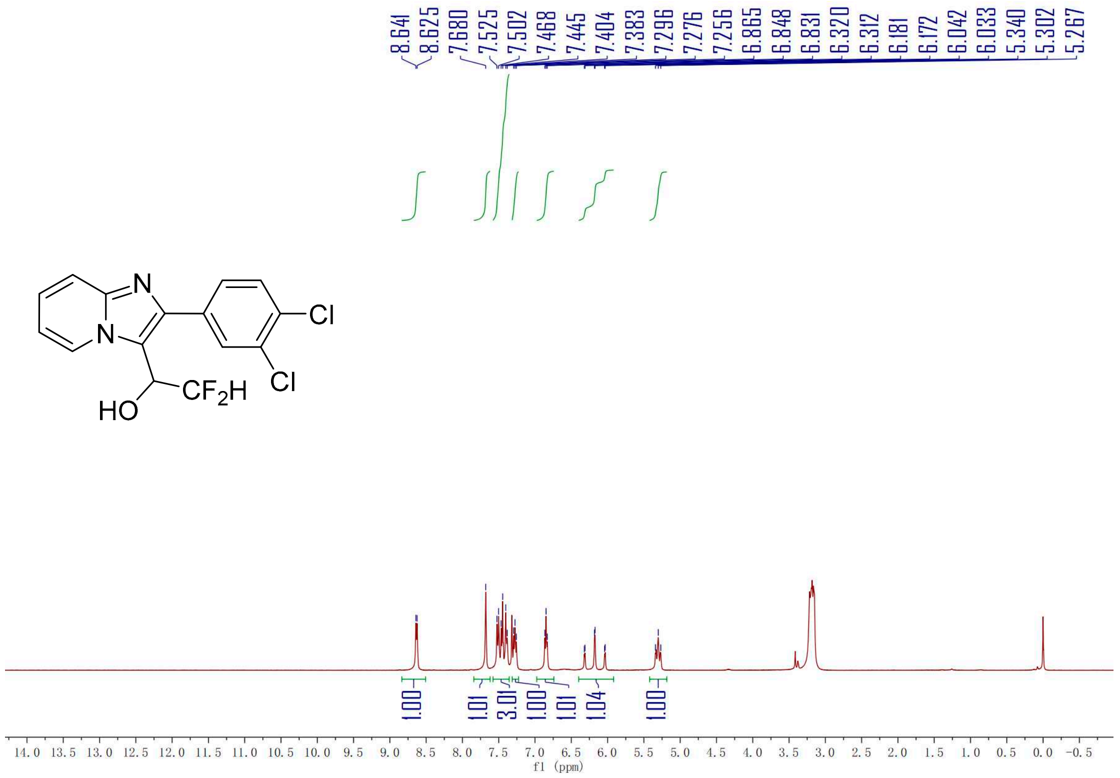

^{1}H NMR spectrum of compound **3m**.

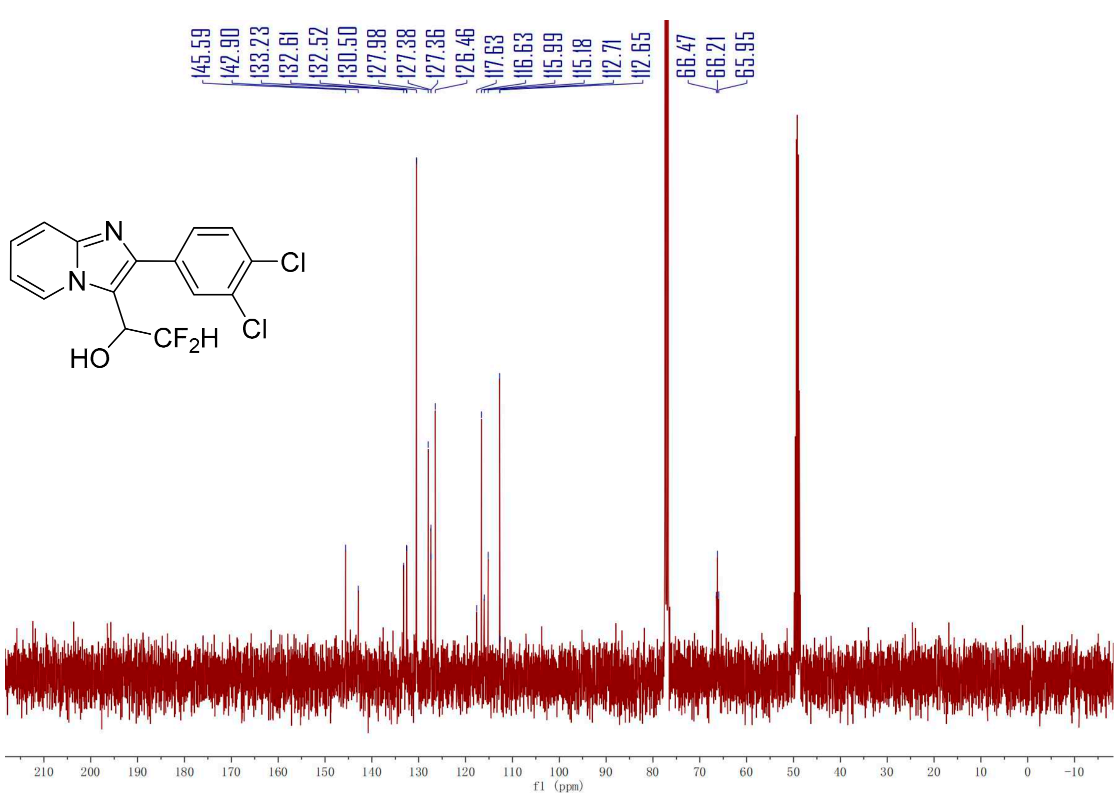

^{13}C NMR spectrum of compound **3m**.

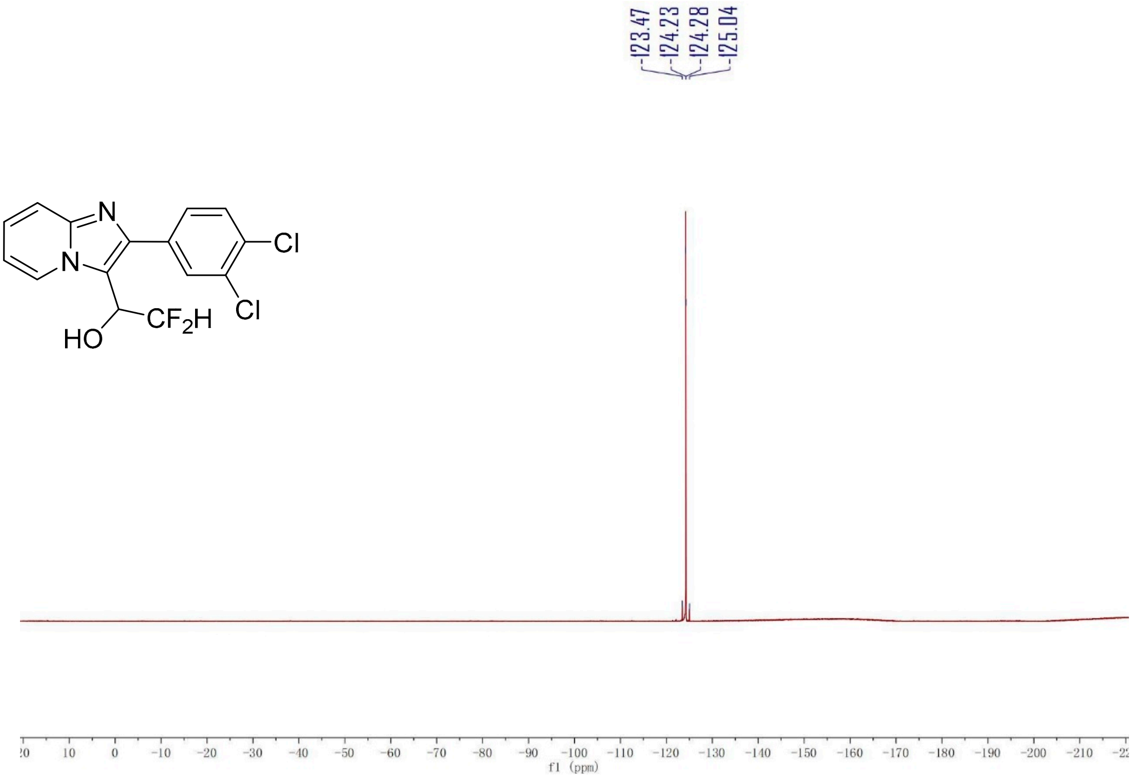

^{19}F NMR spectrum of compound **3m**.

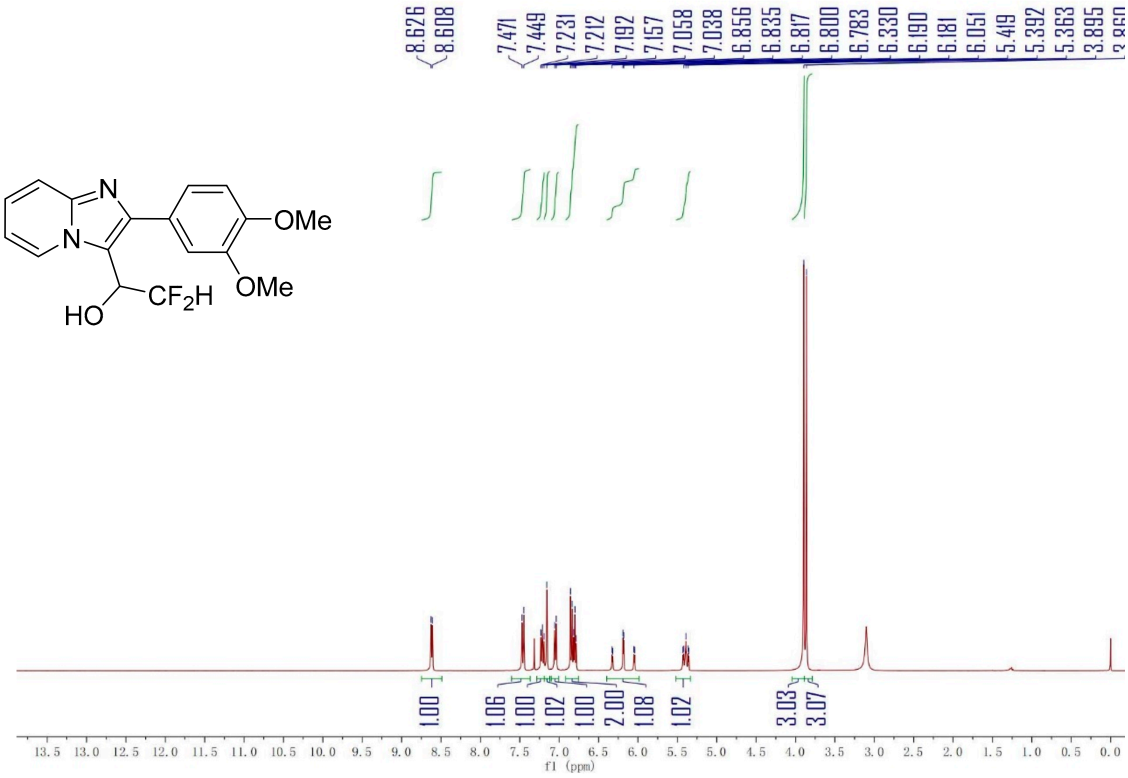

^{1}H NMR spectrum of compound **3n**.

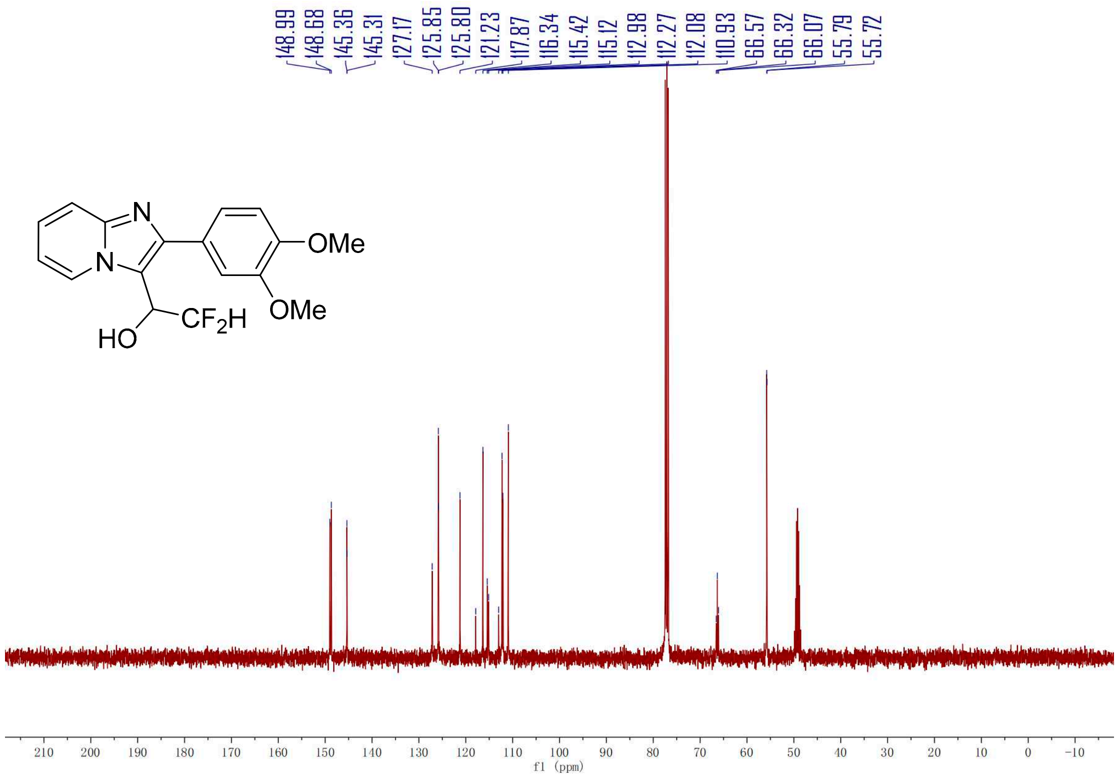

^{13}C NMR spectrum of compound **3n**.

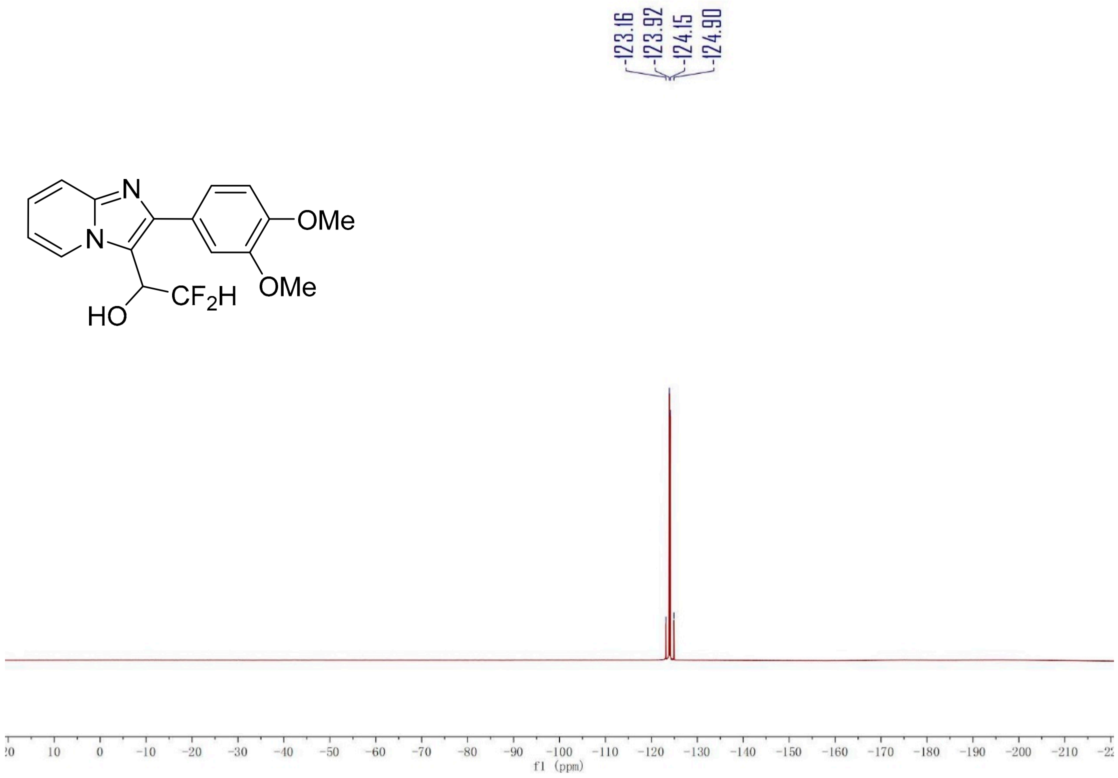

^{19}F NMR spectrum of compound **3n**.

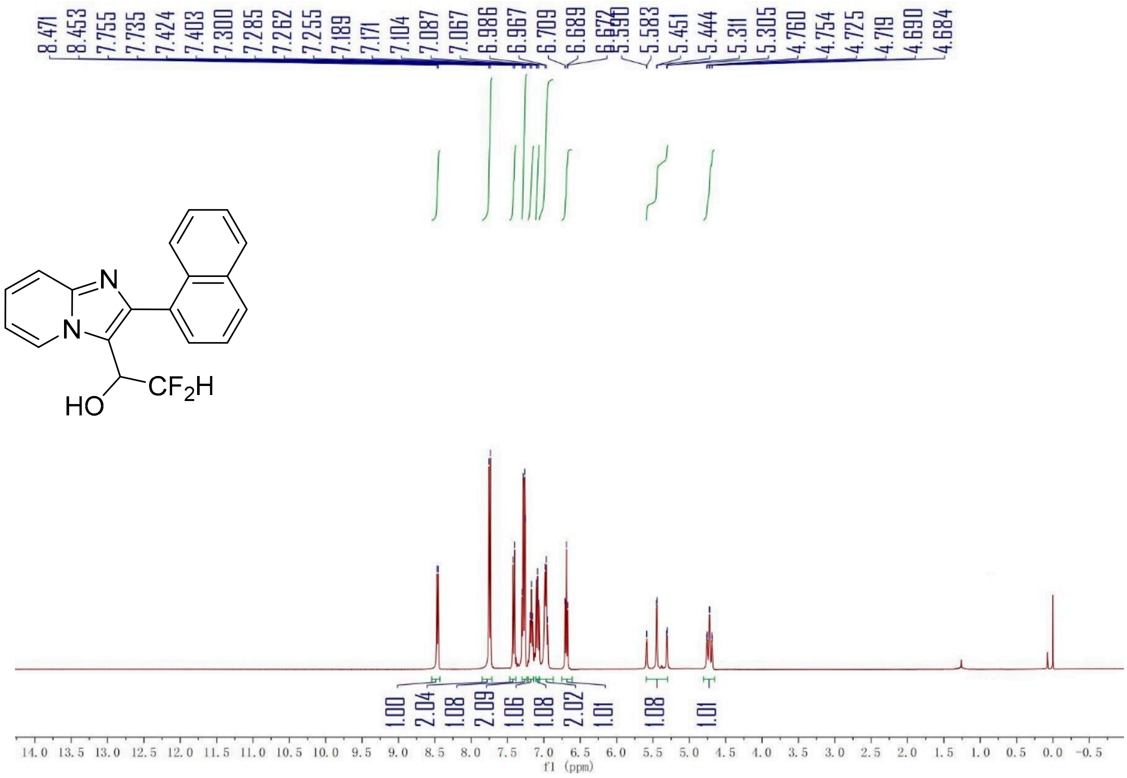

^{1}H NMR spectrum of compound **3o**.

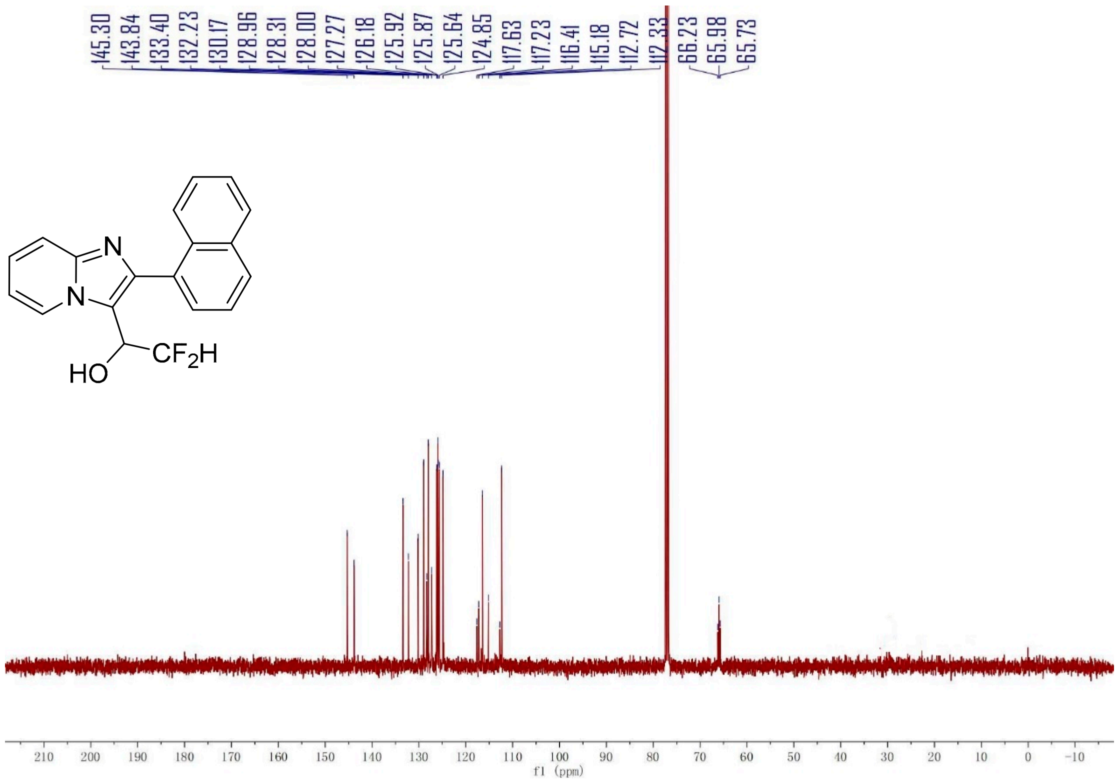

^{13}C NMR spectrum of compound **3o**.

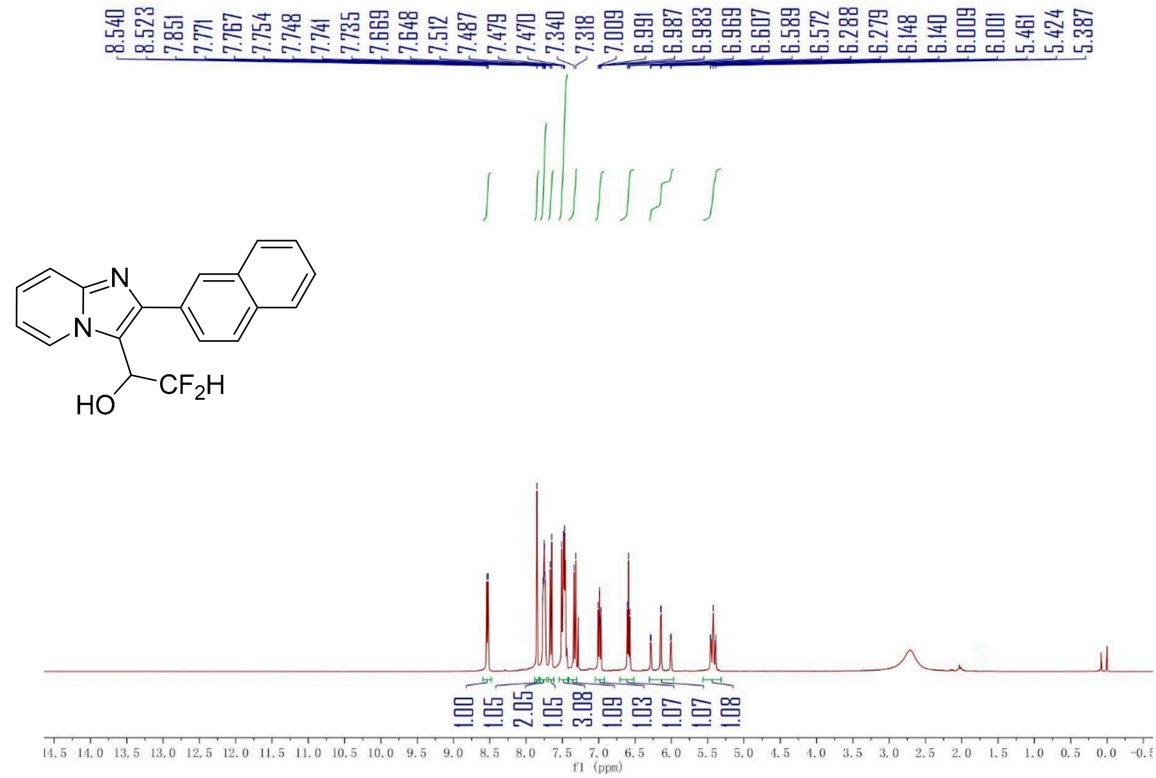

^{1}H NMR spectrum of compound **3p**.

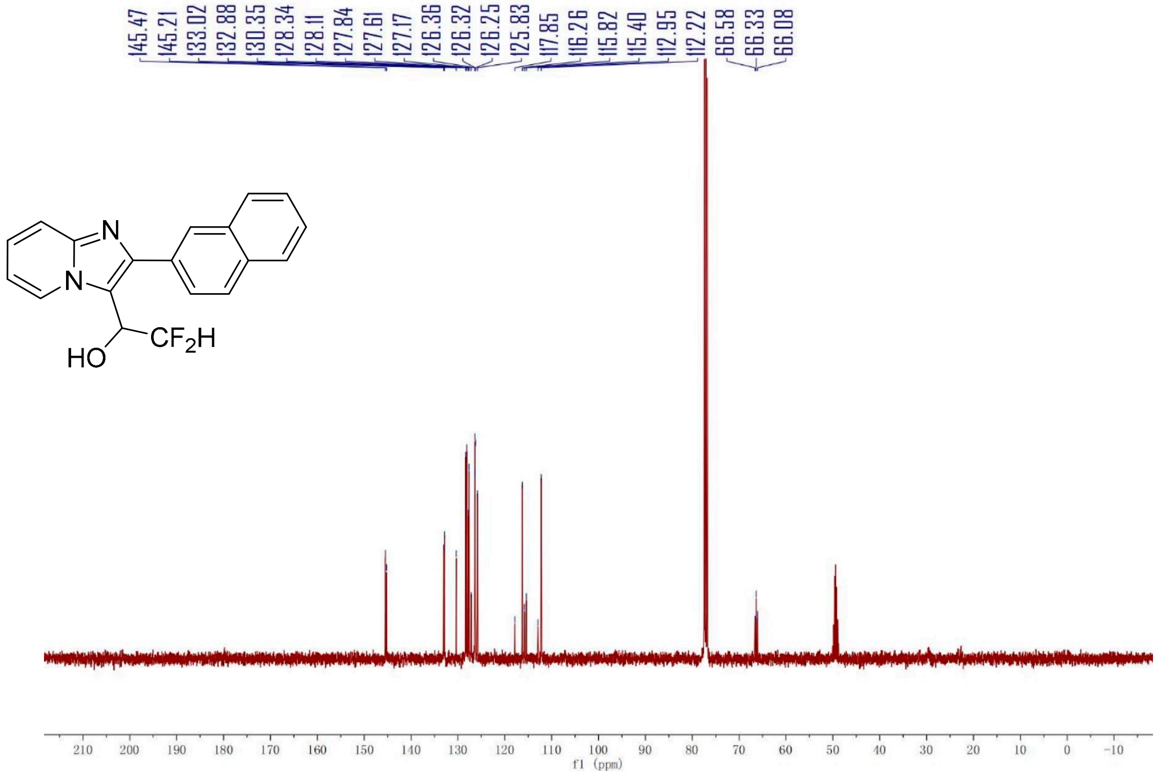

^{13}C NMR spectrum of compound **3p**.

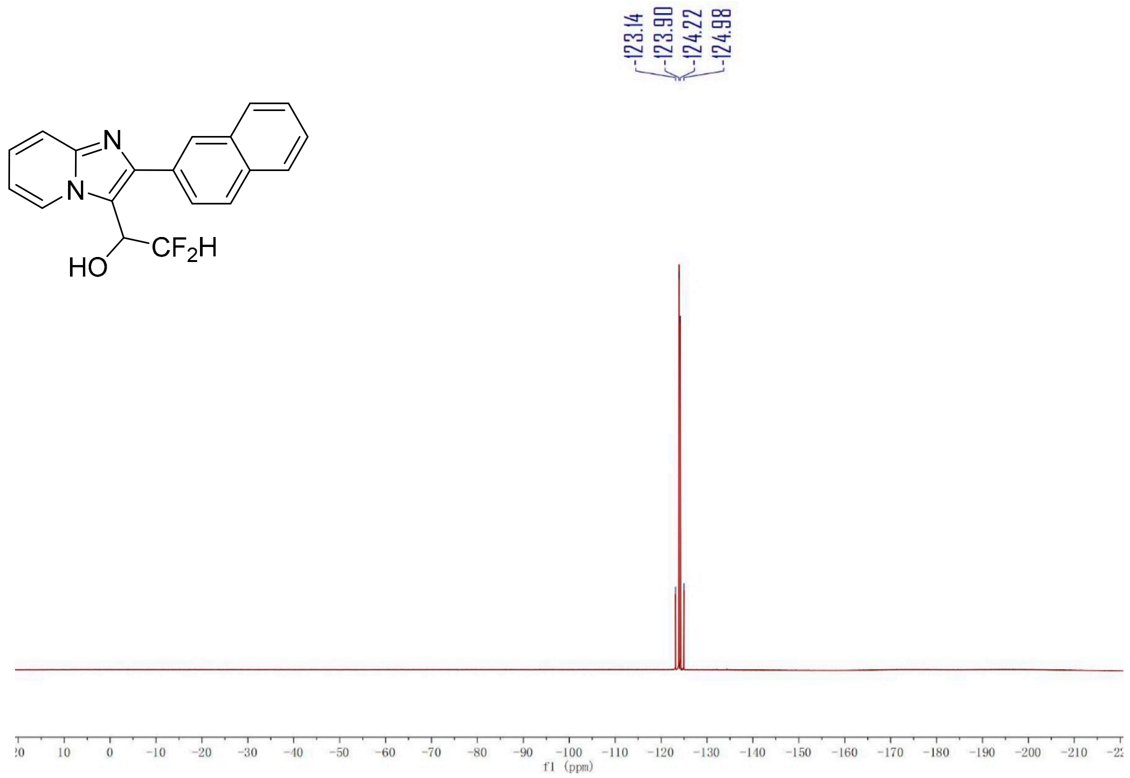

^{19}F NMR spectrum of compound **3p**.

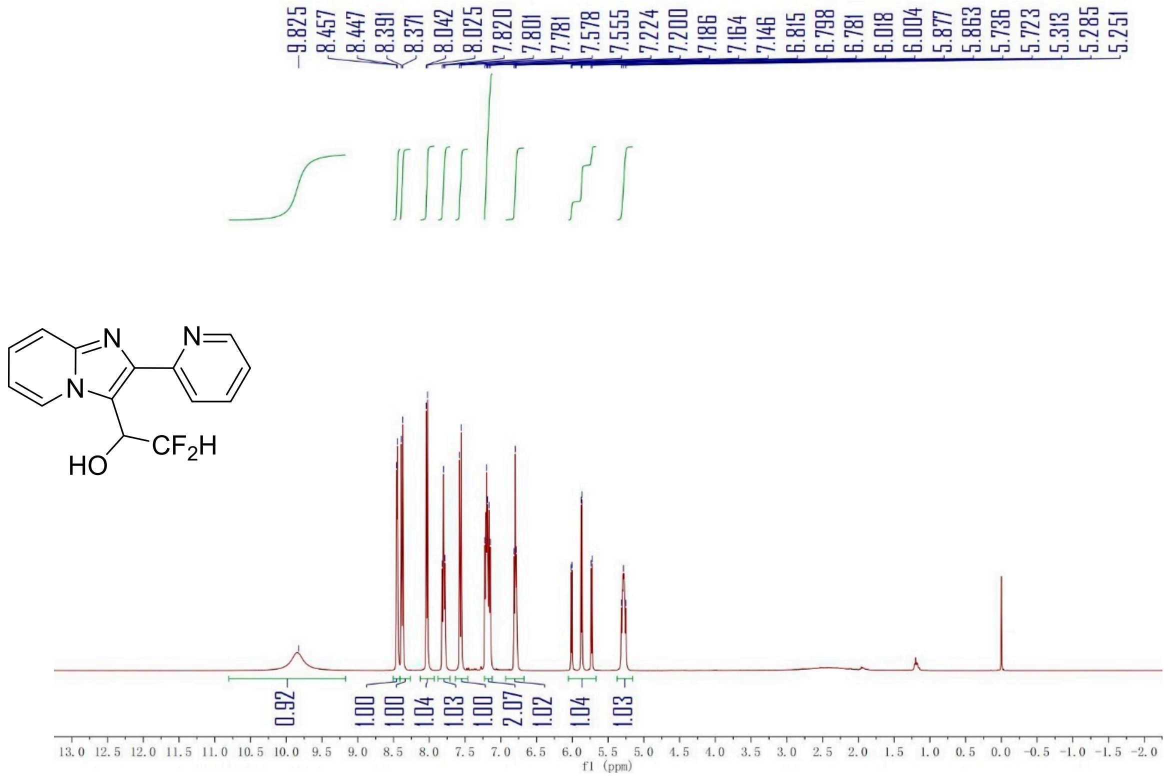

^{1}H NMR spectrum of compound **3q**.

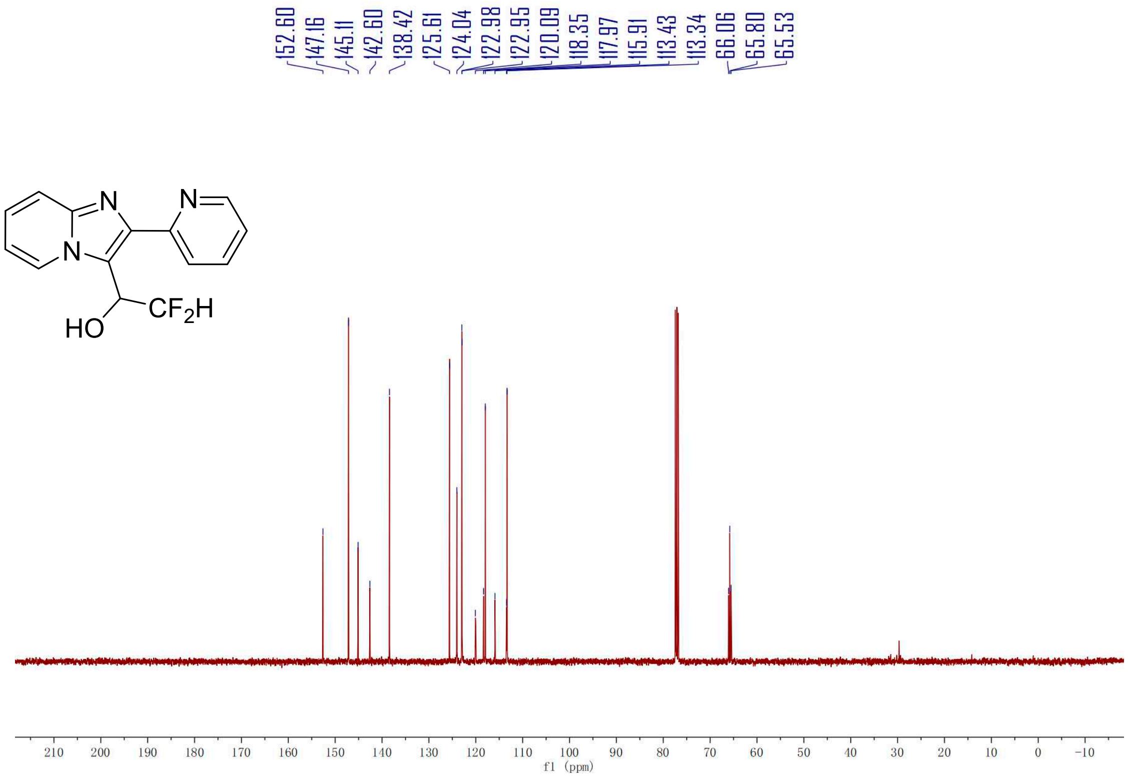

^{13}C NMR spectrum of compound **3q**.

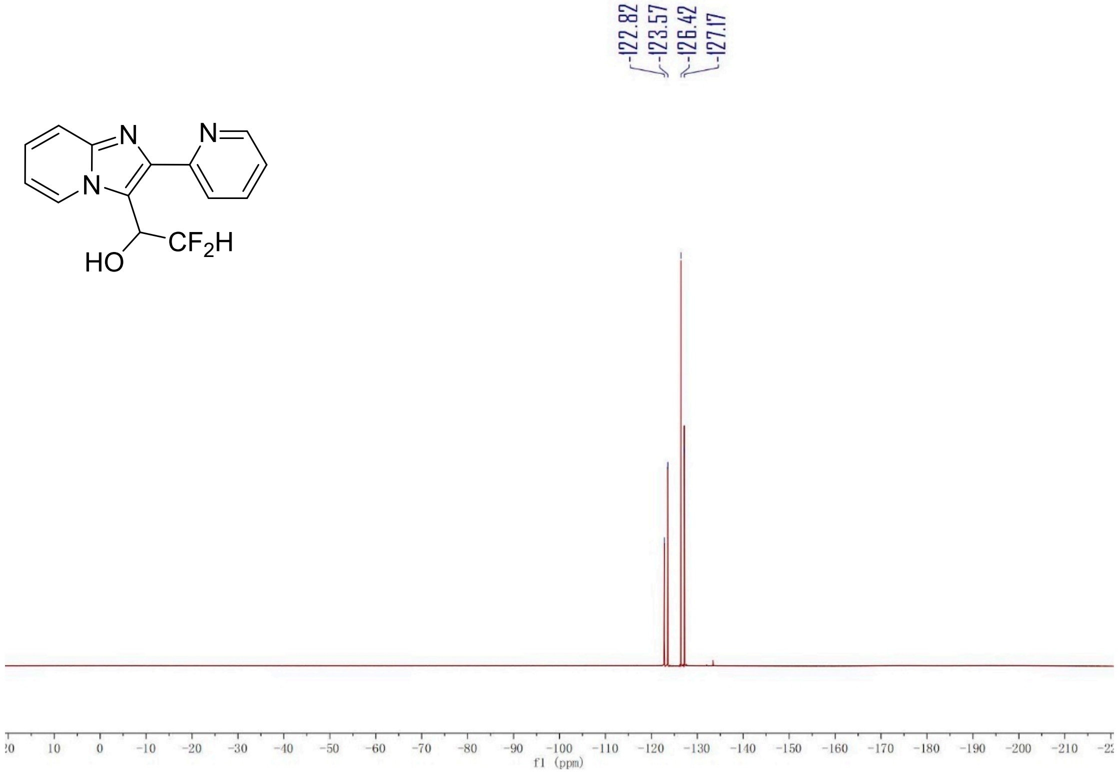

^{19}F NMR spectrum of compound **3q**.

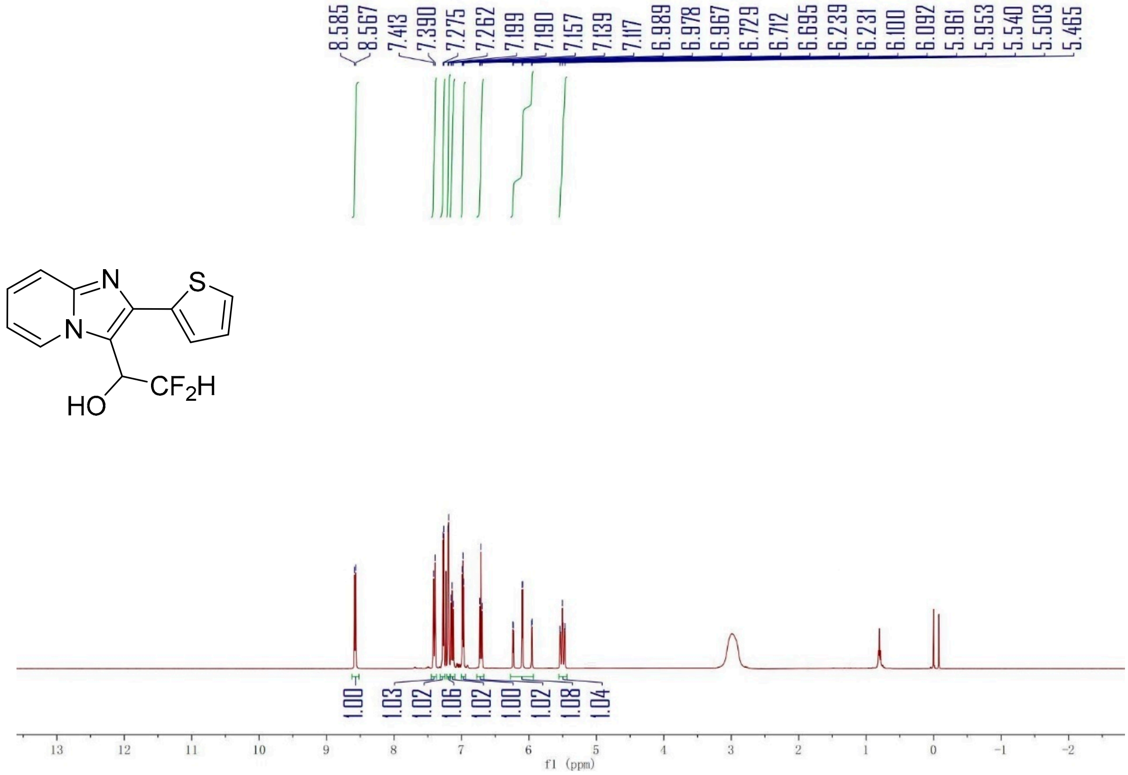

^{1}H NMR spectrum of compound **3r**.

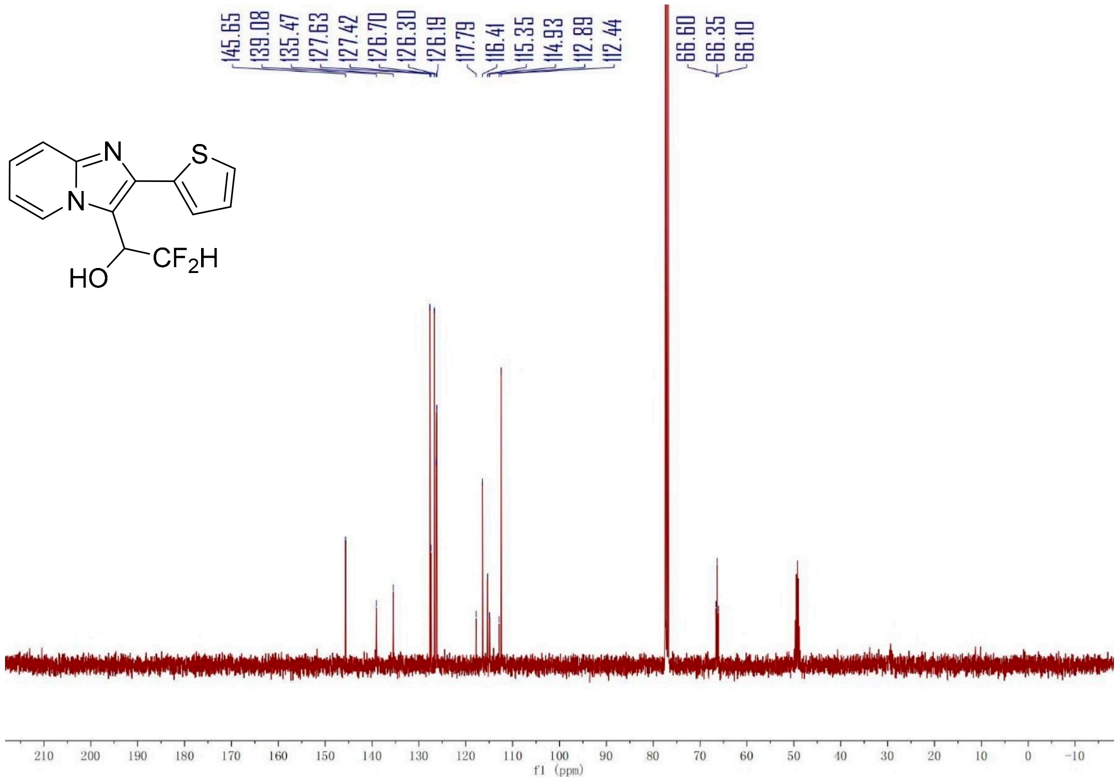

^{13}C NMR spectrum of compound **3r**.

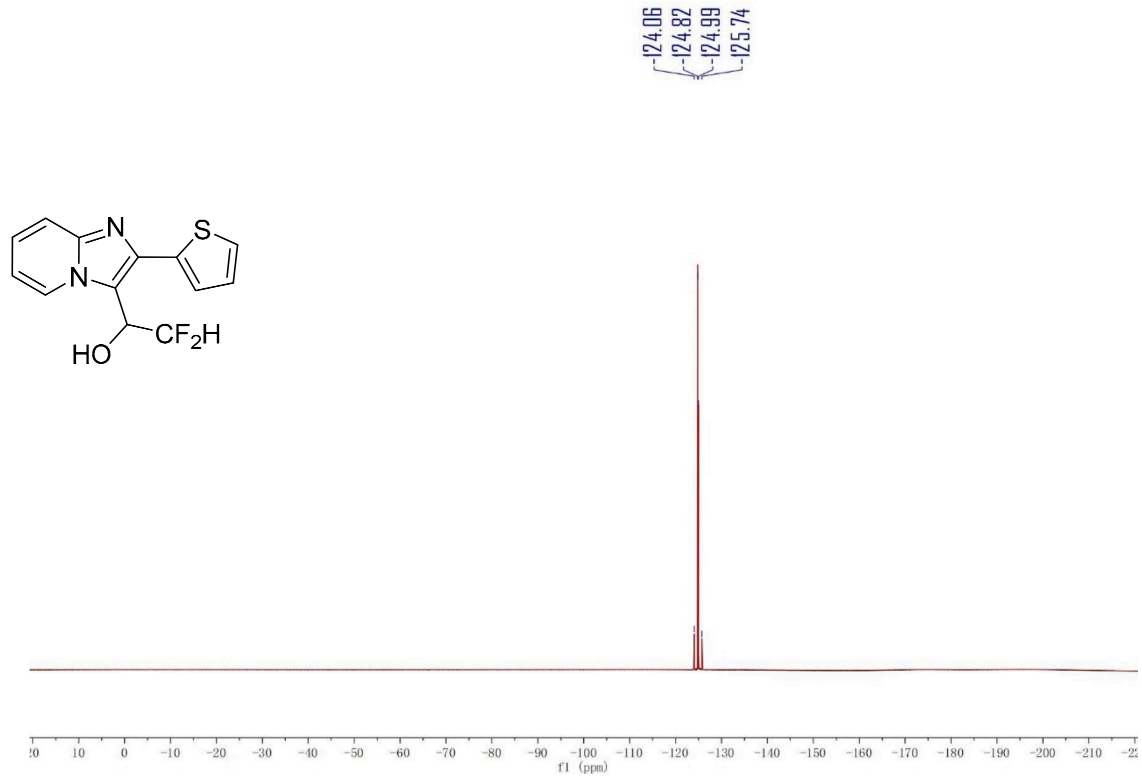

^{19}F NMR spectrum of compound **3r**.

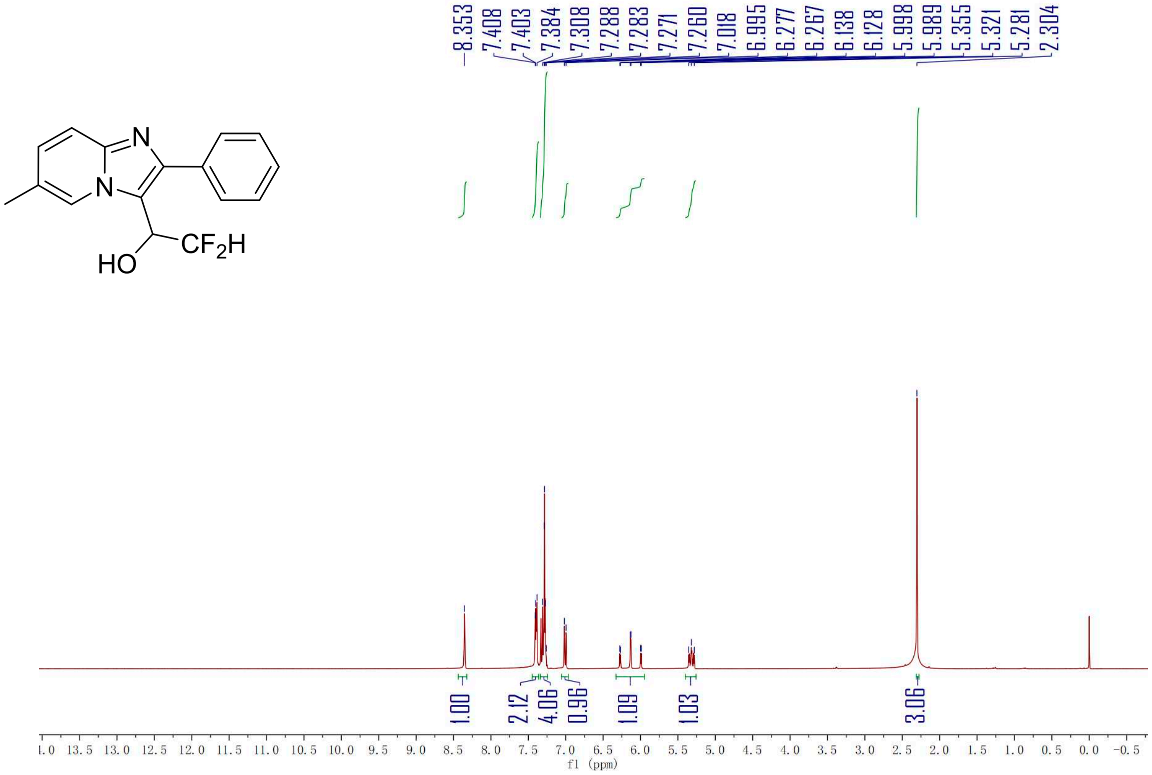

^{1}H NMR spectrum of compound **3aa**.

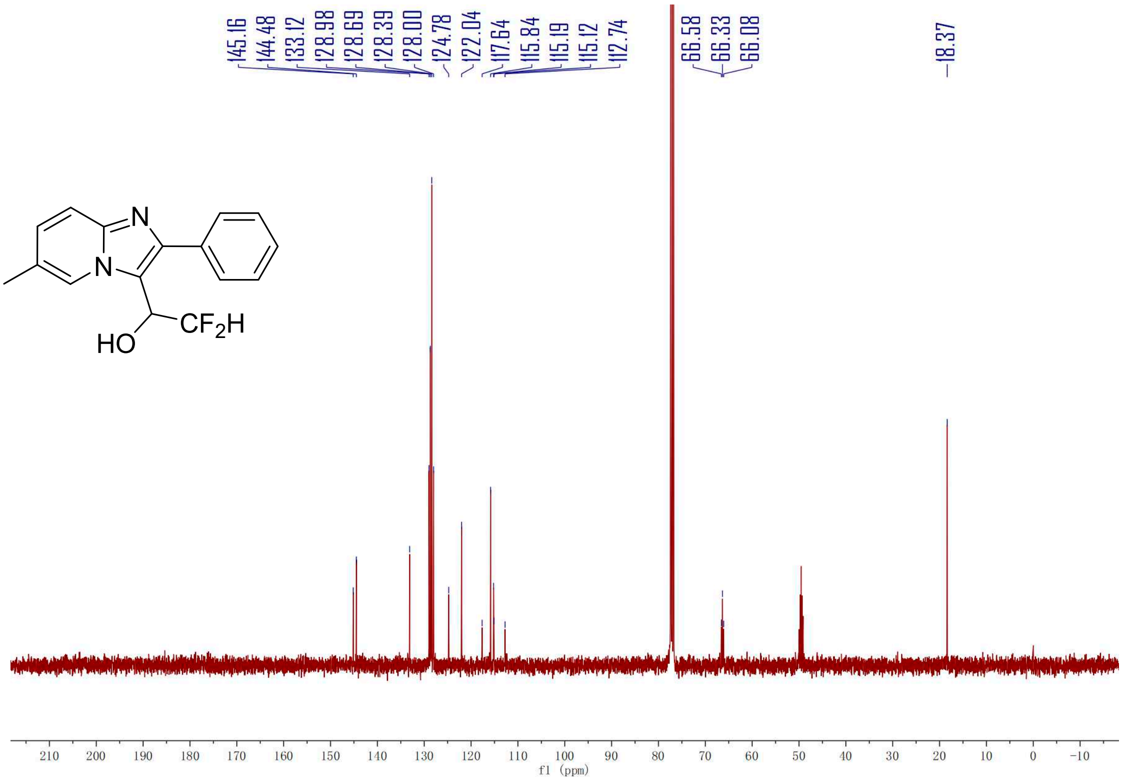

^{13}C NMR spectrum of compound **3aa**.

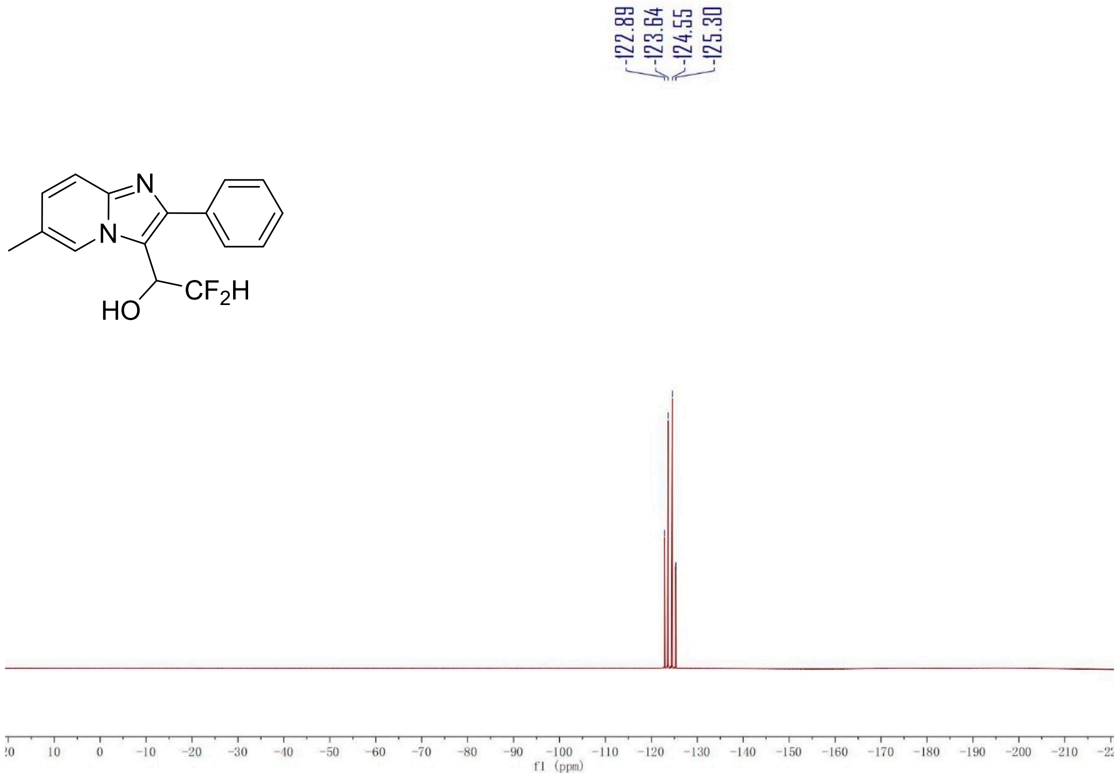

^{19}F NMR spectrum of compound **3aa**.

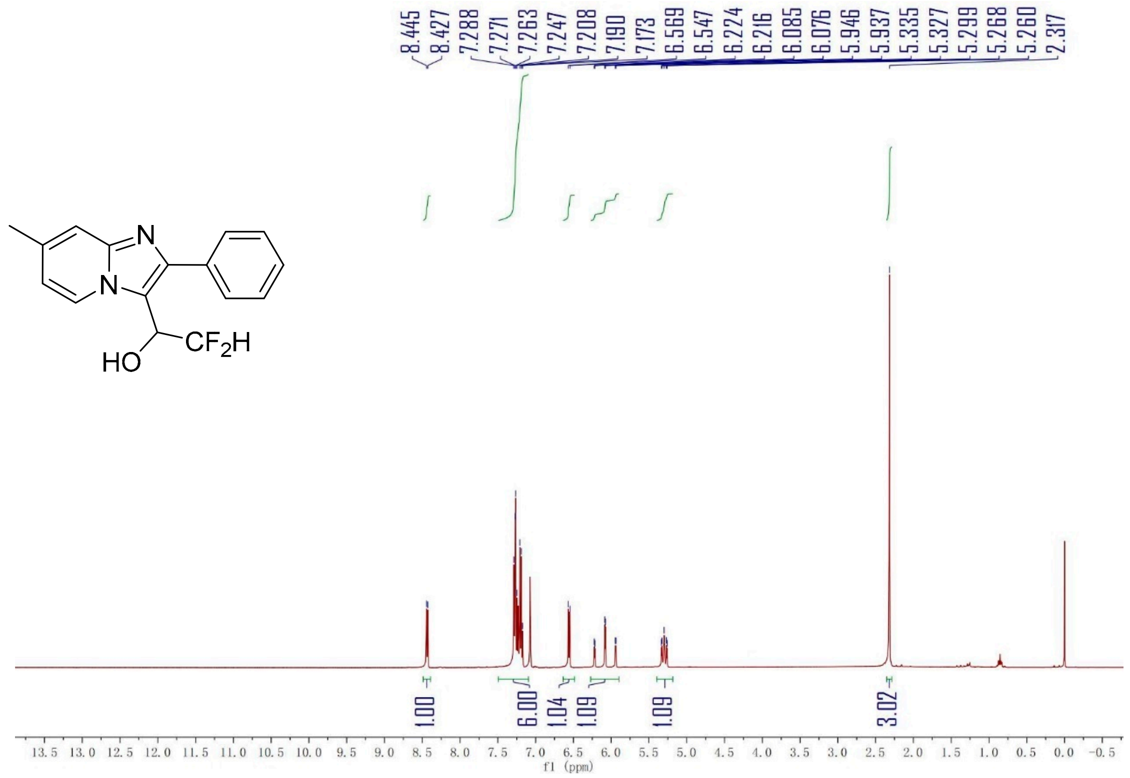

^{1}H NMR spectrum of compound **3ab**.

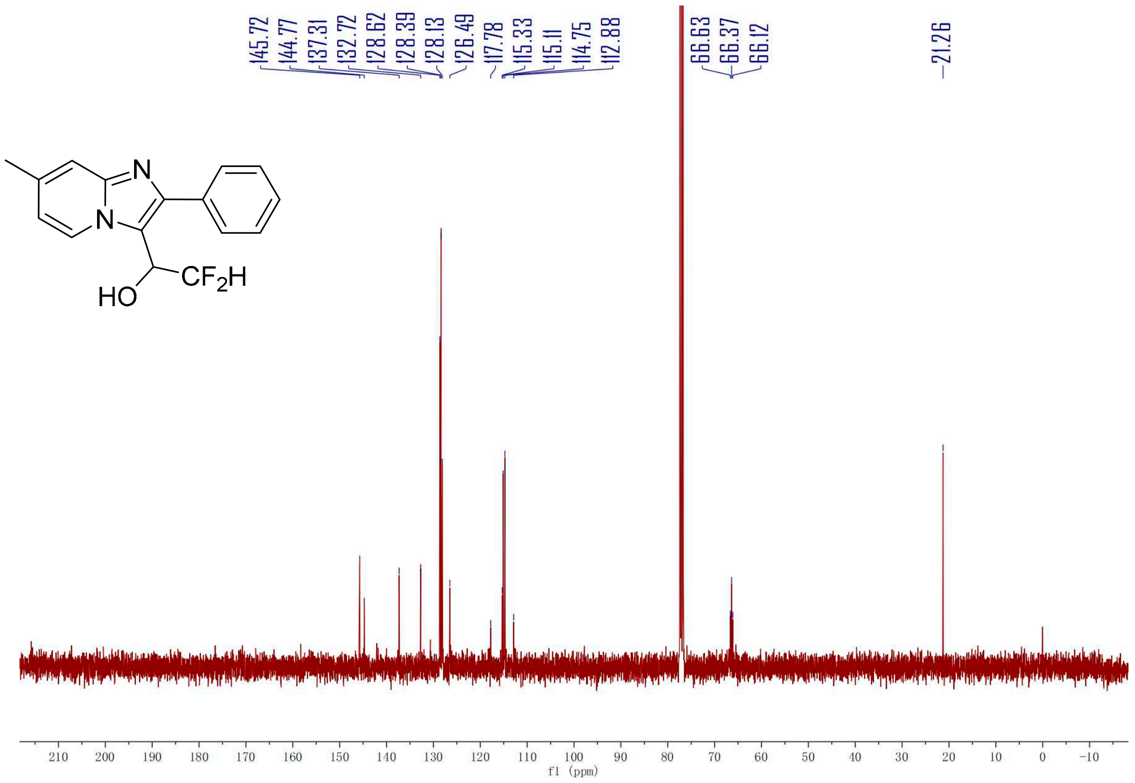

^{13}C NMR spectrum of compound **3ab**.

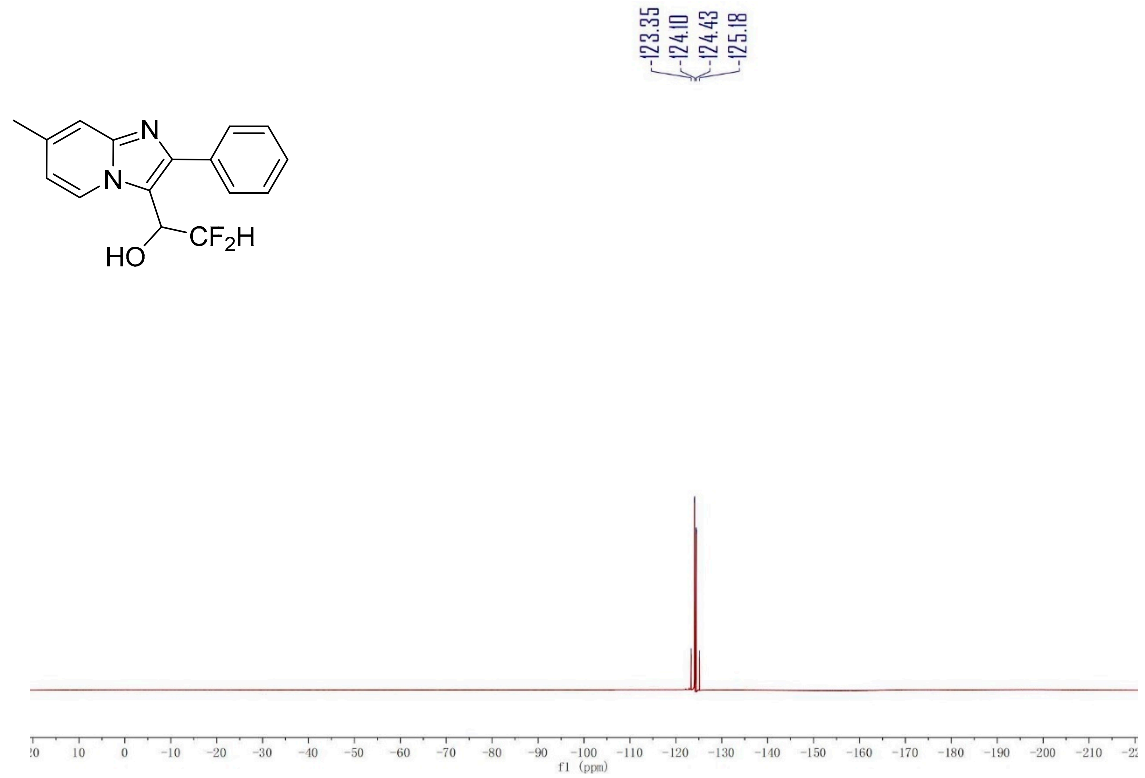

^{19}F NMR spectrum of compound **3b**.

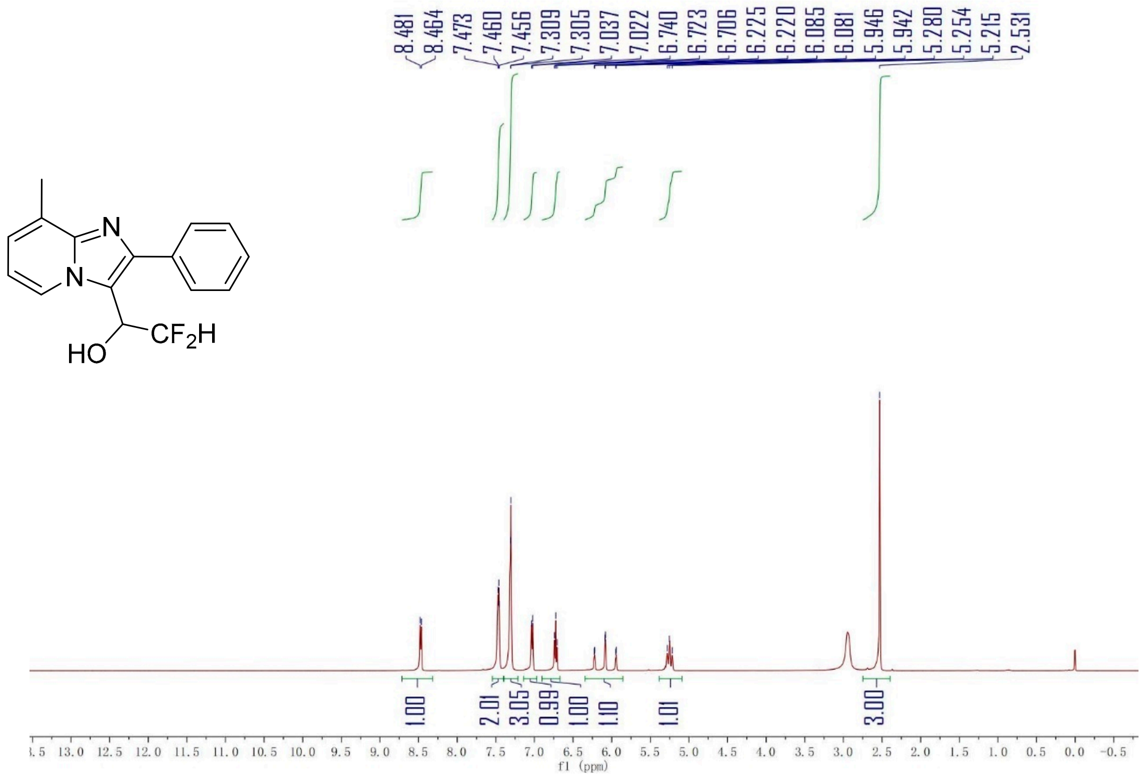

^{1}H NMR spectrum of compound **3ac**.

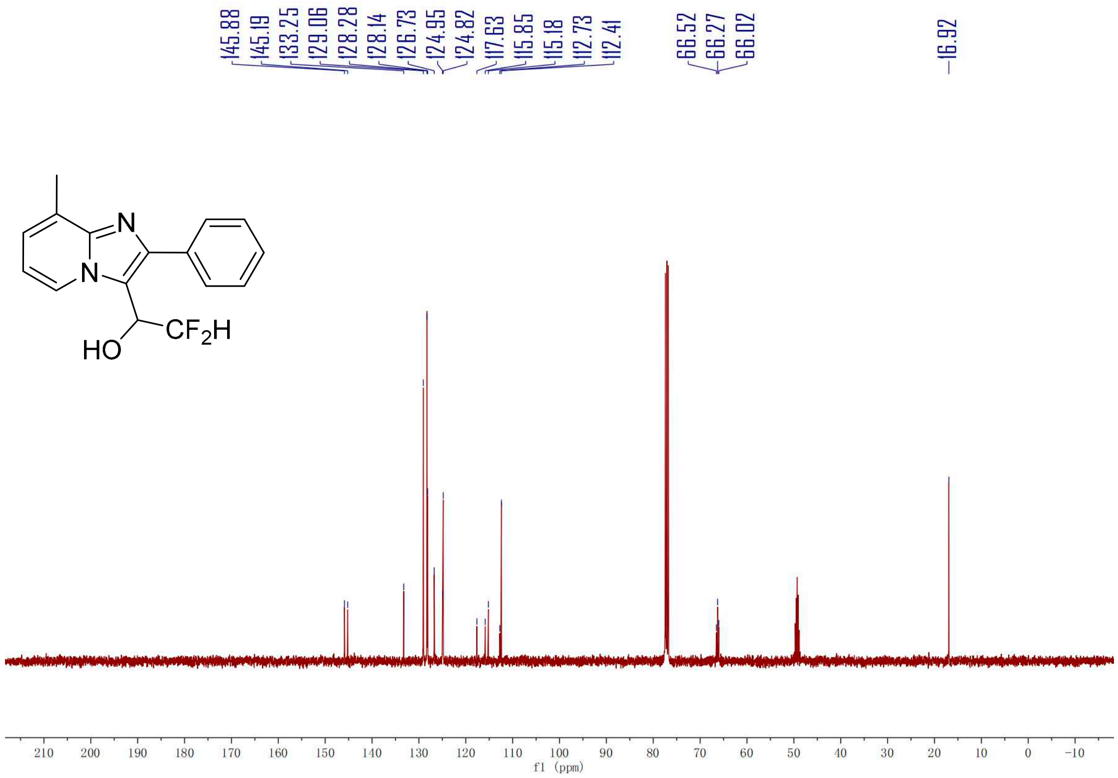

^{13}C NMR spectrum of compound **3ac**.

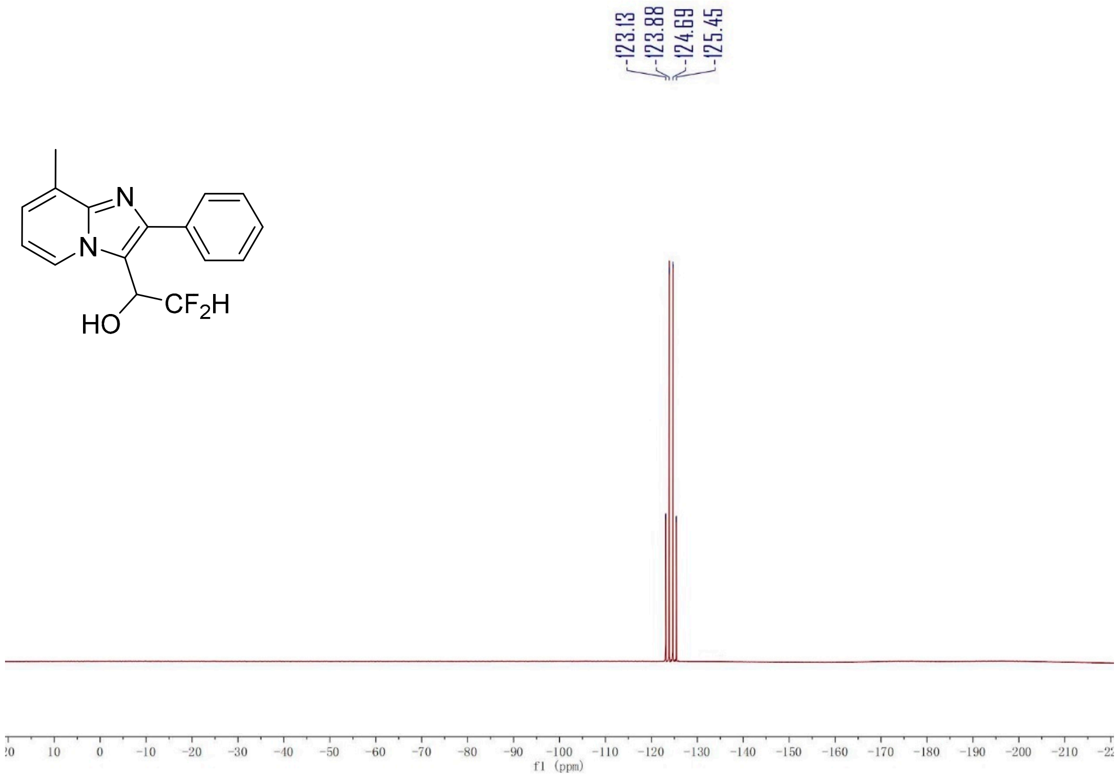

^{19}F NMR spectrum of compound **3ac**.

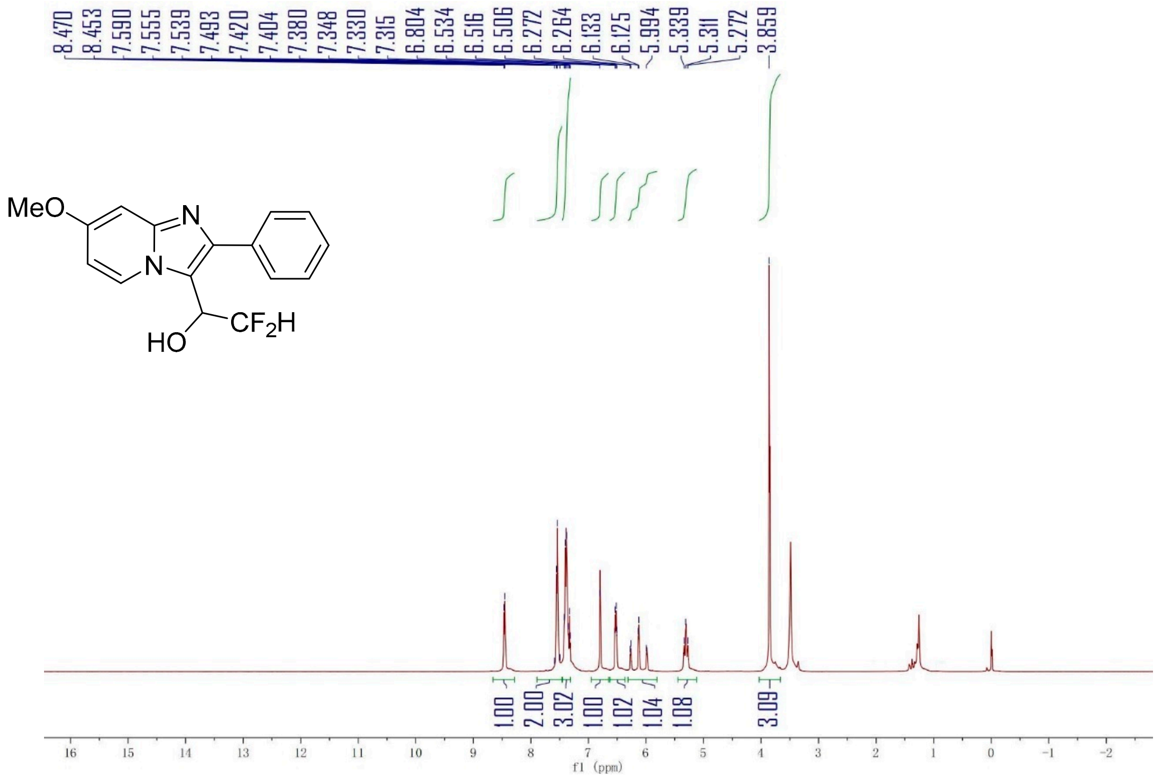

^{1}H NMR spectrum of compound **3ad**.

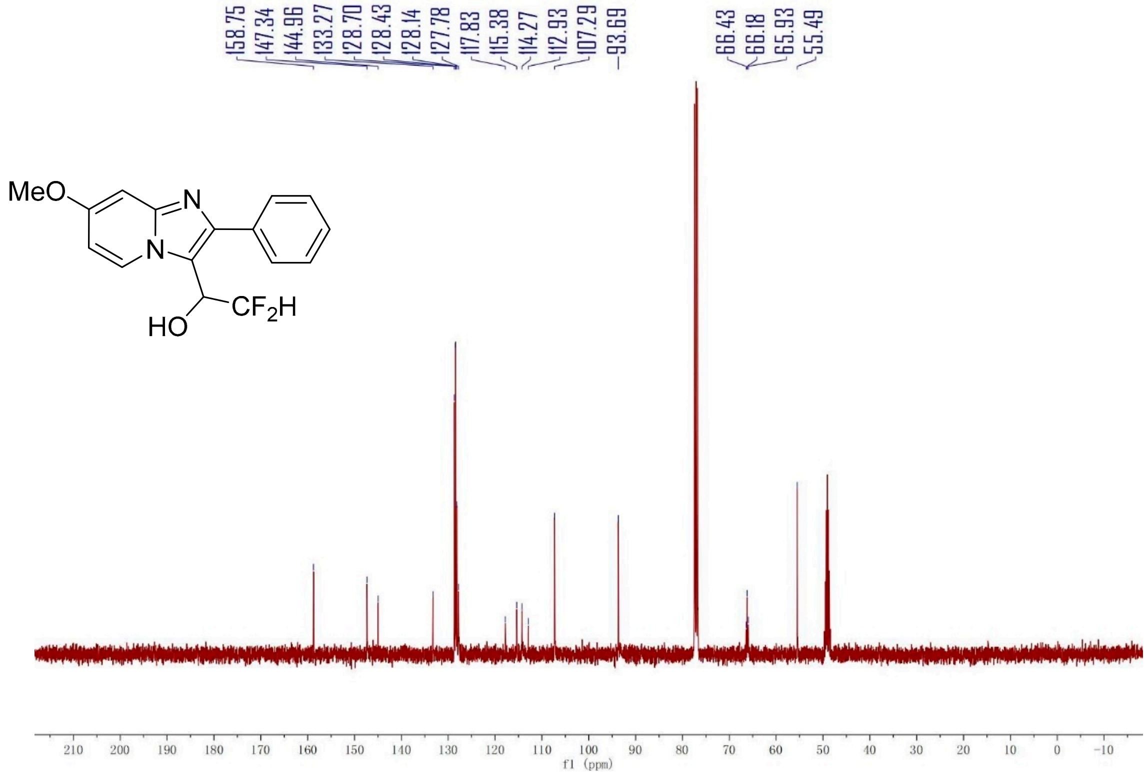

^{13}C NMR spectrum of compound **3ad**.

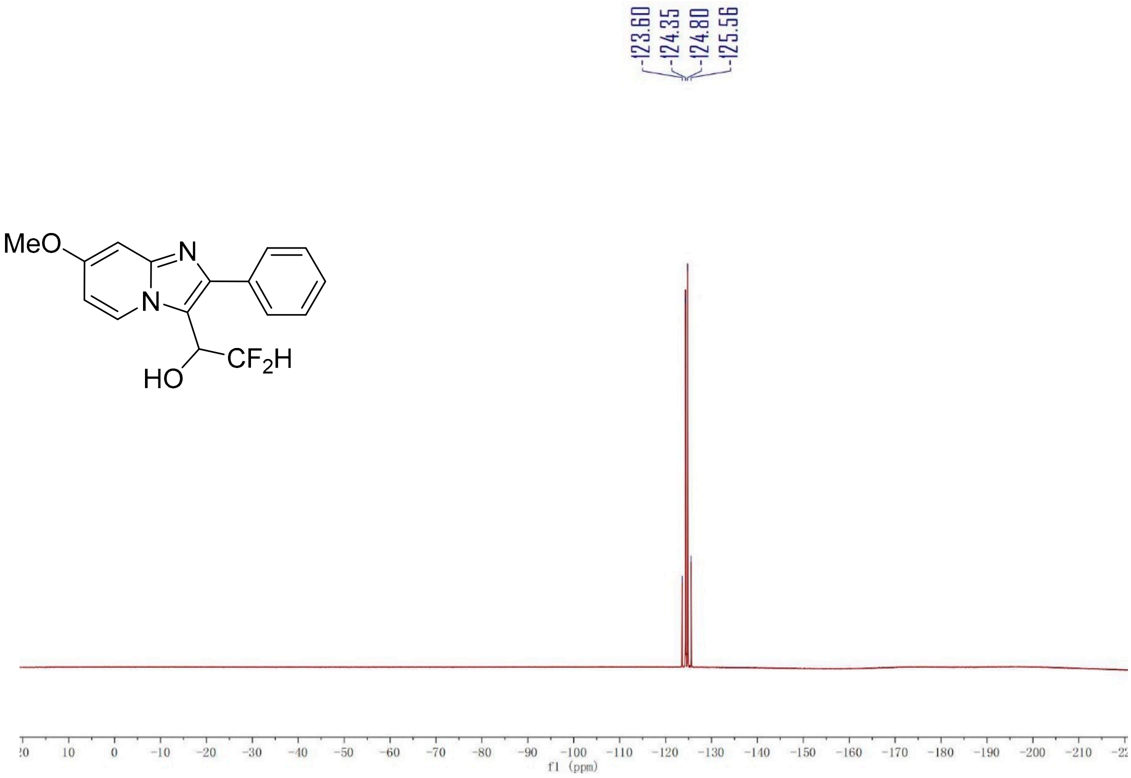

^{19}F NMR spectrum of compound **3ad**.

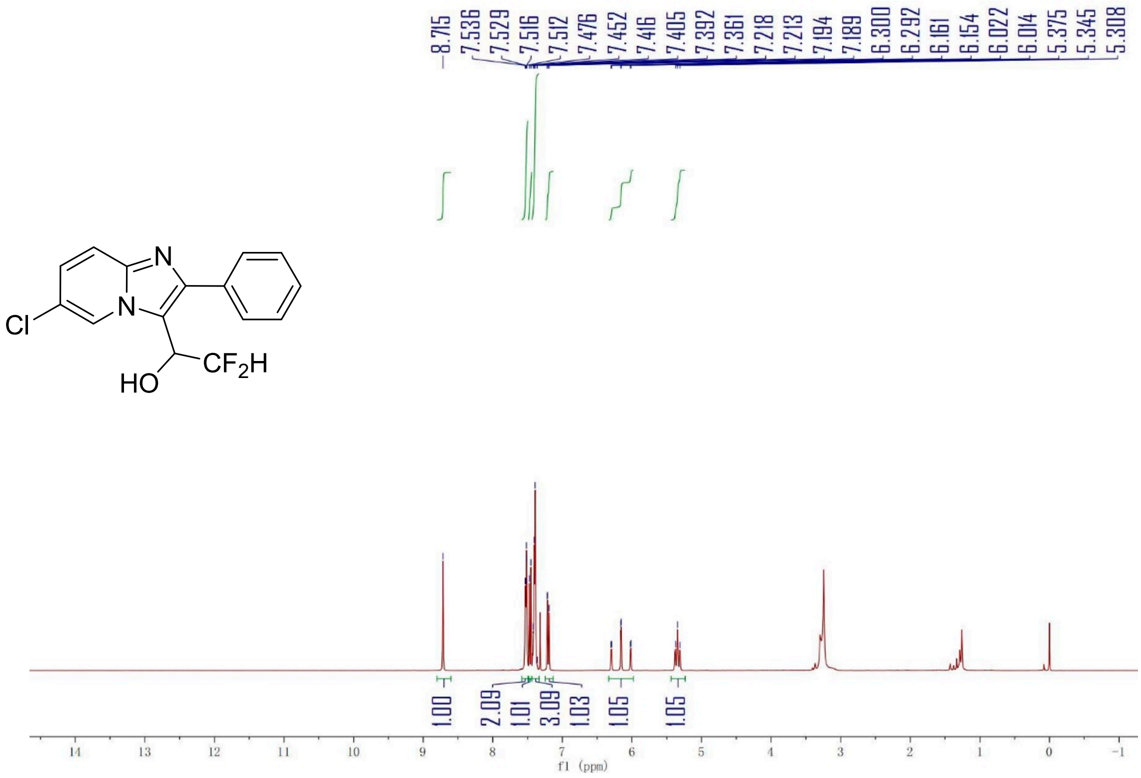

^{1}H NMR spectrum of compound **3ae**.

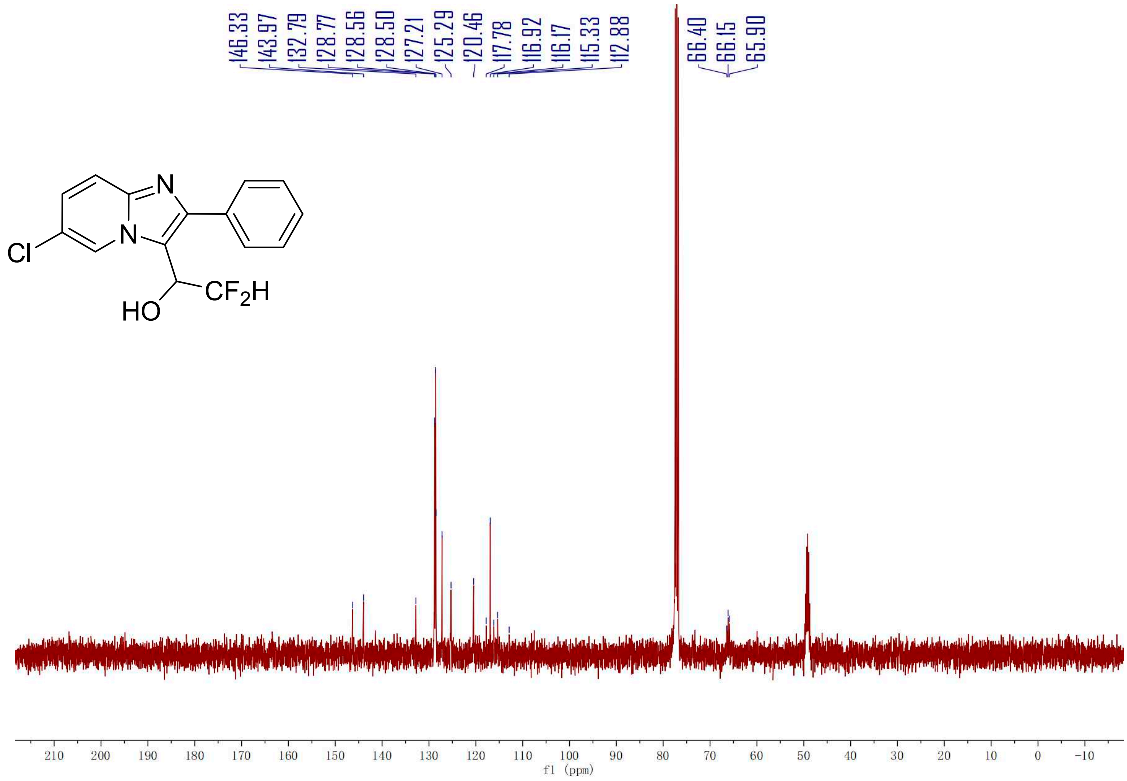

^{13}C NMR spectrum of compound **3ae**.

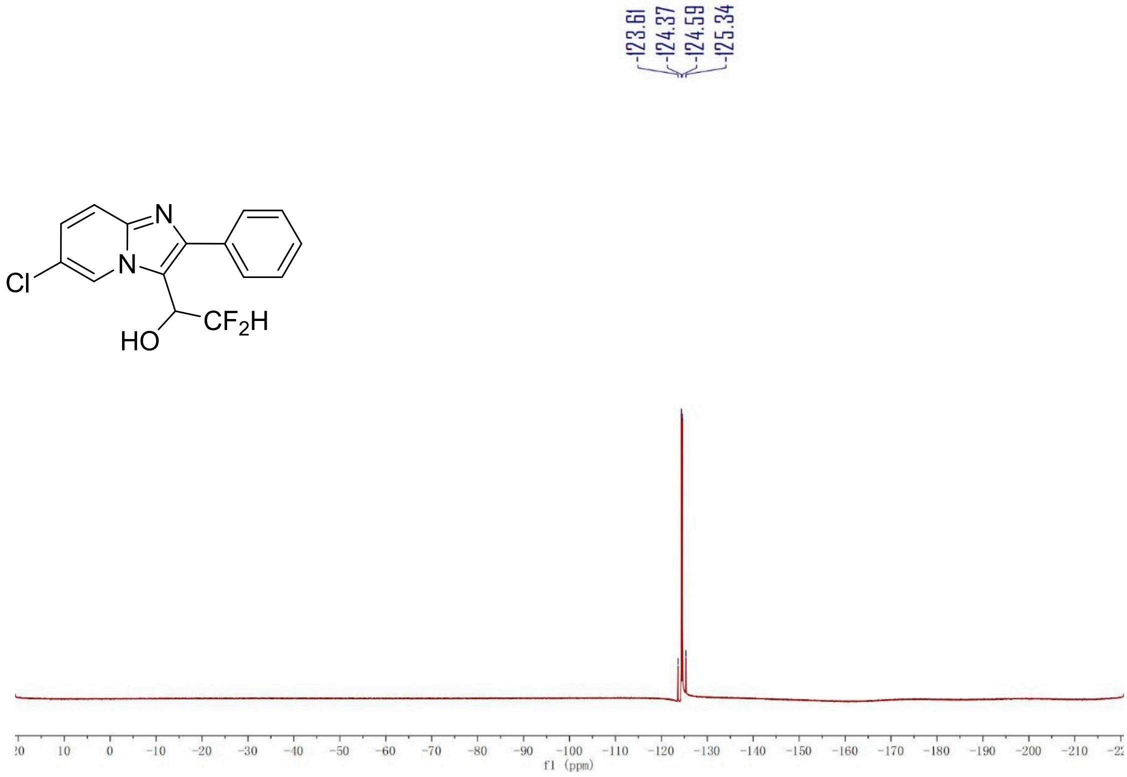

^{19}F NMR spectrum of compound **3ae**.

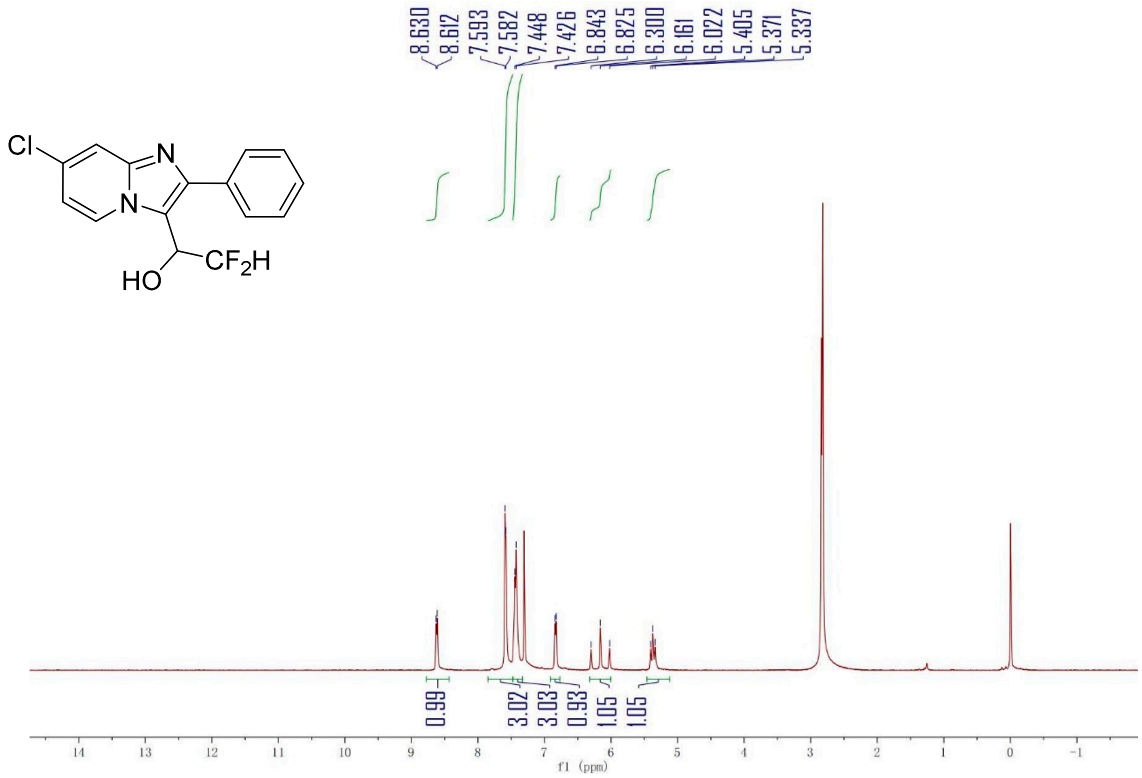

^{1}H NMR spectrum of compound **3af**.

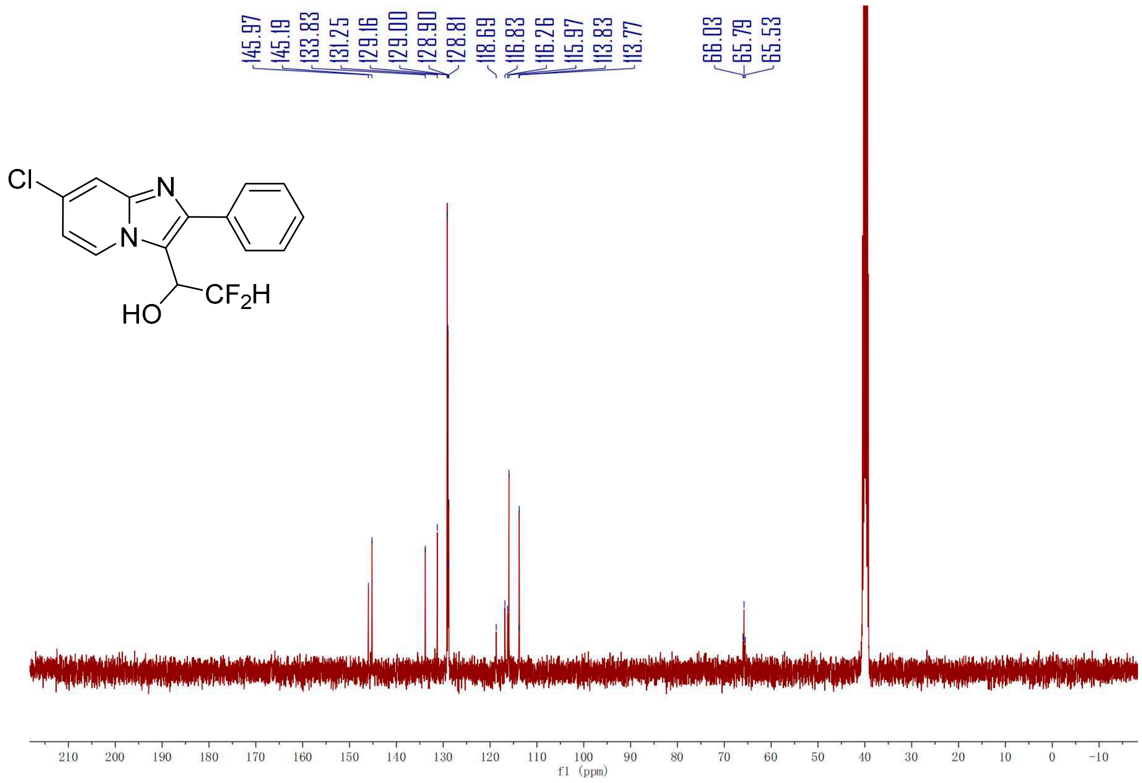

^{13}C NMR spectrum of compound **3af**.

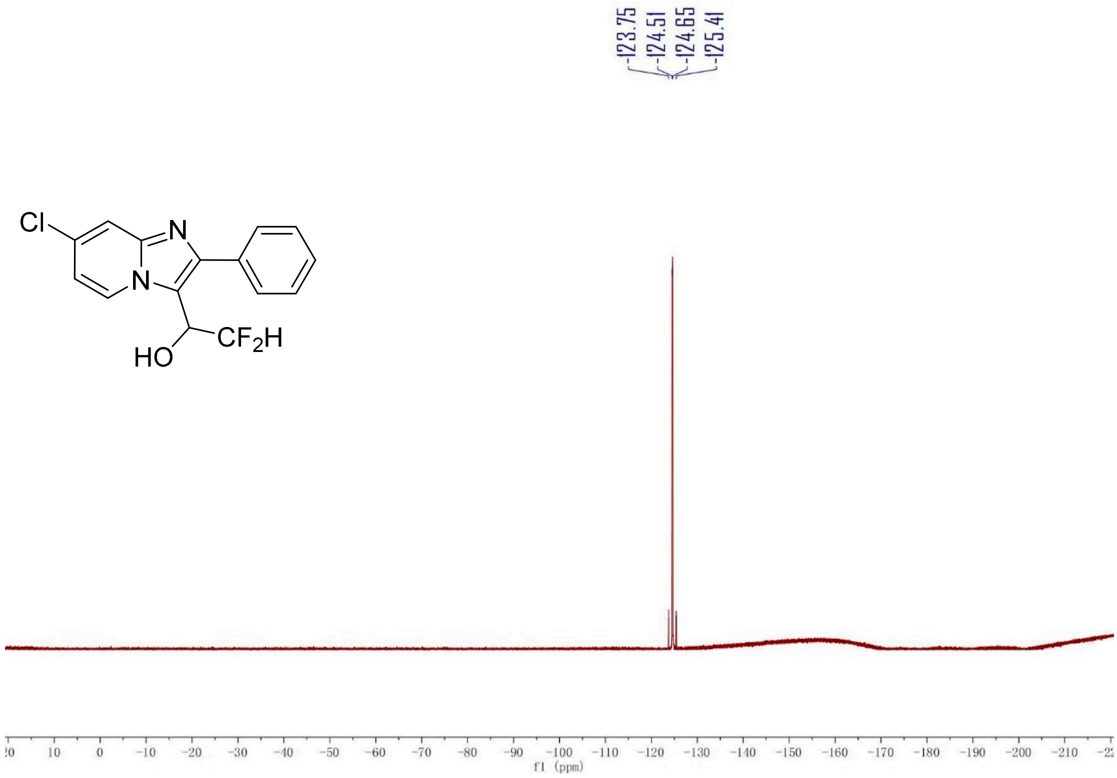

^{19}F NMR spectrum of compound **3af**.

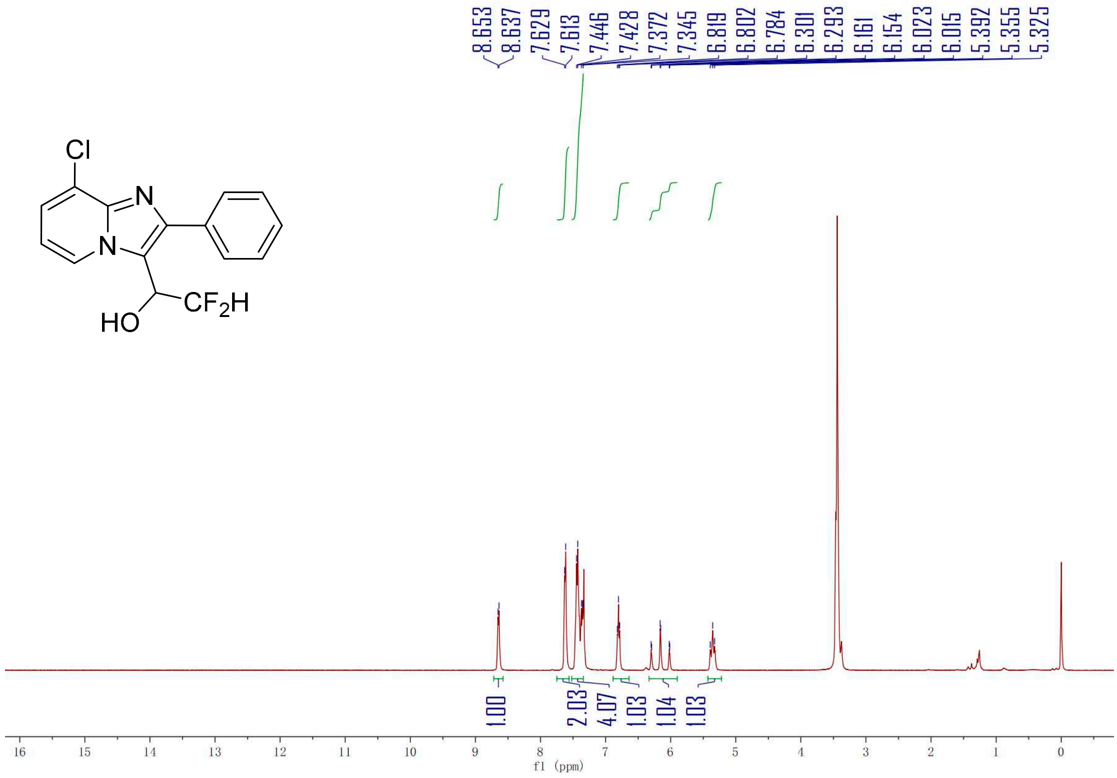

^{1}H NMR spectrum of compound **3ag**.

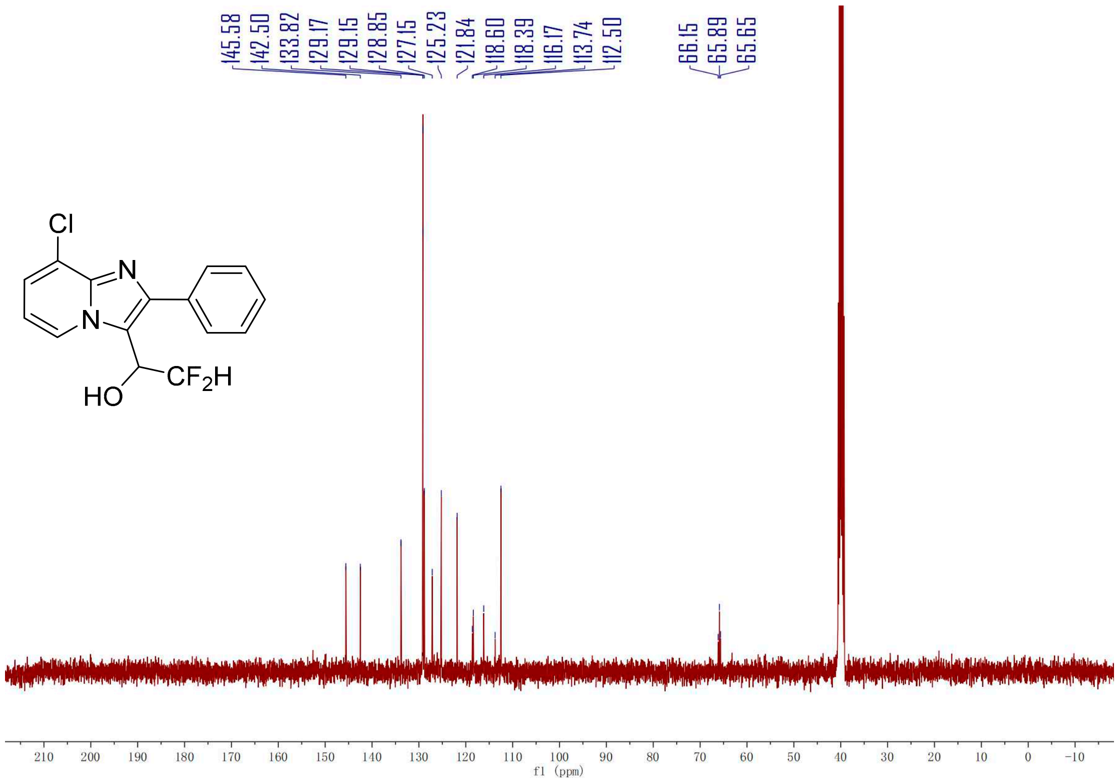

^{13}C NMR spectrum of compound **3ag**.

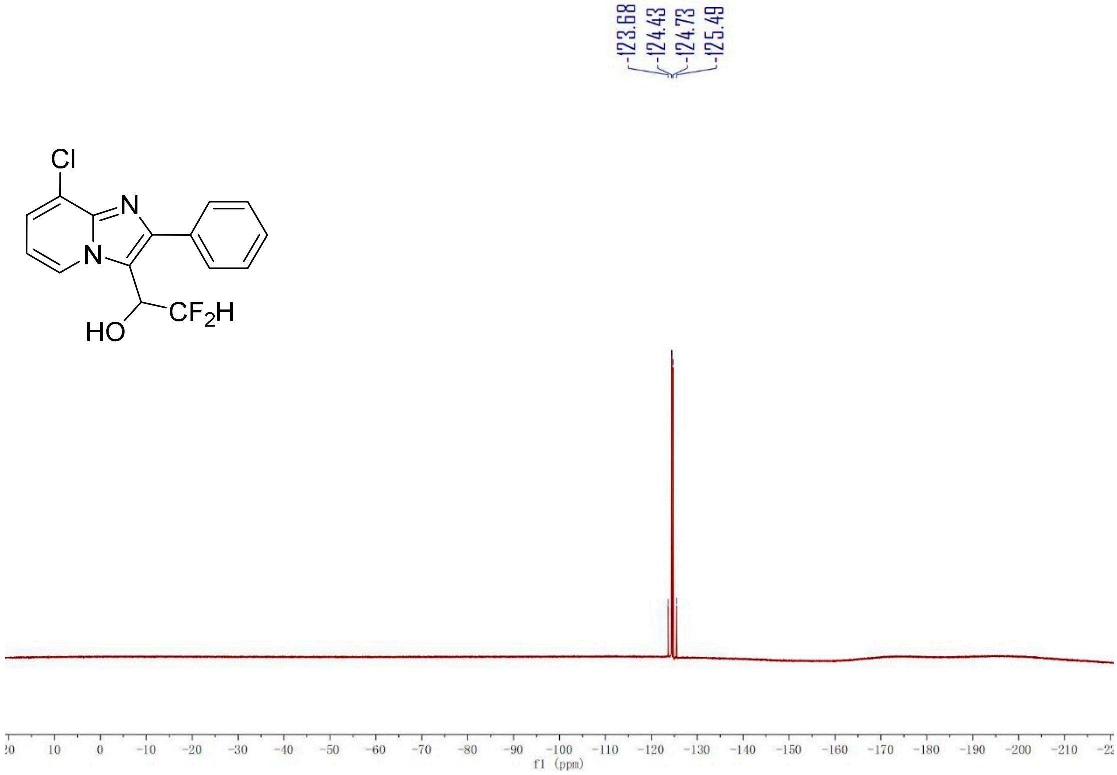

^{19}F NMR spectrum of compound **3ag**.

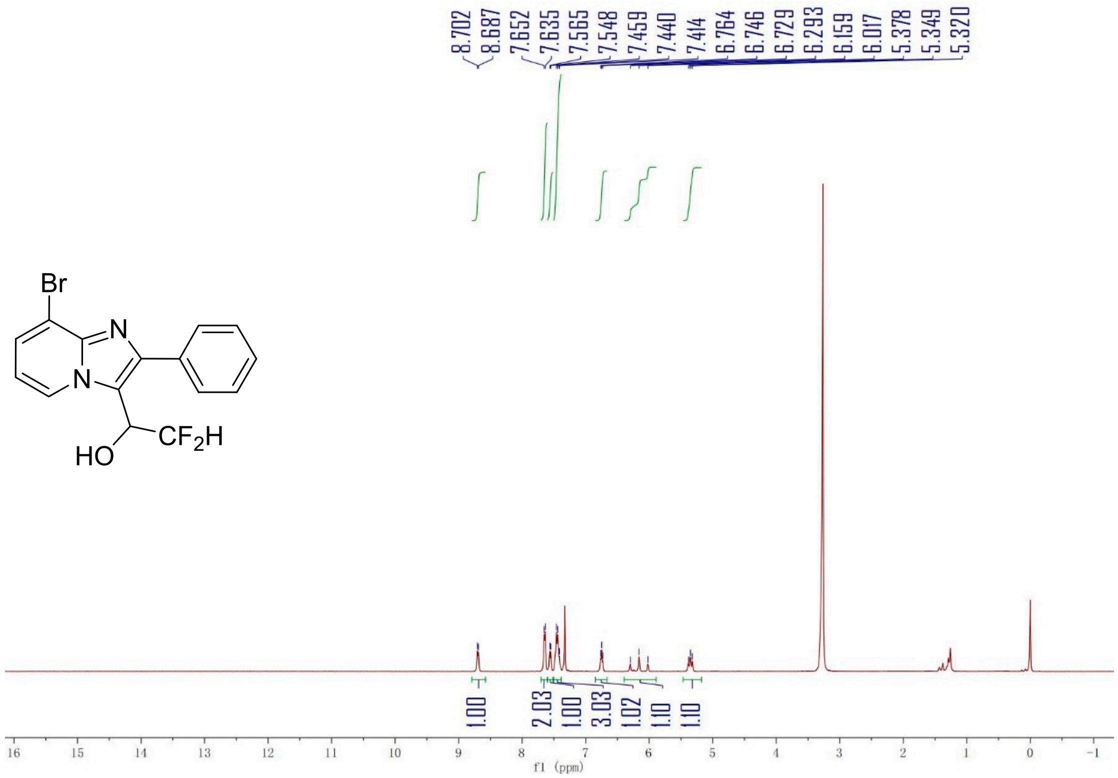

^{1}H NMR spectrum of compound **3ah**.

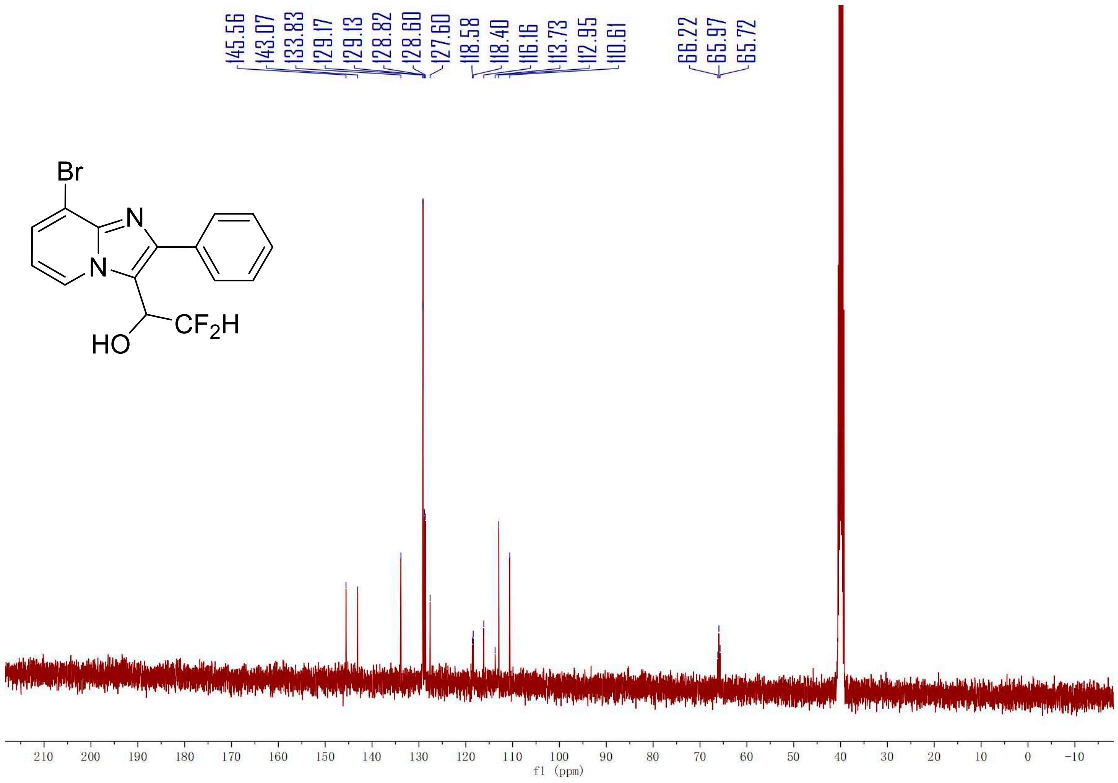

^{13}C NMR spectrum of compound **3ah**.

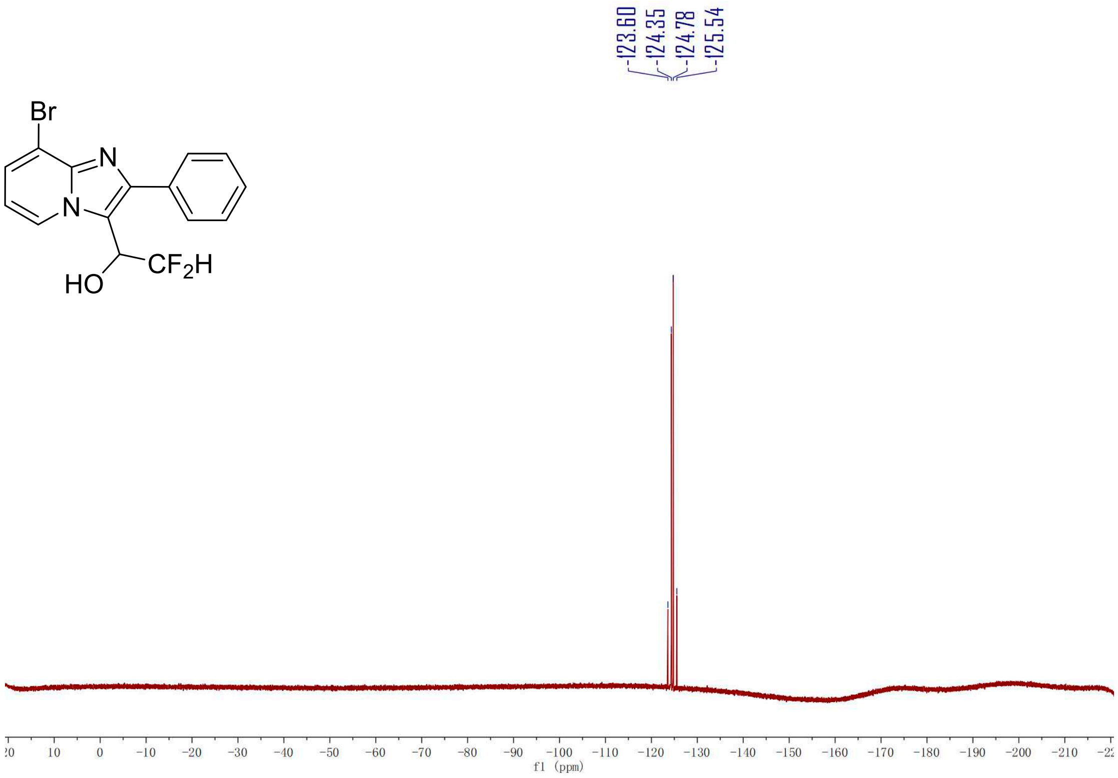

^{19}F NMR spectrum of compound **3ah**.

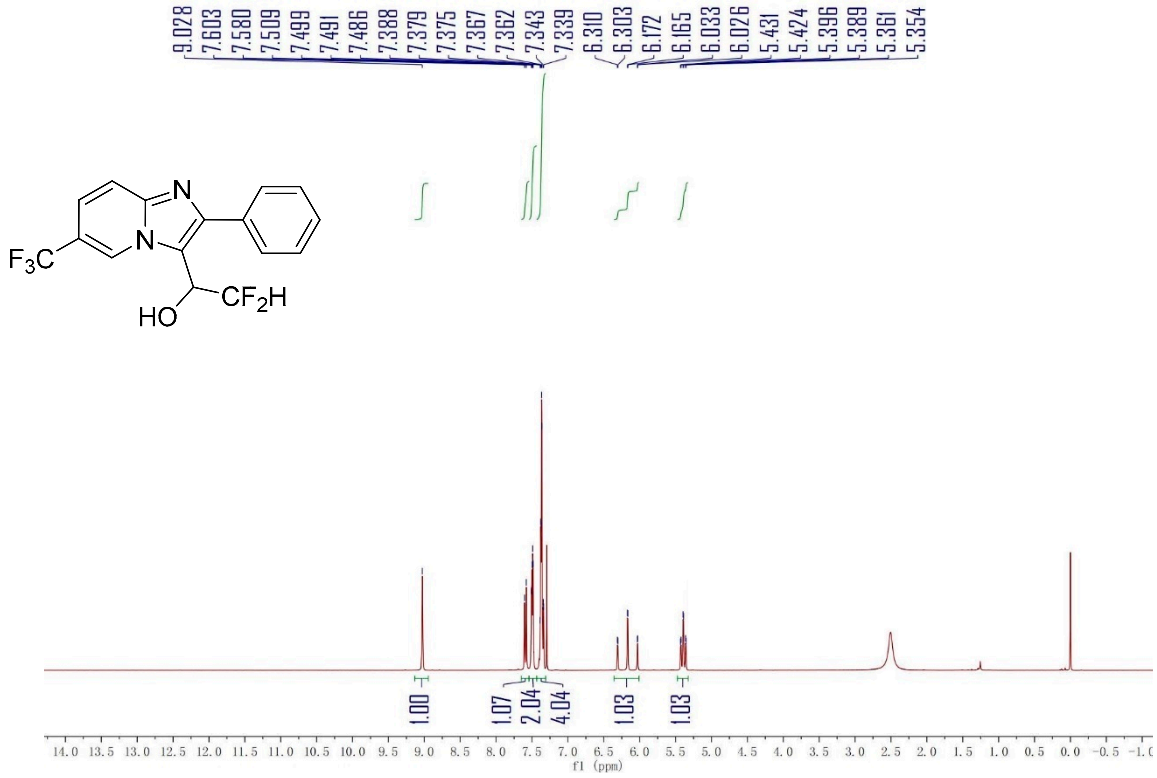

^{1}H NMR spectrum of compound **3ai**.

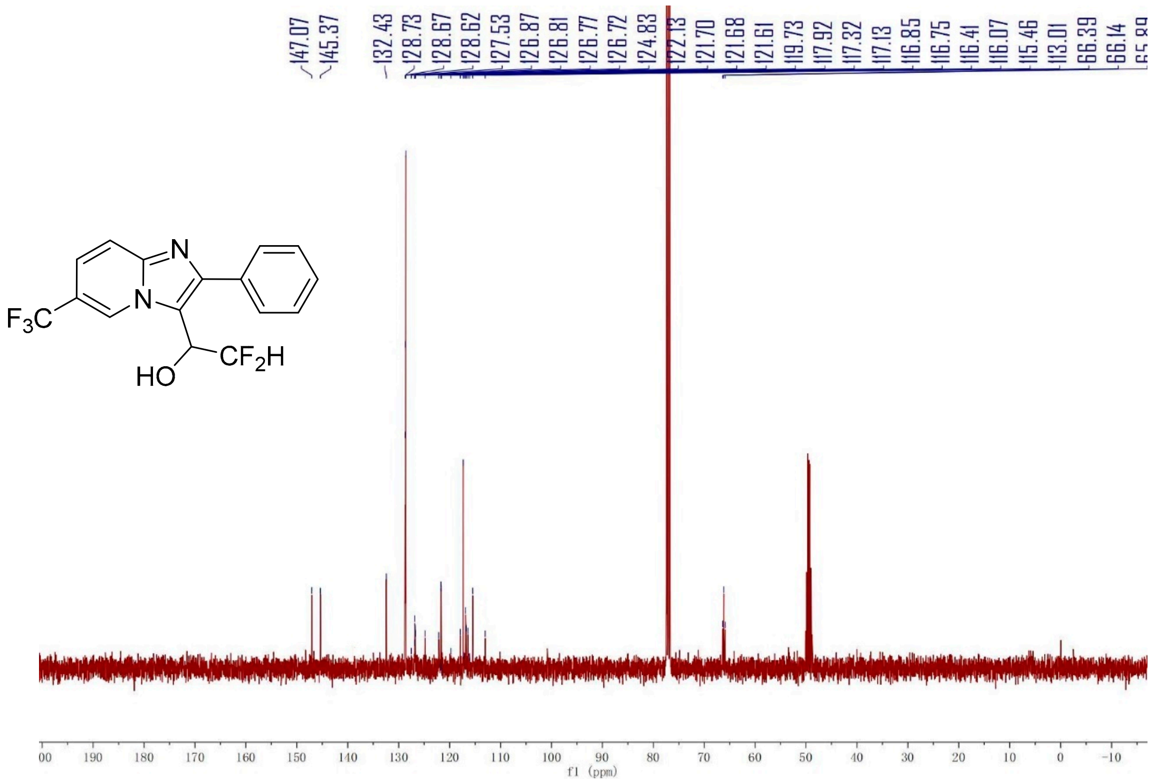

^{13}C NMR spectrum of compound **3ai**.

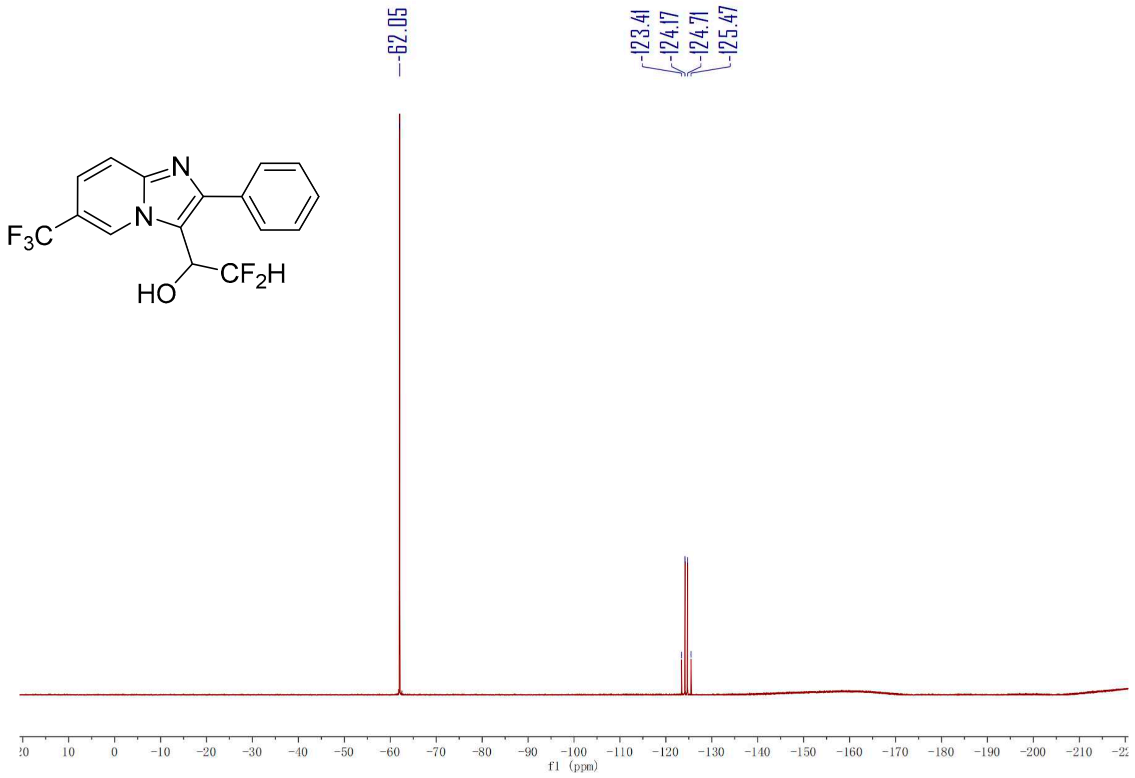

^{19}F NMR spectrum of compound **3ai**.

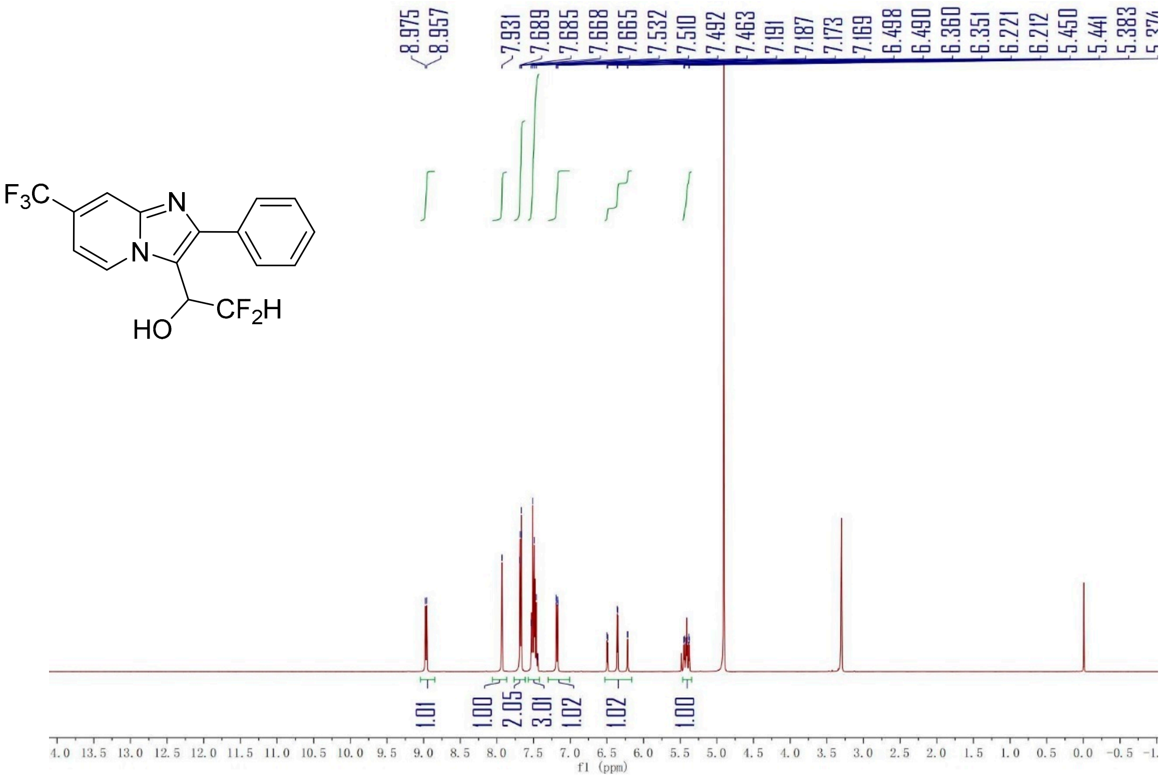

^{1}H NMR spectrum of compound **3aj**.

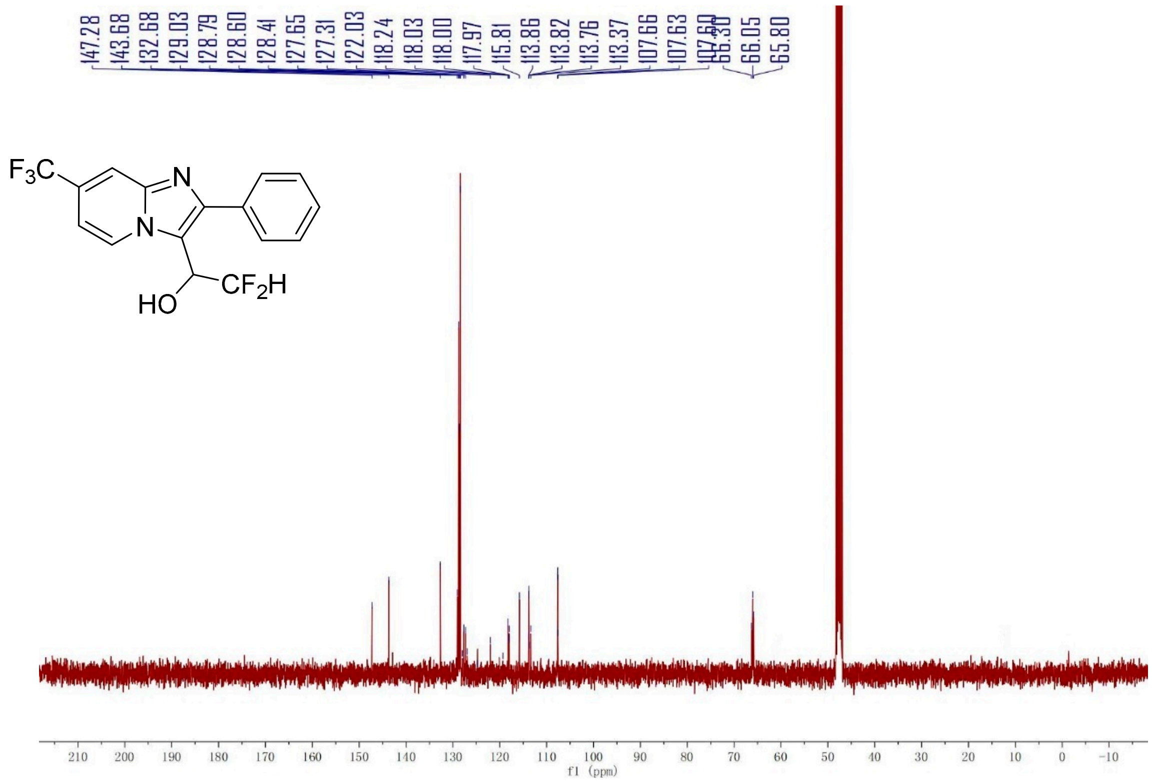

^{13}C NMR spectrum of compound **3aj**.

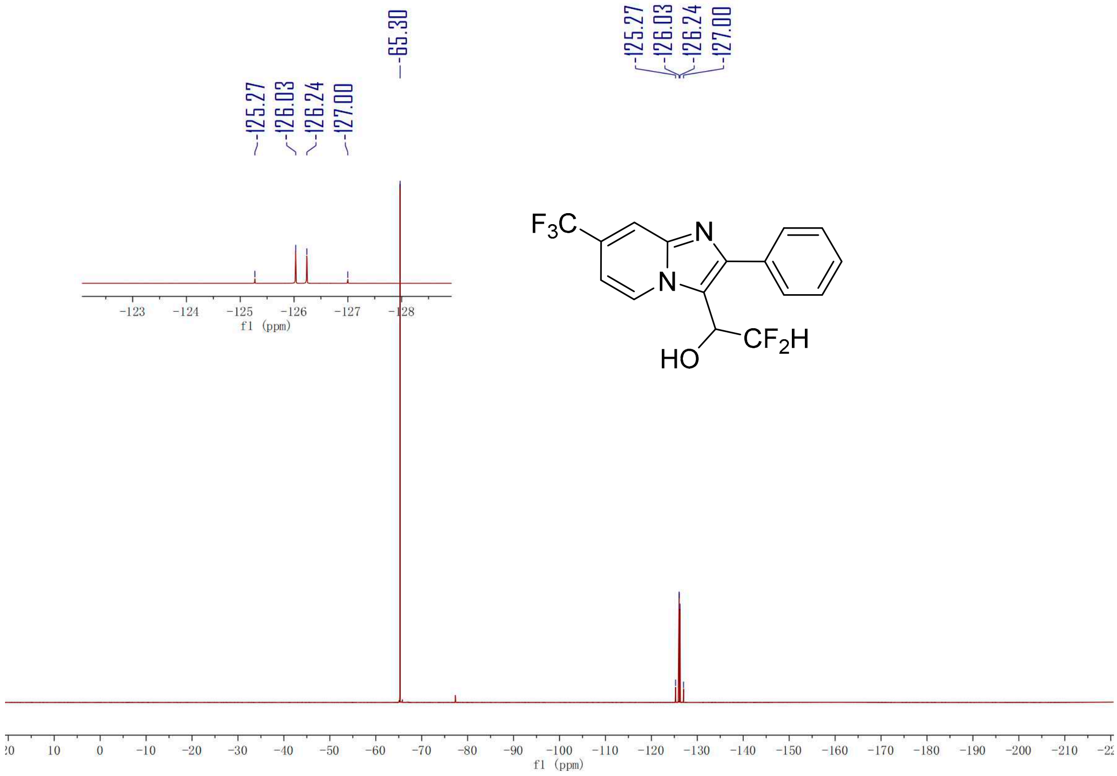

^{19}F NMR spectrum of compound **3j**.

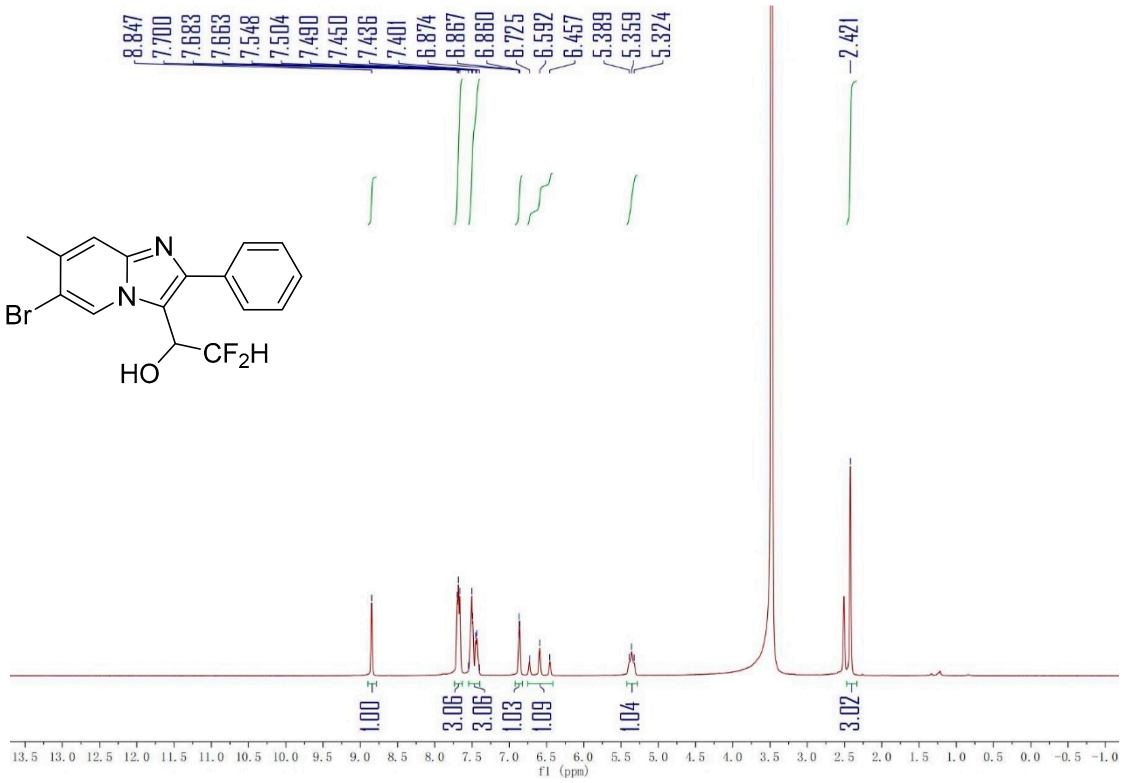

^{1}H NMR spectrum of compound **3ak**.

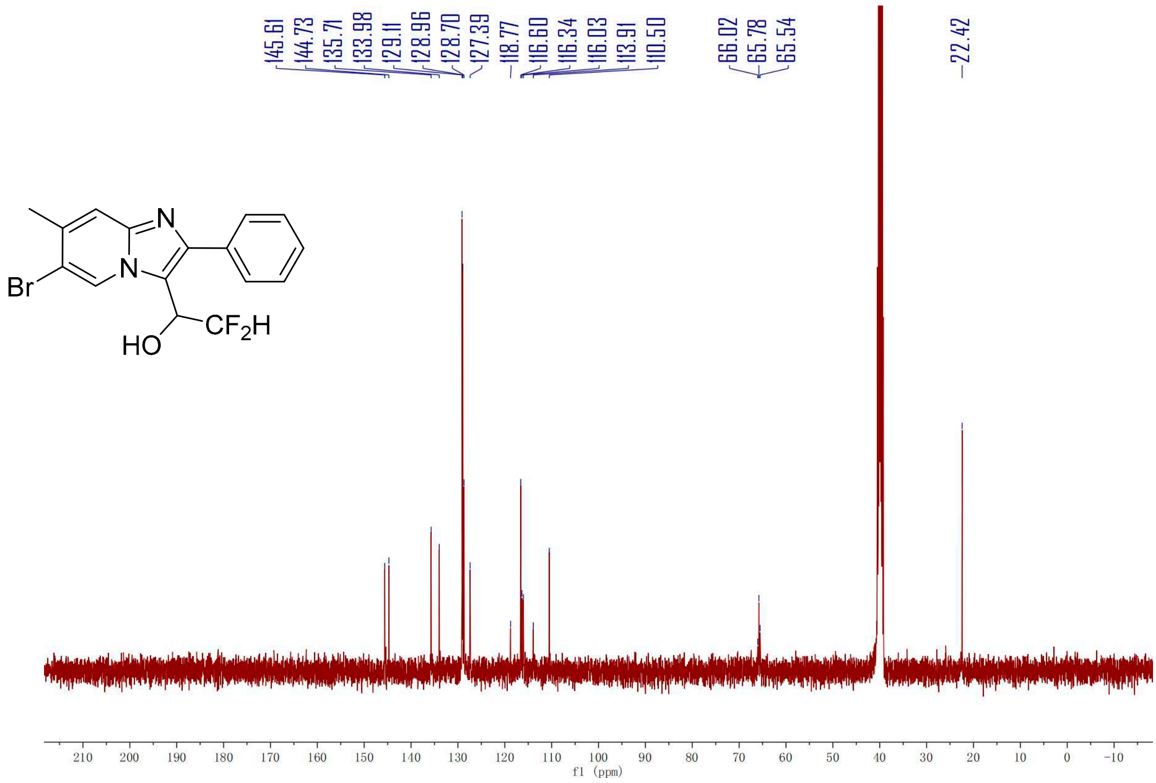

^{13}C NMR spectrum of compound **3ak**.

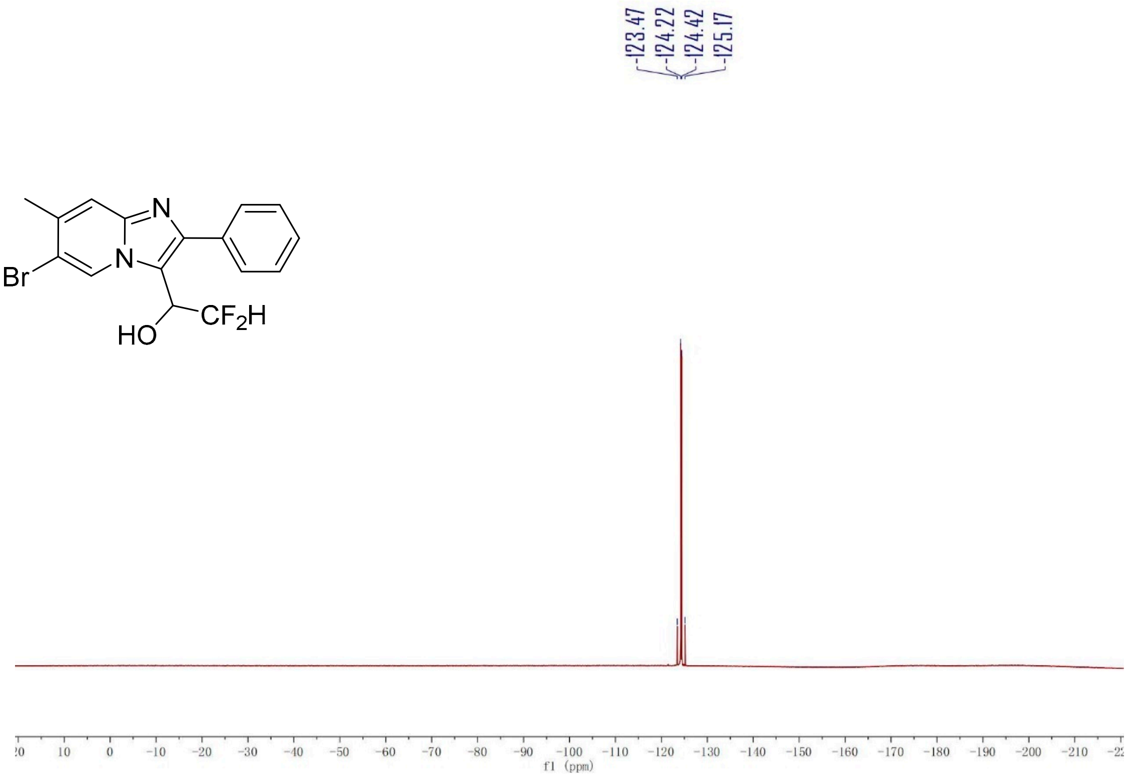

^{19}F NMR spectrum of compound **3ak**.

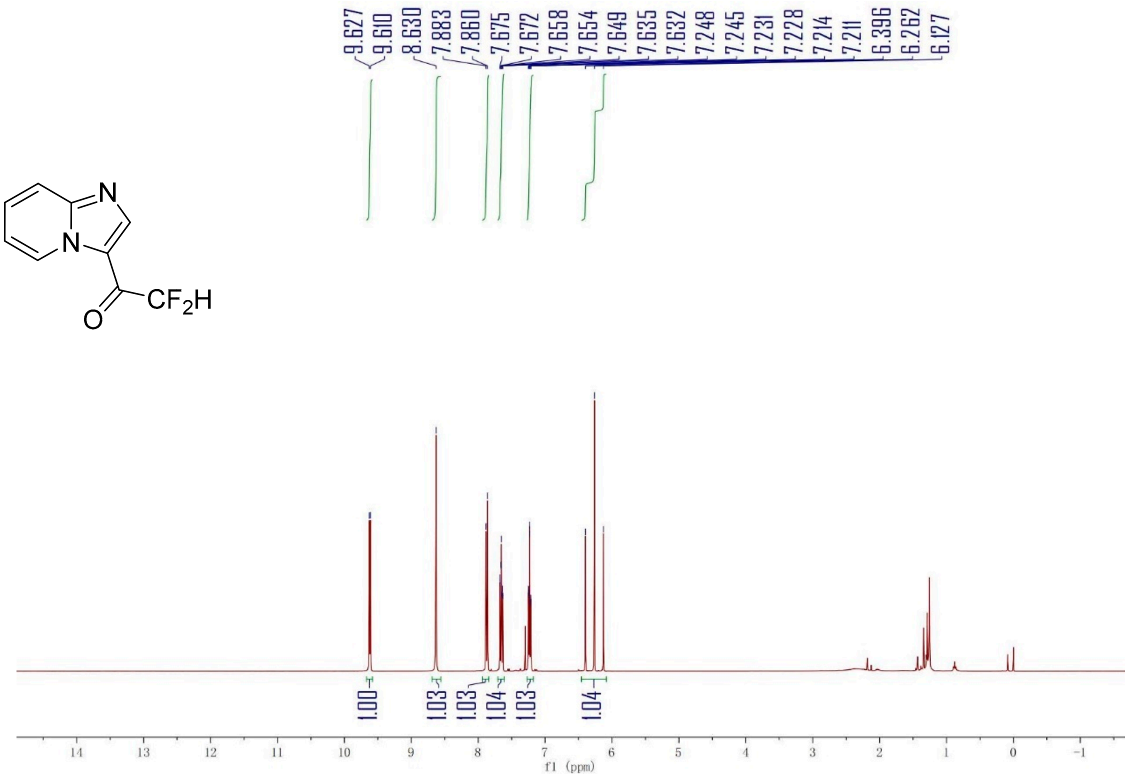

^{1}H NMR spectrum of compound **4a**.

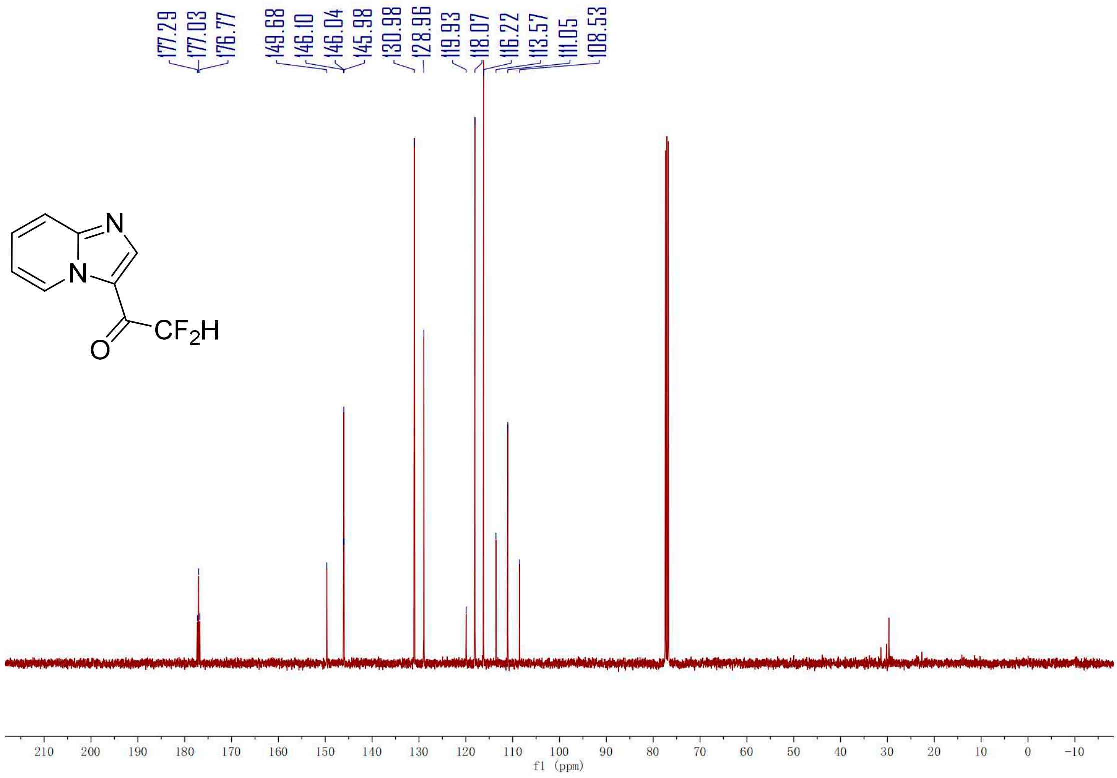

^{13}C NMR spectrum of compound **4a**.

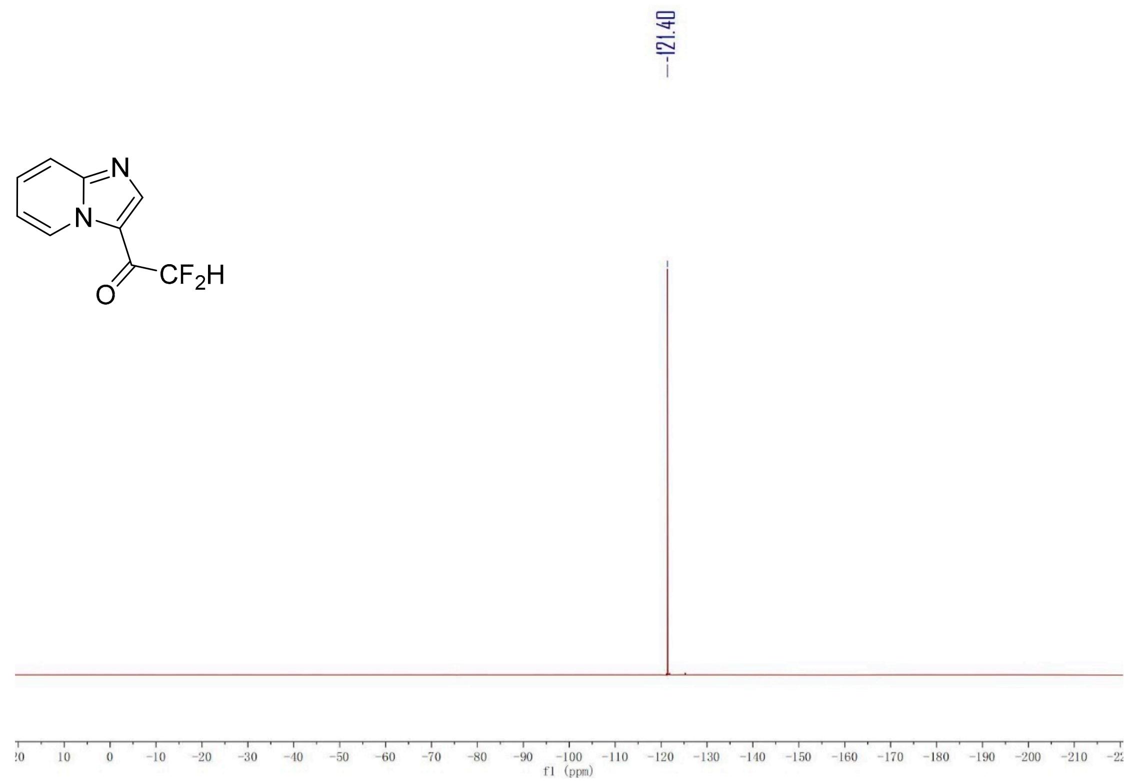

^{19}F NMR spectrum of compound **4a**.

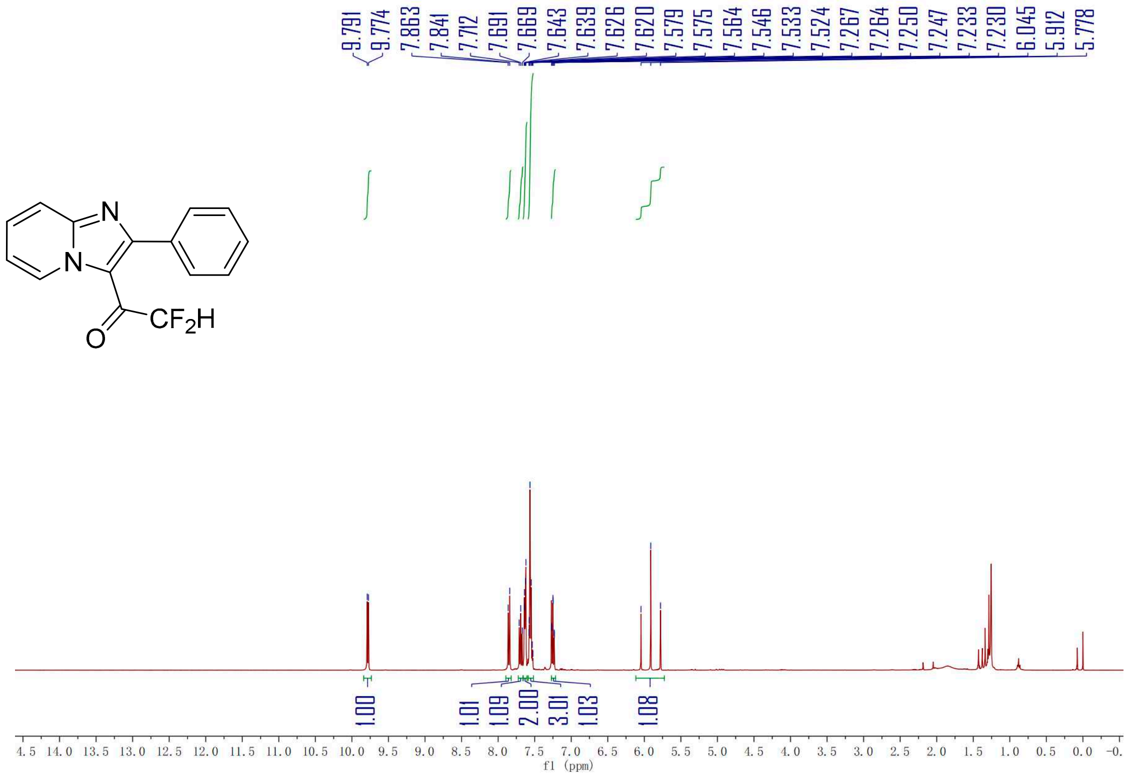

^{1}H NMR spectrum of compound **4b**.

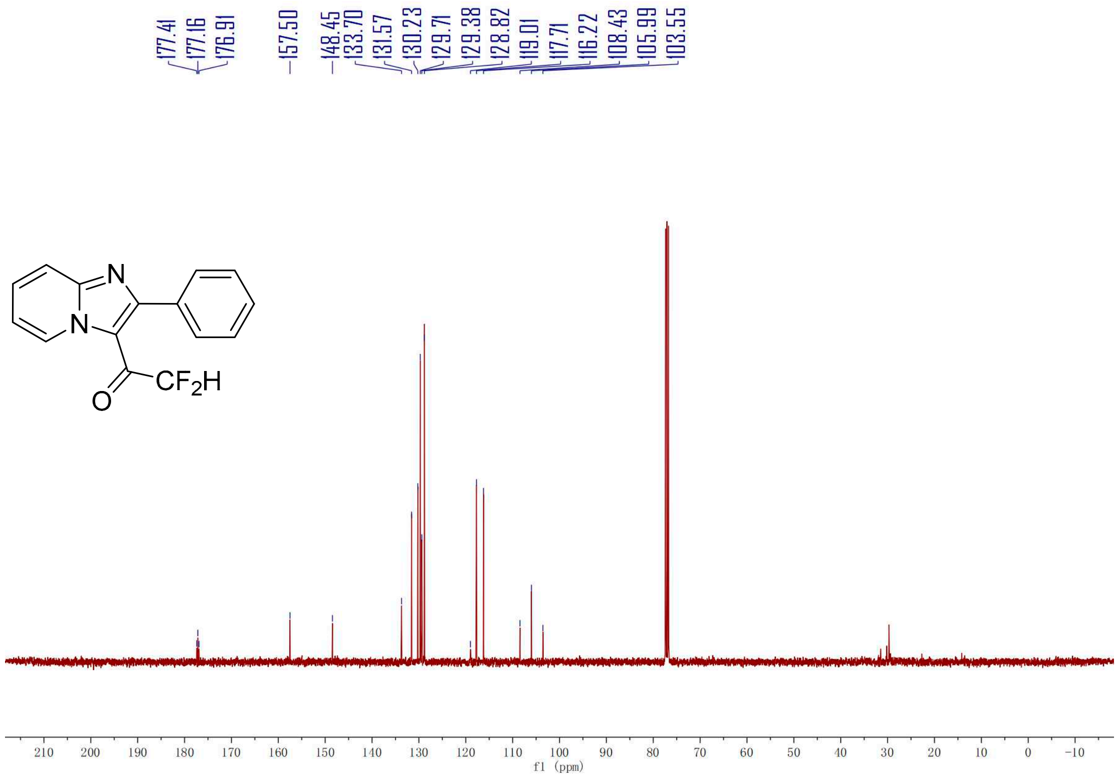

^{13}C NMR spectrum of compound **4b**.

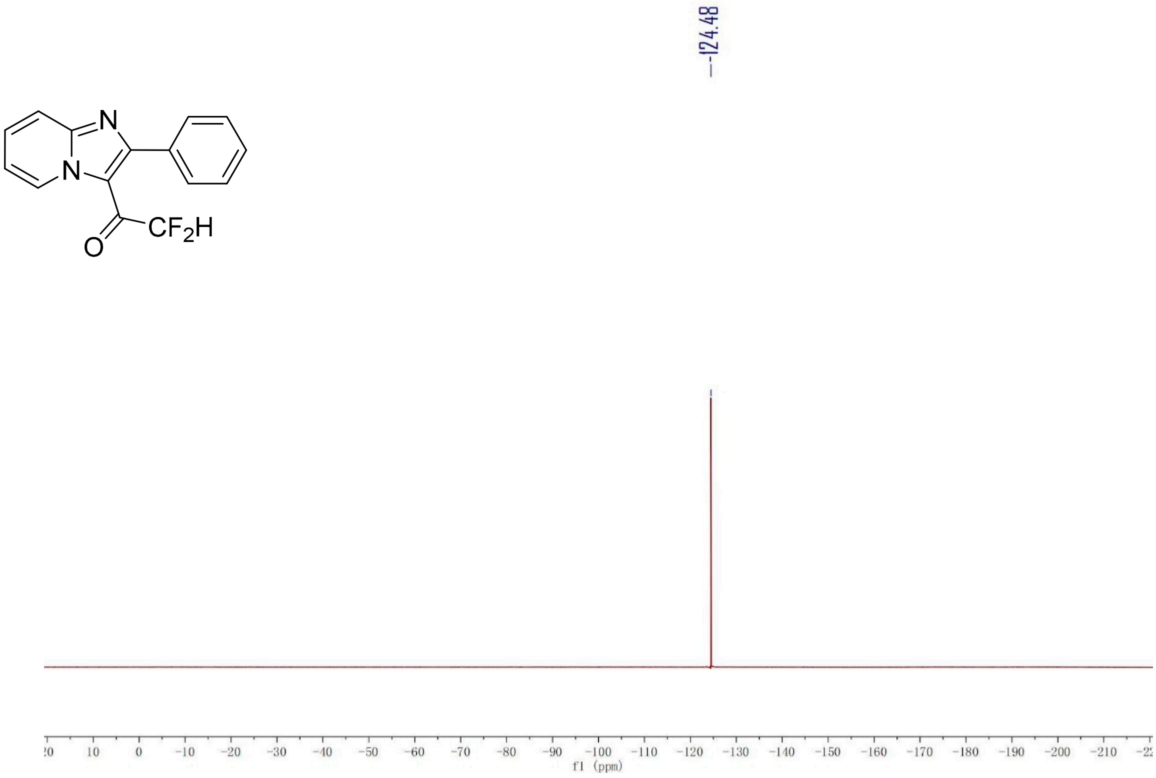

^{19}F NMR spectrum of compound **4b**.

References

[1] Nipate, D. S.; Jaspal, S.; Shinde, V. N.; Rangan, K.; Kumar, A. TEMPO-Mediated Cross-Dehydrogenative Coupling of Indoles and Imidazo[1,2-*a*]pyridines with Fluorinated Alcohols. *Org. Lett.*, **2021**, *23*, 1373.

[2] Ghosh, P.; Ganguly, B.; Kar, B.; Dwivedi, S.; Das, S. Green procedure for highly efficient, rapid synthesis of imidazo[1,2-*a*]pyridine and its late stage functionalization. *Synth. Commun.*, **2018**, *48*, 1076.
